# Humans and machines in biomedical knowledge curation: hypertrophic cardiomyopathy molecular mechanisms’ representation

**DOI:** 10.1186/s13040-021-00279-2

**Published:** 2021-10-02

**Authors:** Mila Glavaški, Lazar Velicki

**Affiliations:** 1 Faculty of Medicine, University of Novi Sad, Novi Sad, Serbia; 2 Institute of Cardiovascular Diseases Vojvodina, Sremska Kamenica, Serbia

**Keywords:** Data mining, Curation, Automated curation, Hypertrophic cardiomyopathy, Signaling pathways, Knowledge graphs, Disease maps

## Abstract

**Background:**

Biomedical knowledge is dispersed in scientific literature and is growing constantly. Curation is the extraction of knowledge from unstructured data into a computable form and could be done manually or automatically. Hypertrophic cardiomyopathy (HCM) is the most common inherited cardiac disease, with genotype–phenotype associations still incompletely understood. We compared human- and machine-curated HCM molecular mechanisms’ models and examined the performance of different machine approaches for that task.

**Results:**

We created six models representing HCM molecular mechanisms using different approaches and made them publicly available, analyzed them as networks, and tried to explain the models’ differences by the analysis of factors that affect the quality of machine-curated models (query constraints and reading systems’ performance). A result of this work is also the Interactive HCM map, the only publicly available knowledge resource dedicated to HCM. Sizes and topological parameters of the networks differed notably, and a low consensus was found in terms of centrality measures between networks. Consensus about the most important nodes was achieved only with respect to one element (calcium). Models with a reduced level of noise were generated and cooperatively working elements were detected. REACH and TRIPS reading systems showed much higher accuracy than Sparser, but at the cost of extraction performance. TRIPS proved to be the best single reading system for text segments about HCM, in terms of the compromise between accuracy and extraction performance.

**Conclusions:**

Different approaches in curation can produce models of the same disease with diverse characteristics, and they give rise to utterly different conclusions in subsequent analysis. The final purpose of the model should direct the choice of curation techniques. Manual curation represents the gold standard for information extraction in biomedical research and is most suitable when only high-quality elements for models are required. Automated curation provides more substance, but high level of noise is expected. Different curation strategies can reduce the level of human input needed. Biomedical knowledge would benefit overwhelmingly, especially as to its rapid growth, if computers were to be able to assist in analysis on a larger scale.

**Supplementary Information:**

The online version contains supplementary material available at 10.1186/s13040-021-00279-2.

## Background

Biomedical knowledge is dispersed across scientific papers and databases and is growing constantly. Biomedical literature can be seen as a large, unstructured data repository [1]. PubMed is a biomedical literature database and supports the search and retrieval of the literature [2]. Filters are used to narrow the search by different criteria (publication date, species, etc.). Each publication in the database has a unique PubMed Identifier (PMID). Medical Subject Headings (MeSH) is a vocabulary thesaurus used for indexing articles for PubMed [3]. Combinations of these and other approaches (e.g., using keywords and key phrases) can be used to constrain database queries. There are also other biomedical databases such as Pathway Commons [4], DrugBank [5], ChEMBL [6], CTDbase [7], miRTarBase [8], and many more.

Curation is the extraction of knowledge from unstructured data into a structured, computable form [9]. Molecular mechanisms can be extracted from biomedical knowledge resources by manual or automated curation [10, 11]. Manual curation consists of the synthesis and integration of information from the literature, large-scale projects, and databases [9] and represents the gold standard for information extraction in biomedical research [12]. The extracted information about molecular mechanisms can be subsequently visually represented using visual pathway editors such as CellDesigner [10]. One example of an automated approach is the “Integrated Network and Dynamical Reasoning Assembler” (INDRA), which extracts molecular mechanisms from text and biomedical databases and assembles them into executable models [13]. It contains a number of clients for accessing and using resources from biomedical databases (e.g., Pathway Commons database) and literature clients for retrieving the literature. For the extraction of molecular mechanisms from text, INDRA uses reading systems such as REACH [14], TRIPS [15], Sparser [16], ISI [17], RLIMPS-P [18], Eidos [19], etc. They extract INDRA statements, intermediate knowledge representations of extracted molecular mechanisms [13]. INDRA statements are then assembled into models [13]. The INDRA Database is built with INDRA, combining content from numerous readers and databases [20].

When the information is combined, its value increases [9]. Disease maps are comprehensive, knowledge-based representations of disease mechanisms [21]. Biomedical knowledge in the form of graphs facilitates the study of complex processes, both as visual and thereby more intuitive representations, as well as a standardized data structure that is human- and computer-readable [22].

Hypertrophic cardiomyopathy (HCM) is the most common genetic cardiac disease [23–25], with a prevalence of 1 in 500 people worldwide [23, 26–29]. It is characterized by marked variability in expression, ranging from asymptomatic to sudden cardiac death or heart failure [30]. In addition to the direct effects of underlying mutations, gene expression is altered by micro and small noncoding RNAs, and secondary molecular changes occur in many signaling pathways [31]. Many studies have been conducted to decipher the molecular mechanisms underlying HCM; however, genotype–phenotype associations remain incompletely understood [32].

Models made exclusively by manual curation or by automated curation have never been compared. Automated biomedical knowledge curation policies that produce disease models of higher quality are still not known.

Our aims were to compare human- and machine-curated HCM models, as well as to examine the performance of different machine approaches for the same task.

## Results

### Constructed models

We created six models representing HCM molecular mechanisms using different approaches and made them publicly available (Table 1). The Manual HCM model was constructed by a human, based on an extensive literature search in PubMed, using CellDesigner. The Tabular manual HCM model was created by manual transcription of species and reactions from the original Manual HCM model CellDesigner XML file to nodes and interactions of a network table in XLSX format. The INDRA-assembled PubMed HCM model was assembled automatically, using INDRA’s PubMed literature client. The INDRA-assembled PubMed+PathwayCommons HCM model was assembled automatically, using INDRA’s PubMed literature client and Pathway Commons database via INDRA’s BioPAX API. The Truncated INDRA DB model was created using INDRA Database. Only statements that were completely correctly extracted from the text were incorporated into the Truncated INDRA DB model. After applying the criteria for correctness, 9.27% of statements remained for inclusion in the Truncated INDRA DB HCM model. The INDRA DB model was created using the INDRA Database. All statements returned by the query were incorporated into the INDRA DB model.

**Table 1:** Constructed models

Model	Number of elements	Number of interactions	Number of compartments	Available at
Manual HCM model	440^a^	509^a^	0^a^	https://bit.ly/3s47FyA
Tabular manual HCM model	175	278	0	https://bit.ly/3saXwR2
INDRA-assembled PubMed HCM model	435	451	0	https://bit.ly/3blm2rB
INDRA-assembled PubMed+PathwayCommons HCM model	1883	3642	0	https://bit.ly/2OLxJQM
Truncated INDRA DB HCM model	77	59	0	https://bit.ly/2ZKypbD
INDRA DB HCM model	546	638	0	https://bit.ly/3upHsga

^a^As estimated by Cytoscape. The original Manual HCM model consisted of 207 elements, 233 reactions, and 11 compartments

The number of elements and interactions in models differ markedly, regardless of whether they represent the same disease (HCM). Models created by automated curation contain no compartments (Table 1).

### Network analysis of the generated models

#### Topological analysis

Topological parameters for the networks (Table 2) and network diameter per element (Table 3) were computed.

**Table 2:** Topological parameters for HCM models obtained with Network Analyzer

	Manual HCM model	Tabular manual HCM model	INDRA-assembled PubMed HCM model	INDRA-assembled PubMed+PathwayCommons HCM model	Truncated INDRA DB HCM model	INDRA DB HCM model
Average number of neighbors	2.309^a^	2.789	1.917	3.582	1.455	2.059
Network diameter	1^a^	12	6	8	3	9
Network radius	1^a^	1	1	1	1	1
Characteristic path length	1.000^a^	4.334	2.541	2.395	1.299	3.900
Clustering coefficient	0.000^a^	0.054	0.007	0.006	0.014	0.028
Network density	0.003^a^	0.009	0.002	0.001	0.010	0.002
Connected components	26^a^	11	58	51	23	101
Multi-edge node pairs	1^a^	24	21	213	3	48
Number of self-loops	0^a^	4	7	14	0	6

^a^ Due to the CellDesigner XML file incompatibility, we suggest that some or all topological measures for the Manual HCM model are calculated falsely by Cytoscape

**Table 3:** Network diameter per element

	Manual HCM model	Tabular manual HCM model	INDRA-assembled PubMed HCM model	INDRA-assembled PubMed+PathwayCommons HCM model	Truncated INDRA DB HCM model	INDRA DB HCM model
Network diameter/number of elements	0.0023^a^ 0.0048	0.0686	0.0138	0.0042	0.0390	0.0165

^a^Number of elements estimated using Cytoscape

#### Nodes’ centrality scores

**Fig. 1:**
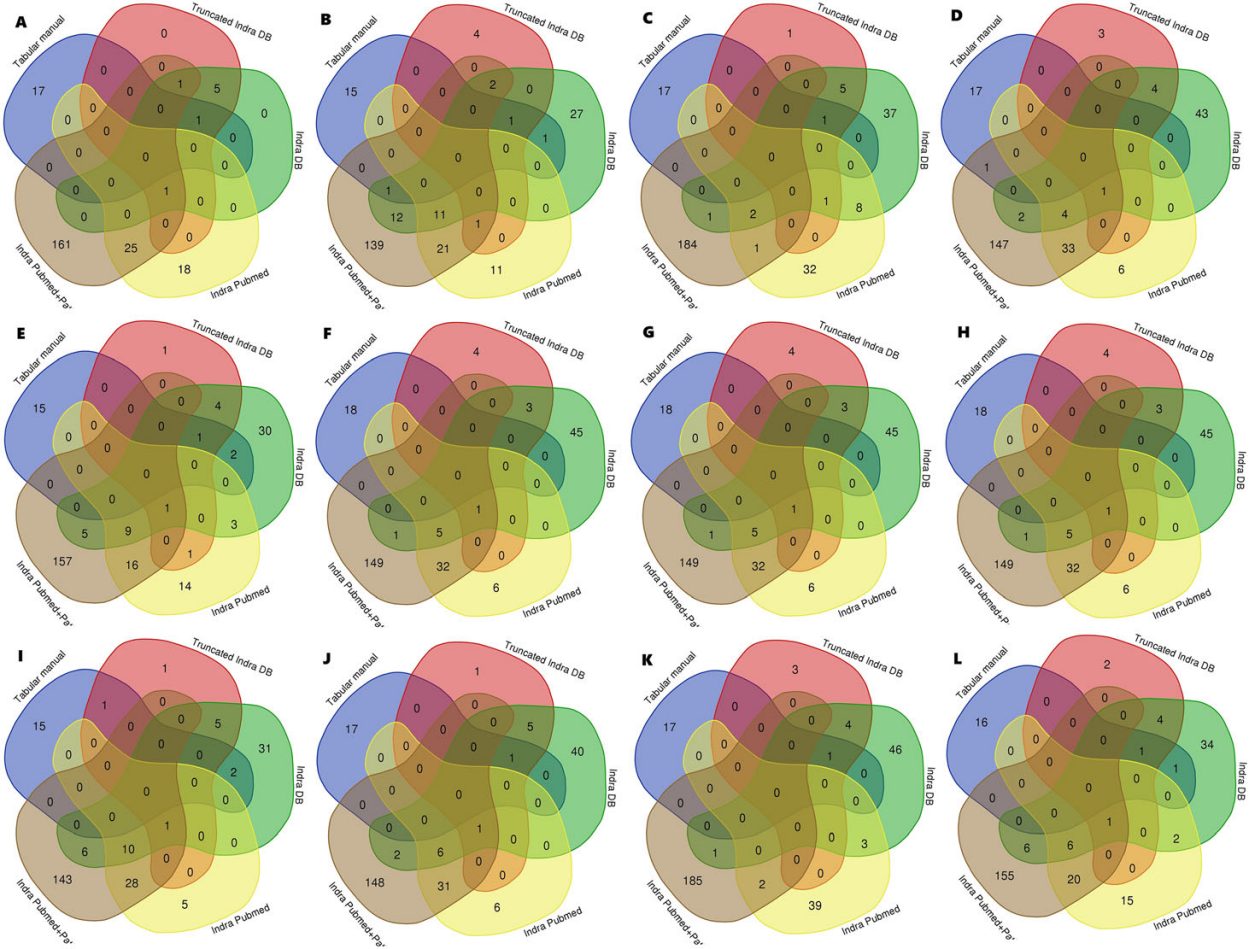
Intersections of sets containing top 10% elements ranked by centrality measures for each network. Top 10% elements were determined for each network by: **a**-betweenness, **b**-bottleneck, **c**-closeness, **d**-clustering coefficient, **e**-degree, **f**-DMNC, **g**-eccentricity, **h**-EPC, **i**-MCC, **j**-MNC, **k**-radiality, **l**-stress

The intersections of sets containing the top 10% elements by centrality measures for each network showed low consensus in terms of centrality measures between networks (Fig. 1). The elements ranked in the top 10% by different centrality measures for each network were visualized (Table 4). Network centrality scores could not be determined for the CellDesigner XML file.

**Table 4:** Elements ranked as top 10% by centrality measures for each network

Model	Link to folder with top 10% elements for each of centrality measures for the model
Tabular manual HCM model	https://bit.ly/3s7PQyO
INDRA-assembled PubMed HCM model	https://bit.ly/3k6Dmon
INDRA-assembled PubMed+PathwayCommons HCM model	https://bit.ly/3s9Wc0x
Truncated INDRA DB HCM model	https://bit.ly/3s6uqSL
INDRA DB model	https://bit.ly/37Kqlfc

#### The most important nodes

**Fig. 2:**
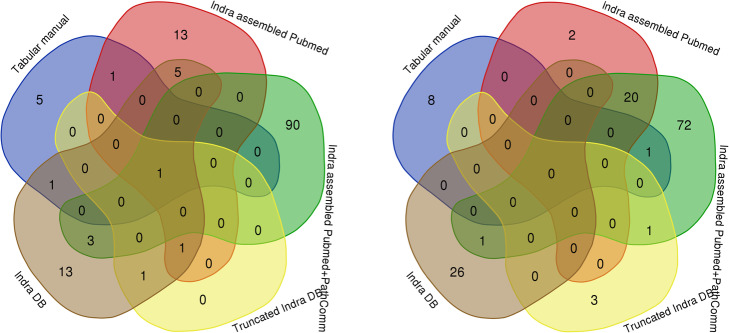
The most important elements of networks (left) and the least important elements of networks (right)

Consensus about the most important nodes was achieved only with respect to one element (calcium), while consensus for other most and least important nodes was lacking (Fig. 2).

**Fig. 3:**
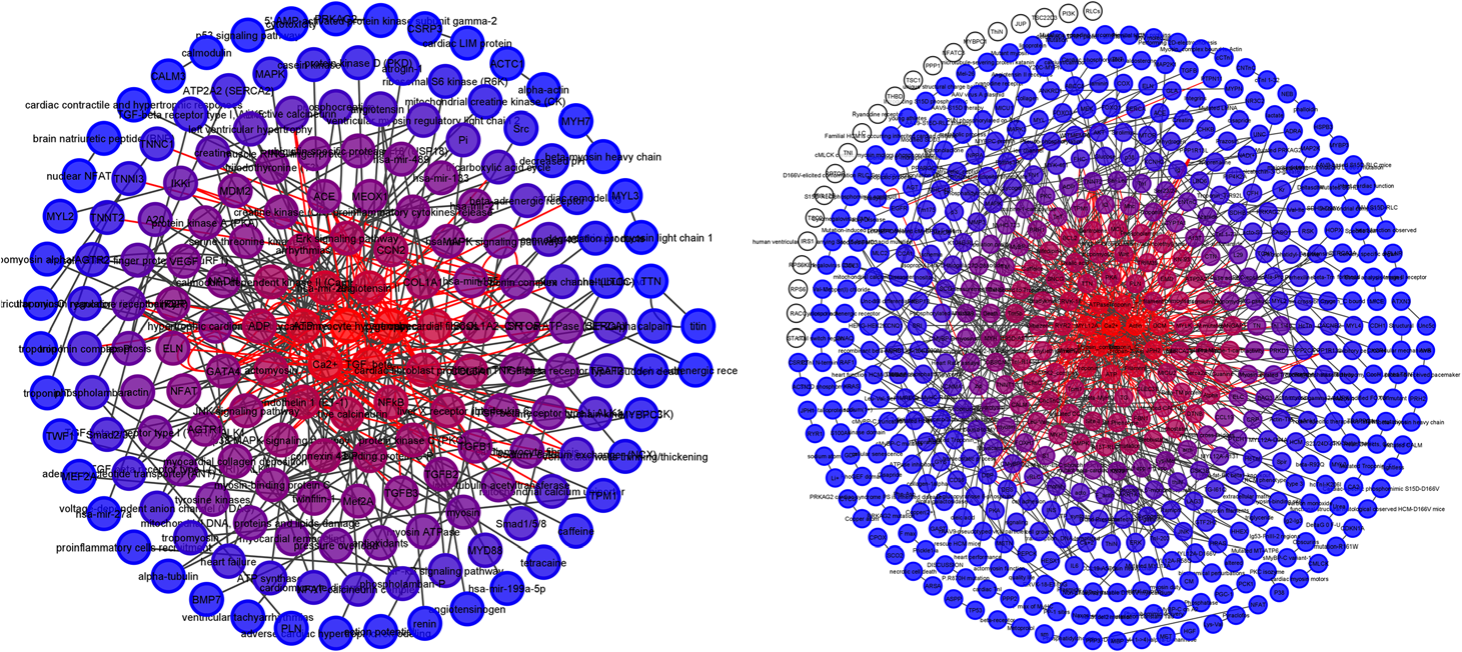
Packed concentric ring sorted by k-shell and gradient of nodes’ color. Tabular manual HCM model (left), INDRA DB model (right)

Each network was represented as a packed concentric ring sorted by k-shell and gradient of nodes’ color applied based on k-shell (Fig. 3, Additional file 1). Rank and k-shell for each node of each network were calculated (Additional file 2). Cytoscape Wk-decomposition [33] could not be performed on the CellDesigner XML file.

#### Reliability of interactions

A different level of reliability threshold was estimated and applied for each model and, as a result, models with reduced levels of noise were generated (Table 5).

**Table 5:** Estimated best reliability threshold for each network and models with reduced level of noise

Model	Estimated best reliability threshold	Models with reduced level of noise
Manual HCM model	–	https://bit.ly/3qDFZ3g
Tabular manual HCM model	0.15	https://bit.ly/3qBzv59
INDRA-assembled PubMed HCM model	0.15	https://bit.ly/3bBKFkf
INDRA-assembled PubMed+PathwayCommons HCM model	0.60	https://bit.ly/3s6ALO3
Truncated INDRA DB HCM model	0.02	https://bit.ly/3k9iH2T
INDRA DB model	0.50	https://bit.ly/3pFqo1Y

#### Cooperatively working elements

The number of detected cooperatively working elements (functional modules) was vastly different for networks (Table 6). Models made by machines without later human intervention contained ambiguous and exogenous elements in the detected functional modules (Table 6, Additional file 3). We have proposed likely implications for the detected functional modules in HCM (Additional file 3). The Manual HCM model could not be analyzed using NCMine app [34].

**Table 6:** Functional modules

Model	Criterion for near-clique mining	Number of functional modules detected	Functional modules with ambiguous elements (%)	Functional modules with exogenous elements (%)
Tabular manual HCM model	Page Rank	17	0.00	0.00
Tabular manual HCM model	Node Degree	18	0.00	0.00
INDRA-assembled PubMed HCM model	Page Rank	6	50.00	16.67
INDRA-assembled PubMed HCM model	Node Degree	5	60.00	20.00
INDRA-assembled PubMed+PathwayCommons HCM model	Page Rank	61	4.92	77.05
INDRA-assembled PubMed+PathwayCommons HCM model	Node Degree	60	5.00	80.00
Truncated INDRA DB HCM model	Page Rank	2	0.00	0.00
Truncated INDRA DB HCM model	Node Degree	2	0.00	0.00
INDRA DB HCM model	Page Rank	27	22.22	18.52
INDRA DB HCM model	Node Degree	33	21.21	15.15

### Factors that affect the quality of machine-curated models

#### Query constraints in machine-curated models

Query based on keywords is considerably more potent than query by MeSH (Table 7).

**Table 7:** Number of results as a consequence of different query constraints

Query		Filter	Search details	Number of results
MeSH	Cardiomyopathy, Hypertrophic, Familial	10 years	MeSH Term	265
MeSH	Cardiomyopathy, Hypertrophic, Familial	10 years	MeSH Major Topic	232
keywords	familial hypertrophic cardiomyopathy	10 years	–	562
keywords	“familial hypertrophic cardiomyopathy”	10 years	Exact match	336
keywords	hypertrophic cardiomyopathy	10 years	–	7952
keywords	“hypertrophic cardiomyopathy”	10 years	Exact match	7390

The average year of publication for papers found by INDRA Database [20] query by the MeSH, used for the INDRA DB HCM model, was x̅=2010.27, with 43.75% of the papers describing research conducted on human material, 15.97% on human and other species material, and the rest being animal studies.

#### Reading systems’ performance

**Fig. 4:**
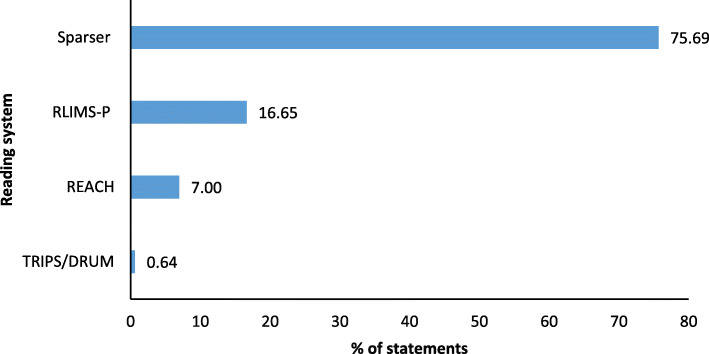
Reading systems’ contribution to extraction of statements for INDRA DB HCM model

**Fig. 5:**
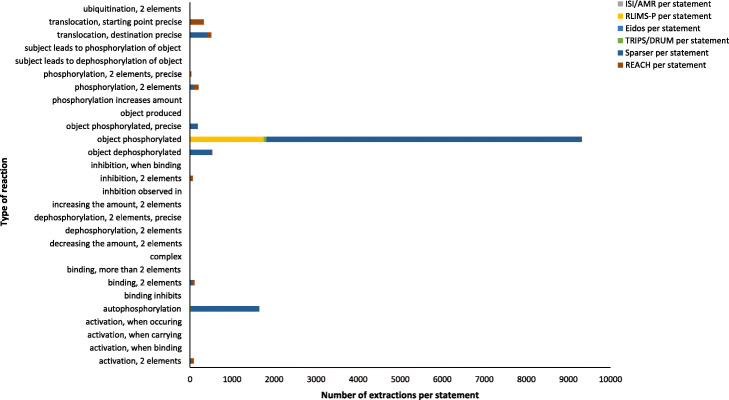
Number of extractions per statement for 28 reaction types in INDRA DB HCM model

The most dominant reading system for the extraction of statements for the INDRA DB HCM model was Sparser, followed by RLIMS-P, REACH, and TRIPS/DRUM (Fig. 4). Reading systems’ extraction performance differed markedly for different reaction types (Table 8). Most extractions per statement were found for different versions of phosphorylation and translocation (Fig. 5).

**Table 8:** Percent of reading systems’ extractions by different reaction types in INDRA DB HCM model

Reaction types	ISI/AMR (%)	RLIMS-P (%)	Eidos (%)	TRIPS/DRUM (%)	Sparser (%)	REACH (%)
Activation, 2 elements	0.01	0.00	0.20	0.05	22.57	77.16
Activation, when binding	0.00	0.00	0.00	0.00	0.00	100.00
Activation, when carrying	0.00	0.00	100.00	0.00	0.00	0.00
Activation, when occurring	0.00	0.00	100.00	0.00	0.00	0.00
Autophosphorylation	0.00	0.00	0.00	1.57	98.43	0.00
Binding inhibits	0.00	0.00	0.00	0.00	0.00	100.00
Binding, 2 elements	0.04	0.00	0.00	0.03	58.36	41.57
Binding, more than 2 elements	0.00	0.00	0.00	0.00	99.07	0.93
Complex	0.00	0.00	100.00	0.00	0.00	0.00
Decreasing the amount, 2 elements	0.00	0.00	0.00	0.00	0.00	100.00
Dephosphorylation, 2 elements	0.00	0.00	0.00	0.00	0.00	100.00
Dephosphorylation, 2 elements, precise	0.00	0.00	0.00	0.00	0.00	100.00
Increasing the amount, 2 elements	0.00	0.00	0.00	0.00	0.00	100.00
Inhibition observed in	0.00	0.00	100.00	0.00	0.00	0.00
Inhibition, 2 elements	0.01	0.00	0.53	0.17	3.27	96.02
Inhibition, when binding	0.00	0.00	0.00	0.00	0.00	100.00
Object dephosphorylated	0.00	0.00	0.00	0.00	99.95	0.05
Object phosphorylated	0.00	18.80	0.00	0.71	80.25	0.24
Object phosphorylated, precise	0.00	9.15	0.00	0.00	90.79	0.06
Object produced	0.00	0.00	0.00	60.00	0.00	40.00
Phosphorylation increases amount	0.00	0.00	0.00	0.00	0.00	100.00
Phosphorylation, 2 elements	0.05	5.08	0.00	0.23	43.26	51.38
Phosphorylation, 2 elements, precise	0.00	1.51	0.00	0.00	43.22	55.28
Subject leads to dephosphorylation of object	0.00	0.00	0.00	0.00	0.00	100.00
Subject leads to phosphorylation of object	0.00	0.00	0.00	0.00	0.00	100.00
Translocation, destination precise	0.00	0.00	0.00	0.08	82.95	16.97
Translocation, starting point precise	0.00	0.00	0.00	0.00	0.00	100.00
Ubiquitination, 2 elements	0.00	0.00	0.00	0.00	0.00	100.00

**Fig. 6:**
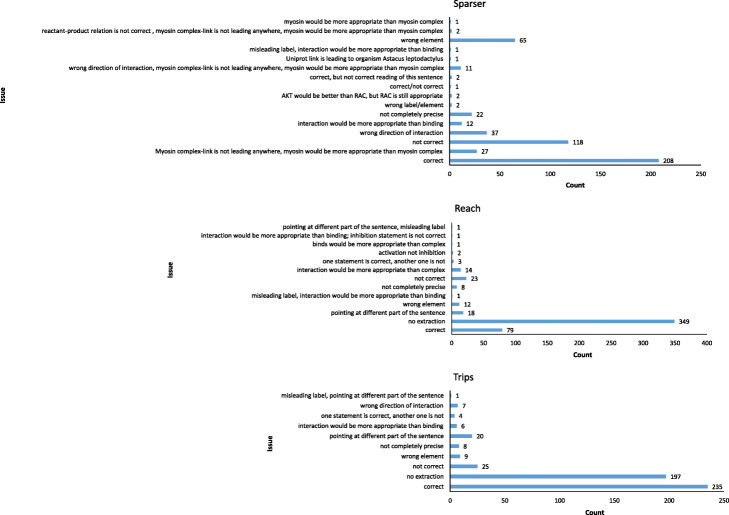
Specific issues found in the statements extracted by reading systems. Count of correct statements is shown as a reference point. The “not correct” issue was assigned in cases where two or more critical issues were found. Wrong element, misleading element label, wrong interaction, wrong direction of the interaction were designated as critical issues

For all reading systems, the most common issue was that statements extracted had two or more critical issues (a combination of wrong elements, misleading element label, wrong interaction, or wrong direction of the interaction) in the same statement, followed by wrong element and wrong direction of interaction in case of Sparser and TRIPS reading systems (Fig. 6).

REACH and TRIPS showed much higher accuracy than Sparser (Table 9) but at the cost of extraction performance (Fig. 4, Table 9). The TRIPS reading system proved to be the best single reading system for text segments about HCM when considering a compromise between accuracy and extraction performance (Fig. 4, Table 9).

**Table 9:** Accuracy of Sparser, REACH, and TRIPS reading systems

	Sparser	REACH	TRIPS
Tolerably accurate^a^ (%)	41.02	83.59	84.38
Not tolerably accurate, not inaccurate (%)	12.89	8.01	6.64
Inaccurate^b^ (%)	46.09	8.40	8.98
No extraction (%)	–	68.16	38.48

Accuracy has been determined for all text segments for which Sparser, as the most dominant reading system, extracted a statement. ^a^ Tolerably accurate: correct statement or no extraction; ^b^ Inaccurate: contains critical issue(s)

For the INDRA DB model, 44.19% of the statements extracted by the Eidos reading system (the result of 20.65% of total extractions by Eidos) were meaningless and inapplicable (Additional file 4). Those were complex statements by structure and brought puzzling noise to the model. For the statements representing simple interactions (consisting of one subject, one object, and interaction between them), Eidos extracted the possible and applicable statements.

### Interactive HCM map

The Interactive HCM map is available at https://silicofcm.eu/interactive-map/. It is hosted on the MINERVA (Molecular Interaction NEtwoRks VisuAlization) platform [35–37] which interfaces with DrugBank [5], ChEMBL [6], CTDbase [7], and miRTarBase [8]. The majority of the proteins that have a 3D structure already resolved and available in the Protein Data Bank can be directly visualized and explored using MolArt [38], a built-in MINERVA platform visualization tool.

Plugins enable additional onsite analysis. In maps with defined pathway areas, the Gene set enrichment analysis (GSEA) plugin [37] retrieves active data overlays and performs enrichment analysis, highlighting pathways significantly enriched for data overlays. These data can be user-provided. Adverse drug reactions plugin [37] links an external data file to the corresponding map elements. Targets of drugs with identified adverse reactions are shown in the map and can be filtered. The Disease-variant associations plugin [37] indicates genes with variants associated with a given disease [37]. Map exploration plugin [37] enables focused molecular interaction network exploration (e.g., of the neighborhood of a molecule appearing multiple times in a network) [37]. Centrality plugin [39] calculates network topology values. Overlays plugin [39] automatically creates, displays, or removes multiple overlays from uploaded data files [39].

## Discussion

### Constructed models

The difference in the number of nodes and interactions between the original Manual HCM model in CellDesigner XML format and its uploaded version is caused by the incompatibility of the Cytoscape [40] and CellDesigner XML formats. The incompatibility is also evident from visual inspection of the network uploaded to Cytoscape/NDEx [41–43], where empty elements (reactions represented as nodes) constitute 53.95%. The inaccurate number of elements and misconstructed visual representation raised questions regarding the reliability of CellDesigner XML format in any Cytoscape analysis.

Visual inspection of networks revealed a weakness of the machine-curated models: the absence of compartments, which can be important for diseases like HCM, where a molecular signal is context-specific (organelle, cell, tissue, organ).

When the number of elements and interactions in models is taken as a criterion, the machine-curated models proved to be a richer source of information. Whether that abundance is noise or a broader view of the topic is yet to be determined.

The general problem of machine-curated models is the misleading labeling of the elements. Abbreviations like LV (a common abbreviation for the left ventricle in HCM articles) are turned into amino acid sequences (Leu-Val). Elements starting with Greek letters (e.g. α-adrenergic receptor) are turned into labels that consist of Greek letters only (e.g., α).

### Network analysis of the generated models

Comparing the original Manual HCM model in CellDesigner XML format and the same model (same elements and interactions) transcribed to the network table, we got different values for topological parameters in network analysis for all relevant measures. Taken together with the unsatisfactory result of upload for the model in CellDesigner XML format, we suggest that, although this format is readable by some Cytoscape tools, it should not be used for network analysis.

### Topological analysis

The average number of neighbors is the highest in the INDRA-assembled PubMed+PathwayCommons HCM model and the lowest in the Truncated INDRA DB HCM model. That is as expected because the INDRA-assembled PubMed+PathwayCommons HCM model is built using “neighborhood” query for the list of genes associated with HCM. “Neighborhood” query returns the neighborhood around a set of source genes [13], which is then incorporated in the model—it adds both elements and their neighbors to a model at the same time. The choice of the Truncated INDRA DB HCM model statements was based only on the correctness of a limited set of statements, so the discontinuity (manifested also as a lack of neighborhood connections) in the model was expected. All other models have a comparable average number of neighbors, with an element usually having two neighbors.

Network diameter indicates how distant the two most distant nodes are. It is a parameter of graph “compactness” (overall proximity between nodes) [44]. In order to compare the compactness of graphs of different sizes, we determined the network diameter per element. The Tabular manual HCM model was far more compact than the machine-curated models. At the same time, network diameter per element for the Manual HCM model had the lowest values, probably due to incompatible format.

Characteristic (average) path length represents “closeness” in a network [45]. It is defined as the average distance between all pairs of its nodes [46]. The characteristic path length is largest for the Tabular manual HCM model, closely followed by the INDRA DB HCM model, INDRA-assembled PubMed HCM model, INDRA-assembled PubMed+PathwayCommons HCM model, and Truncated INDRA DB HCM model. Characteristic (average) path length for the Manual HCM model has value 1, which is probably the result of incompatible CellDesigner XML format.

Clustering coefficient is a measure of local cohesiveness [47]. The clustering coefficient of a network is the average of all its individual clustering coefficients [48]. It is the largest for the Tabular manual HCM model. The Manual HCM model has a clustering coefficient of 0.0.

Network density is the number of existing relationships relative to a possible number. Dense networks are more important for control than for information. Dense networks tend to generate a lot of redundant information. Large networks tend to be sparse [49].

### Nodes’ centrality scores

There was no consensus between networks about the top elements in terms of centrality measures. This result is partially a consequence of diverse labeling between models, along with inconsistent labeling within models. Some rare elements were found as intersections of these sets, but they reflect the combination of the same principle for labeling, simultaneously with consistency about the highest values of centrality measures. Conclusions regarding the consensus turned out not to depend on the choice of centrality measure. The effect of different number of elements in networks on centrality measures and consequent comparison of top 10% of nodes is hard to predict and generalize, and could be the subject of a future research. Although this issue is partially and roughly resolved by using the same proportion of the elements (10%), the consensus between networks about the top elements in terms of centrality measures is affected by number of elements in networks, with impact and magnitude that are yet to be estimated.

### The most important nodes

Although the actually important nodes are estimated as important ones for all the models, the INDRA-assembled PubMed+PathwayCommons HCM model had the most less-expected elements estimated as being the most important ones.

For all models, among the group of elements estimated as the least important, most of the nodes are indeed less important for HCM. However, in the same group, there were some elements that are considered as important. We suggest that happens because of diverse labeling of closely related or same elements. K-shell decomposition algorithm assigns a weight based on the degree of a node (number of connections that it has to other nodes) and the adjacent nodes. Accordingly, diverse labeling makes these elements scattered, and thus less connected.

Venn diagrams for the most important nodes of all networks revealed that a consensus is achieved with respect to calcium, while other 95 percentile bucket elements were rarely the most important in a few models.

Venn diagrams for the least important nodes of all networks revealed that there is no consensus about the least important elements either, which is as expected because those elements represent noise or additional (non-essential) information.

In an interpretation context, wk-shell-decompositions and measures of centrality both tell us about importance of a node, but wk-shell-decompositions and each of centrality measures have different criteria of what is important and how is it estimated (i.e. calculated).

### Reliability of interactions

The PE-measure tool [50] demonstrated useful noise reduction in networks, especially in the INDRA DB model. We suggest that the combination of INDRA DB and PE-measure (or equivalent) tools could be beneficial for other disease models as well. The estimated best reliability threshold could also serve as a rough assessment of the level of noise in models. In this respect, the INDRA-assembled PubMed+PathwayCommons HCM model and INDRA DB model contain much more noise than the Tabular manual HCM model, INDRA-assembled PubMed HCM model, and especially the Truncated INDRA DB HCM model (which has the lowest estimated reliability threshold).

At the moment, there is no strict, straightforward, nor objective way to estimate where the border between the clutter and definite molecular elements involved in the disease is.

Disease modelers interested in domain knowledge consistency of models might be interested in what do combinations of the applied noise-removal technique and each of these model-generation techniques could bring, since model-generation techniques do not all generate same type of clutter.

### Cooperatively working elements

Most of the determined functional modules (cooperatively working elements) are possible and relevant for HCM (Additional file 3). All the machine-curated models contained ambiguous elements (due to imprecise labeling), except the Truncated INDRA DB, for which before construction such elements were excluded. All machine-curated models contained exogenous elements, except the Truncated INDRA DB. In the INDRA-assembled PubMed+PathwayCommons HCM model, functional modules containing exogenous elements dominated. Although these functional modules do not represent HCM itself properly, this approach could be interesting in cases where interactions between diseases and external factors are studied.

### Factors that affect the quality of machine-curated models

#### Reading systems’ performance

We propose assigning weights to statements extracted by a reading system that is favorable with regard to a particular use-case instead of giving preference to more numerous identical statements extracted. The choice of the reading system (and proposed weighting) is a trade-off between quantity and quality and could be guided by the molecular context and type of reactions important for a disease.

Although the RLIMS-P reading system demonstrated higher statement extraction performance, it is specifically designed to extract protein phosphorylation information. Favoritism of RLIMS-P due to its high extraction performance and, consequently, a large volume of phosphorylation statements should be revised for each disease of interest individually. Phosphorylation is the most common post-translational protein modification, and a key component of signal transduction [51]. However, statements about phosphorylation in HCM overshadowed other reaction types in the INDRA DB. Although we cannot pinpoint the exact contribution of phosphorylation to HCM mechanisms, especially in terms of understudied (“dark”) kinases [52], our suggestion is that phosphorylation statements should be dosed based on the model purpose. When models are built to enable hypothesis generation, abundance of phosphorylation statements is useful; when the purpose is to find key elements, they could produce an imbalance in the analysis.

#### Query constraints in machine-curated models

In HCM query by MeSH, the average year of publication is 10 years apart from the current research, which makes a difference in the overall representation of HCM, as more recent HCM research has brought in a whole additional quantum of knowledge. Moreover, query by MeSH returned a lot of animal studies, which are mostly aggregating noise in models for diseases like HCM, where animal models do not fully replicate human HCM [53]. For those reasons, we suggest that, for machine-curated models, the best approach to finding elements for HCM models is to query by keywords. Relying on MeSH, both fully or partially, should be avoided. HCM research tagged with MeSH is usually basic research, whereas HCM applied research is usually easier to find using keywords.

### Interactive HCM map

The interactive HCM Map is both human- and machine-readable and represents a platform for sharing and gathering molecular mechanisms of HCM and a standalone basis for in silico exploration. It also serves as a template for uploading and visualizing multiple datasets. It is the only publicly available knowledge resource dedicated to HCM.

### Related work

To the best of our knowledge, this is the first attempt to compare human and machine-curated disease models and examine how the choice of different query constraints in machine approaches can affect disease modeling.

Hoyt et al. (2019) manually evaluated 2989 statements generated by INDRA using REACH and Sparser readers containing studied genes from MEDLINE abstracts and PubMed Central full-text articles, following which 30.7% of statements were marked as correct, 48.6% required manual correction, and 20.7% could not be corrected. The criterion for correctness was that “all” aspects of the statement, including the subject and object entities, relationship type, phosphorylation, and other post-translational modifications, were extracted to the same extent as careful manual curation could. The authors identified errors in BEL statements extracted from INDRA. The most common error was wrong name entity recognition. Other common errors were the improper assignment of the subject and object, semantic incorrectness due to the presence of a negation word, and errors arising from evidence that did not actually include relations between the subject and object entities [11].

Allen et al. (2015) showed that the DRUM system (Deep Reader for Understanding Mechanisms, a version of the general-purpose TRIPS NLP system customized for extraction of molecular mechanisms from biomedical text) has performance (precision and recall) close to human experts in extracting the molecular mechanisms from text, and it was the best performing system among those evaluated. The same authors also found high precision among human biologists, but considerable non-overlap in the answers they provided. That accounted for the approximately 0.50 recall for either of the human teams they observed, using the pooled answers of the two teams as the gold standard [54].

Cohen et al. (2015) carried out a test with two expert human biologists and reading systems. Their task was to identify as many relationships as possible between six text passages and a prior model. Four kinds of relationships between texts and prior models were probed: the text might corroborate or contradict something in the model; it might introduce a new mechanism or a new relationship between entities in the model. Before the test began, biology experts on the evaluation team prepared a gold standard—a list of assertions. Recall was defined as the fraction of relationships that should have been found that were actually found, and precision as the fraction of the relationships found that were in the gold standard. The two expert human biologists’ recall scores were less than 0.5 (they failed to notice roughly half of the relationships between the texts and the prior models). However, their precision was very high: 0.86–1.00. They noticed different relationships, they disagreed with each other. They also noticed some relationships that the evaluation team had not. For the same task, the best recall score for a reading system was 0.4 with an associated precision score of 0.67. The least effective system achieved 0.03 recall at 0.33 precision. The authors assumed that human expertise probably includes an ability to not notice assertions that are “obvious” or “unimportant” [55].

Allen et al. (2018) studied how different extensions and customizations of the TRIPS parser affected performance [15]. Bose et al. (2020) used decisions from a statistical word sense disambiguation system SupWSD to advise the logical semantic parser TRIPS. Significant improvement across all metrics was found using this approach, with roughly 14% improvement to raw accuracy, although the research was not conducted on biomedical literature specifically [56].

While other authors have focused on reading systems’ performance as parsers (precision, recall, and F1 score—often defined differently), we focused on their potential to build models that would be equal to the models built by humans: containing reliable information (accuracy of extracted statements, based on human estimation) and providing complete information (extraction performance). We believe that the reliability of the information is the principal aspect of any reading system for biomedical knowledge curation.

Interactive disease maps have so far been generated for Alzheimer’s disease [57], cancer [58], Parkinson’s disease [59], influenza A virus replication cycle [60], rheumatoid arthritis [22], asthma [61, 62], inflammation [63], and others.

### Future directions

CellDesigner XML format should not be used for network analysis in Cytoscape. A higher level of interoperability between CellDesigner XML (and related) and INDRA generated formats and platforms would be useful because only in that case would direct comparison or better complementation of human- and machine-curated models be possible.

In machine-curated models, query constraints strongly affect the final disease models, so they should be chosen carefully and according to the purpose, with complete information about the advantages and disadvantages that each approach brings. Although we have shown that the PubMed database is a reliable source of information for human reading, the REACH reading system is equally or more accurate than other reading systems, and we suggest that a period of “last 10 years” is optimal for HCM research; the strategy that unites all these components derived a suboptimal (noisy and containing blurred key pathways) HCM model. More research is required, about the advantages and disadvantages of particular query constraints and their combinations for machine-curated models.

There is an urgent need for quality control criteria for disease models. Owing to the many techniques available for generating disease models, the formalization of minimal requirements for adequate quality of disease models or definition of methods for estimation of the quality of disease models are necessary. Such an approach could also accelerate and direct the development of more sophisticated techniques for building useful and representative disease models.

The Interactive HCM Map represents the body of knowledge available today, a summary of all major molecular pathways involved in HCM. Since some molecular mechanisms underlying HCM are still unknown, more interactions have yet to be identified. The HCM map will be constantly updated and improved, involving the community of HCM signaling experts.

### Limitations

Although our goal was a comprehensive comparison of models produced by different approaches (as a whole, by the most central and important elements, by the reliability of interactions and the level of noise they contain, as well as by cooperatively working elements), there is no single correct way to compare models and their quality. Moreover, since the molecular mechanisms underlying HCM are still only partially understood, we cannot claim that some interactions are more important or less possible—we can only assess the extent to which results are in line with the literature. Our analysis covered only the first phase of biomedical knowledge curation (and not the subsequent manual, semi-automatic, or automatic re-curation), so as to isolate only the effects of the selections made in this phase. Since we studied only one disease, we cannot generalize our findings to all diseases and models. In manual disease modeling, different persons cannot produce completely consistent results. Consequently, our results show the features of a single manual model made by a particular person rather than features of manual disease modeling itself. Currently there are no criteria for the diverse characteristics of different models.

### General

The rapid growth and accumulation of biomedical knowledge demands its structuring so that computers can assist in its interpretation [11] and comprehensive understanding. Disease models still need plenty of human input in the curation or re-curation phases, although semi-automatic or automatic re-curation options are emerging and can reduce time-consuming manual effort. Our results show how better performance can be attained even without the development of highly complex technologies. Selections made in the first phase of biomedical knowledge curation can affect overall performance. Our results show the effect of different strategies (techniques, query constraints, and reading systems) that should be considered in this phase. This evaluation also identified approaches that could be combined in order to achieve a specific goal of disease modeling. We anticipate that these results could be helpful for developers of the reading systems and model assemblers and may improve performance.

Manual curation represents the gold standard for information extraction in biomedical research [12] and is most suitable for models that will be used as a base for mathematical models generation, because only high-quality elements will be incorporated into the model. On the other side, manual curation is time- and effort-consuming. Automated curation is useful in situations where the more elements is the better, such for new hypothesis generation, because it provides more substance.

INDRA’s BioPAX API for the Pathway Commons database query is useful in automatic approach when paths between sets of genes are important and especially when microRNAs should be included in the model. INDRA’s PubMed literature client is favorable when focus is on available biomedical literature. INDRA Database is preferable when all available information is needed. All automated approaches generate a high level of noise. Although we expected the best results when the two approaches were combined: use of INDRA Database (expected to provide a high volume of information) with latter human intervention (expected to rigorously remove the clutter), in our case the model generated was too disconnected to be useful. In our case, the best automated approach for finding molecular mechanisms from clinical research was to query by keywords, while for finding elements from preclinical research query by MeSH was better. The PE-measure tool [50] demonstrated useful noise reduction in networks.

## Conclusions

There are many ways and resolutions for a disease to be modeled. Different approaches for the curation of models for the same disease can produce models with diverse characteristics and they give rise to utterly different conclusions in subsequent analysis. The final purpose of the model should direct the choice of techniques and tools for the curation. Manual curation represents the gold standard for information extraction in biomedical research and is most suitable when only high-quality elements for models are required. Automated curation provides more substance, but high level of noise is expected. Strategic combinations of query constraints, reading systems, and techniques like PE-measure could improve the performance and quality of machine-curated models. Different curation strategies can also reduce the level of human input.

## Methods

**Fig. 7:**
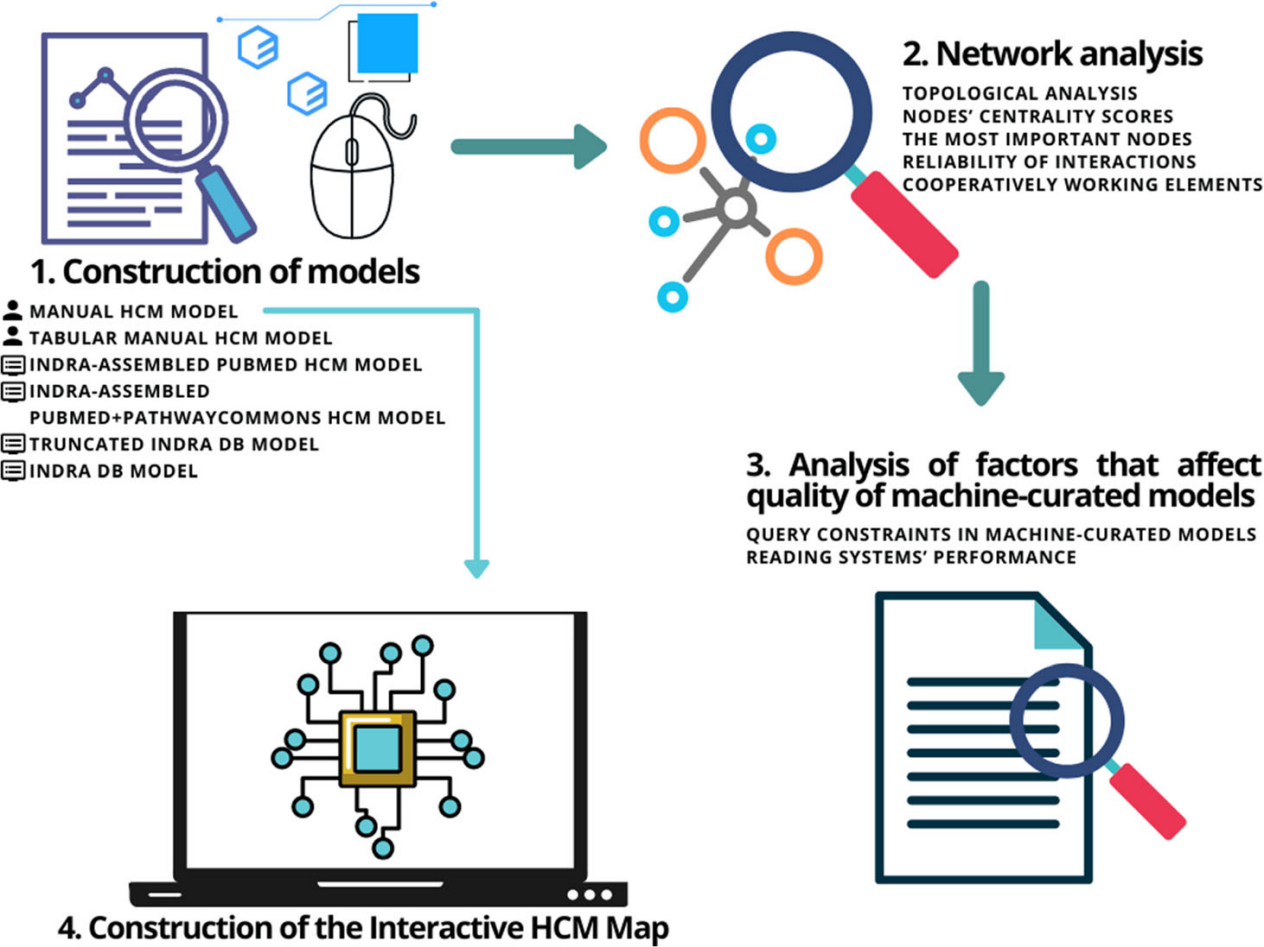
Phases of the research

Our research comprises four parts: construction of HCM models using different approaches, network analysis of the generated models, analysis of factors that affect the quality of machine-curated models, and construction of the Interactive HCM Map (Fig. 7).

### Construction of models

#### Manual HCM model

Construction of the Manual HCM model started with an extensive literature search in PubMed, for the molecular mechanisms underlying HCM. Relevant key phrases like “noncoding RNA hypertrophic cardiomyopathy,” “micro RNA hypertrophic cardiomyopathy,” “gene hypertrophic cardiomyopathy,” “signaling hypertrophic cardiomyopathy,” among others, and the filter “10 years” (for covering the period 2010–2020) were used for selection of the literature. First, well-established “consensus” information was retrieved from major reviews, and details from recent original publications were added subsequently.

The information was represented in Systems Biology Markup Language (SBML) format [64], as a Systems Biology Graphical Notation (SBGN) diagram [65] using CellDesigner v 4.4.2. Annotations for all the components (RNAs, genes, and proteins) were added using Minimal Information Requested In the Annotations of Models (MIRIAM) [66].

#### Tabular manual HCM model

All species and reactions from the original Manual HCM model XML file were manually transcribed to nodes and interactions of a network table in XLSX format.

#### INDRA-assembled PubMed HCM model

The model was assembled using INDRA [13]: INDRA’s PubMed literature client was used with the search term “hypertrophic cardiomyopathy” (major_topic = True) and filtering out results older than January 1, 2010. The content was read using the REACH reading system [14]. The statements extracted were grounded, mapped, and preassembled (de-duplicated and arranged in a hierarchy) before they were assembled using Cytoscape networks assembler for further analysis. Additional file 5 contains the code used for the generation of the model.

#### INDRA-assembled PubMed+PathwayCommons HCM model

The model was assembled using INDRA [13]: one collection of statements was generated from the Pathway Commons database [4] via INDRA’s BioPAX API, with “neighborhood” query, for a list of genes associated with HCM: *GAA*, *ACTC1*, *ACTN2*, *ANKRD1*, *CALR3*, *CASQ2*, *CAV3*, *CRYAB*, *CSRP3*, *DES*, *FHL1*, *FLNC*, *GLA*, *JPH2*, *LAMP2*, *LDB3*, *MYBPC3*, *MYH6*, *MYH7*, *MYL2*, *MYL3*, *MYLK2*, *MYOZ2*, *MYPN*, *NEXN*, *PLN*, *PRKAG2*, *TCAP*, *TNNC1*, *TNNI3*, *TNNT2*, *TPM1*, *TTR*, and *VCL*.

Another collection of statements for this model was compiled using INDRA’s PubMed literature client with the search term “hypertrophic cardiomyopathy” (major_topic = True) and filtering out results older than January 1, 2010. The content was read using the REACH reading system [14]. All the statements retrieved from both collections were gathered, and then grounded, mapped, and preassembled (de-duplicated and arranged in a hierarchy) before they were assembled using Cytoscape networks assembler for further analysis. Additional file 6 contains the code used for the generation of the model.

#### Truncated INDRA DB model

Statements were found using INDRA Database with the MeSH query constraint “Cardiomyopathy, Hypertrophic, Familial.” Only statements that were completely correctly extracted from the text were incorporated into the Truncated INDRA DB model. The criteria for correctness were that all aspects of the statement, including subject and object, their labels, interaction type, and interaction direction, were extracted the same way as careful manual curation would. The statements were manually transcribed to nodes and interactions in a network table in XLSX format.

#### INDRA DB model

Statements were found using the INDRA Database with the MeSH query constraint “Cardiomyopathy, Hypertrophic, Familial.” All statements were incorporated into the INDRA DB model. The statements were manually transcribed to nodes and interactions in a network table in XLSX format.

### Network analysis of the generated models

Network analysis was conducted analogous to network analysis in our previous research [67].

All models were imported to Cytoscape v. 3.8.2 [40] for further analysis and uploaded to NDEx v. 2.5.0 [41–43].

#### Topological analysis

Topological analysis of each network was performed using Network Analyzer v. 4.4.6 [68], a built-in Cytoscape tool. All networks were analyzed as directed graphs.

Definitions of the topological measures and other parameters were as following. “The neighborhood of a given node is the set of its neighbors. The connectivity of a given node is the size of its neighborhood. The average number of neighbors indicates the average connectivity of a node in the network. A normalized version of this parameter is the network density. The density is a value between 0 and 1. It shows how densely the network is populated with edges. The length of a path is the number of edges forming it. The eccentricity is the maximum non-infinite length of a shortest path between a given node and another node in the network. The network diameter is the largest distance between two nodes. If a network is disconnected, its diameter is the maximum of all diameters of its connected components. The diameter can also be described as the maximum node eccentricity. The network radius is the minimum among the non-zero eccentricites of the nodes in the network. The average shortest path length, also known as the characteristic path length, gives the expected distance between two connected nodes.” [69].

“In directed networks, the clustering coefficient *C*_*n*_ of a node *n* is defined as: *C*_*n*_ = *e*_*n*_/(*k*_*n*_ (*k*_*n*_-1)), where *k*_*n*_ is the number of neighbors of *n* and *e*_*n*_ is the number of connected pairs between all neighbors of *n*. The clustering coefficient of a node is always a number between 0 and 1. The network clustering coefficient is the average of the clustering coefficients for all nodes in the network.” [69].

“Two nodes are connected if there is a path of edges between them. Within a network, all nodes that are pairwise connected form a connected component. The number of connected components indicates the connectivity of a network – a lower number of connected components suggests a stronger connectivity. The number of multi-edge node pairs indicates how often neighboring nodes are linked by more than one edge.” [69].

Since the diameter of a graph is better defined when compared to the total number of nodes in the graph [39], we also determined the network diameter per element.

#### Nodes’ centrality scores

Betweenness, bottleneck, closeness, clustering coefficient, degree, DMNC, eccentricity, EPC, MCC, MNC, radiality, and stress centrality measures were used. Centrality scores for each node of each network were calculated and the top 10% elements for each of the centrality measures of each network were visualized using the Cytoscape CytoHubba app v. 0.1 [70] and uploaded to NDEx. Venn diagrams for the top 10% elements for each centrality measure of each network were drawn using the Venn diagram tool [71].

#### The most important nodes

Estimation of the most import nodes in networks and their partition into shells based on that rank was performed by wk-shell-decomposition using the Cytoscape Wk-shell-decomposition app v. 1.1.0 [33]. Each network was represented as a packed concentric ring sorted by k-shell and gradient of nodes’ color applied based on k-shell. Rank and k-shell were calculated for each node of each network. Venn diagrams for the most and least important nodes of all networks were drawn using the Venn diagram tool [71].

#### Reliability of interactions

Models with a reduced level of noise were generated using the Cytoscape PE-measure app v. 1.0 [50] and uploaded to NDEx. The best reliability threshold for each model was estimated by a human domain expert, following the principle of finding the network that covers HCM mechanisms the best with the least clutter. The term clutter in this case covered: wrong elements, wrongly labeled elements, and all the elements that should not be present in an ideal disease model. A human domain expert was inspecting the networks with different thresholds applied and chose the level which produced the network that best represent the disease (according to up-to-date scientific literature, with the most of the known elements involved in the disease and the least of the clutter).

#### Cooperatively working elements

The cooperatively working elements (functional modules) of each network were detected by near-clique mining using the Cytoscape NCMine app v. 1.3.0 [34]. All models were analyzed as directed networks.

### Factors that affect the quality of machine-curated models

#### Query constraints in machine-curated models

PubMed searches by “Cardiomyopathy, Hypertrophic, Familial” MeSH Term with filter “in the last 10 years,” as well as “Cardiomyopathy, Hypertrophic, Familial” MeSH Major Topic with filter “in the last 10 years” were conducted manually and compared with PubMed search for keywords “familial hypertrophic cardiomyopathy,” “hypertrophic cardiomyopathy,” exact match keywords “familial hypertrophic cardiomyopathy,” and” hypertrophic cardiomyopathy,” all with filter “10 years.”

A deeper analysis of all papers listed in the INDRA Database tagged with the MeSH was carried out manually; the average year of publication, along with the percentage of species studied, was calculated.

#### Reading systems’ performance

We compared the extraction performance of all reading systems used in the INDRA Database (ISI/AMR, RLIMS-P, Eidos, TRIPS/DRUM, Sparser, REACH), by calculating their contribution to each individual statement and the database query by MeSH for HCM as a whole. We classified all statements extracted from the query into 28 reaction types and calculated the corresponding contribution of each reading system.

We compared the accuracy of reading systems capable of translating the most important types of reactions (including subject, interaction, and object) for HCM: Sparser, REACH, and TRIPS. The output of Sparser, REACH, and TRIPS reading systems, for all text segments for which Sparser extracted a statement, was analyzed by the same human curator. We have proposed an issue for each of the statements that were assessed as incorrectly extracted and estimated the contribution of each issue to the inaccuracies of the reading systems.

We evaluated the adequacy of the Eidos reading system for studying a disease through human estimation of the meaningfulness of extracted statements.

### Construction of interactive HCM map

The Manual HCM model was transformed into an HCM knowledge resource and made publicly available using the MINERVA (Molecular Interaction NEtwoRks VisuAlization) platform v. 15.1.2 [35–37]. Disease-variant associations v. 1.0.0 [37], Adverse drug reactions v. 1.0.0 [37], Map exploration v. 1.0.0 [37], Gene Set Enrichment Analysis (GSEA) v. 0.9.1 [37], Centrality v. 0.9.0 [39], and Overlays v. 0.9.0 [39] plugins were added.

## Supplementary Information

**Additional file 1.** Networks represented as packed concentric ring sorted by k-shell. Each network represented as a packed concentric ring sorted by k-shell and gradient of nodes’ color applied based on k-shell.

**Additional file 2.** Ranks and k-shells for each node of each network.

**Additional file 3.** Cooperatively working elements. Cooperatively working elements (functional modules) detected and their likely implications.

**Additional file 4.** Eidos statements. Statements extracted for the INDRA DB model by Eidos reading system.

**Additional file 5.** Code used for generation of INDRA-assembled PubMed HCM model.

**Additional file 6.** Code used for generation of INDRA-assembled PubMed+PathwayCommons HCM model.

**Additional file 7.** Original Manual HCM model.

## Data Availability

The datasets generated and/or analyzed during the current study are included in this published article and its additional information files. Some intermediate results of analysis are not publicly available due to their volume but are available from the corresponding author on reasonable request. The Interactive HCM map is available at https://silicofcm.eu/interactive-map/.
