# Supplementary material for: Humans and machines in biomedical knowledge curation: hypertrophic cardiomyopathy molecular mechanisms’ representation
Source: BioData Min. 2021 Oct 2;14:45. doi: 10.1186/s13040-021-00279-2 (PMC8487578; doi:10.1186/s13040-021-00279-2)
Supplement: Supplementary file 1 — Additional file 1. Networks represented as packed concentric ring sorted by k-shell. Each network represented as a packed concentric ring sorted by k-shell and gradient of nodes’ color applied based on k-shell. [file 13040_2021_279_MOESM1_ESM.docx]

**Additional file 1. Networks represented as packed concentric ring sorted by k-shell**

Elements closer to center and colored redder are estimated as more important.

**Tabular manual HCM model**

**
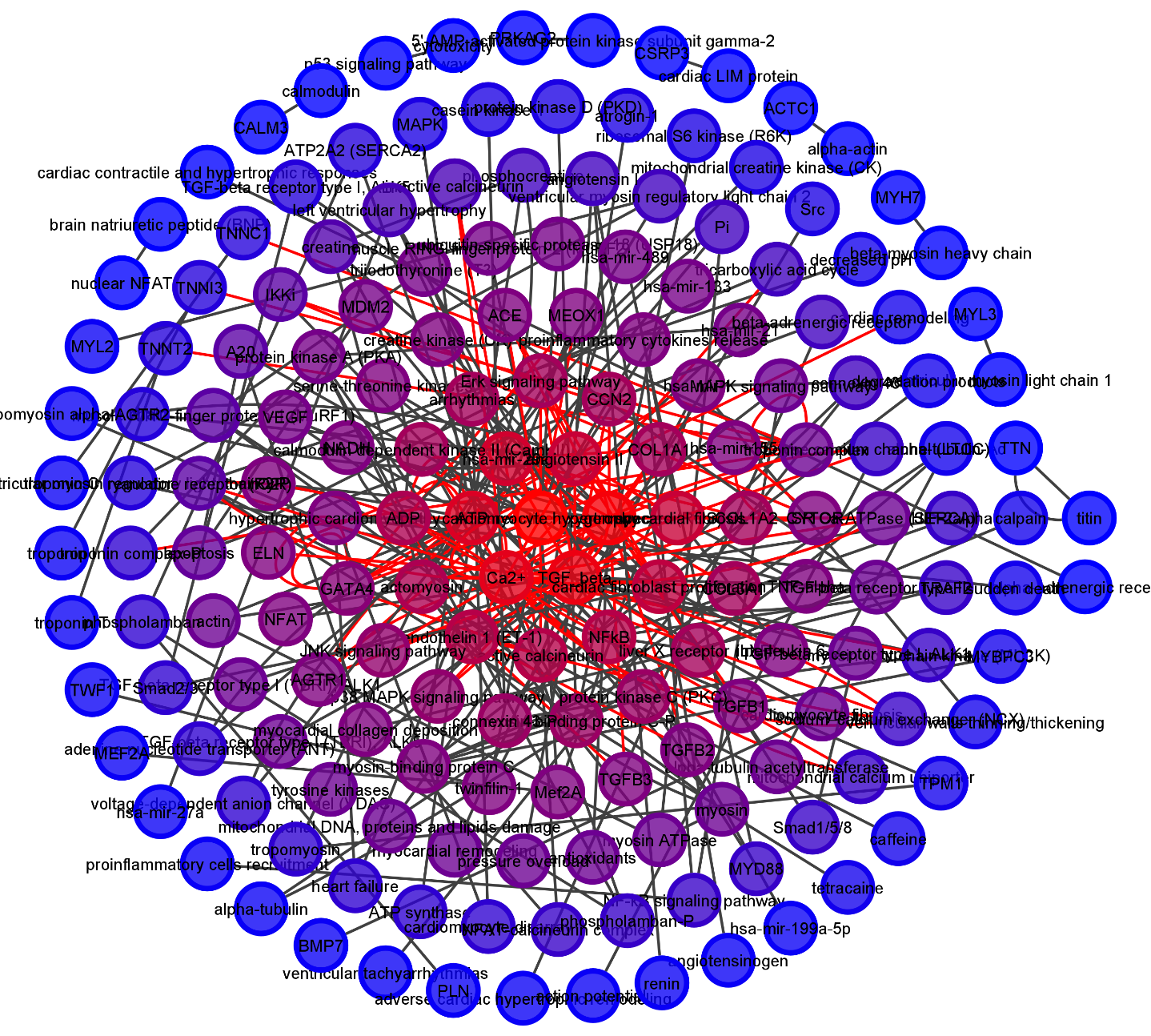
**

**INDRA-assembled PubMed HCM model**

**
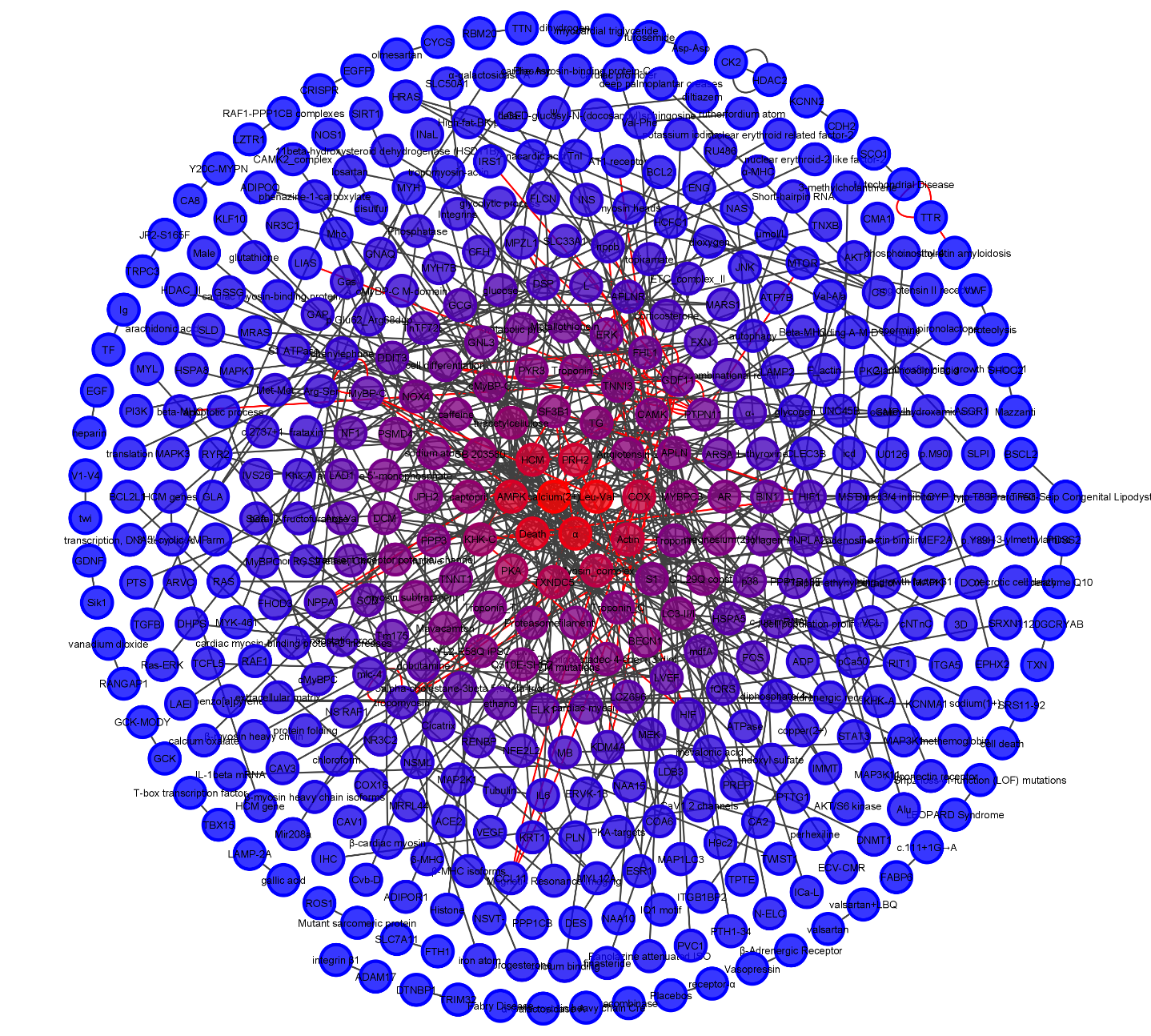
**

**INDRA-assembled PubMed+PathwayCommons HCM model**

**
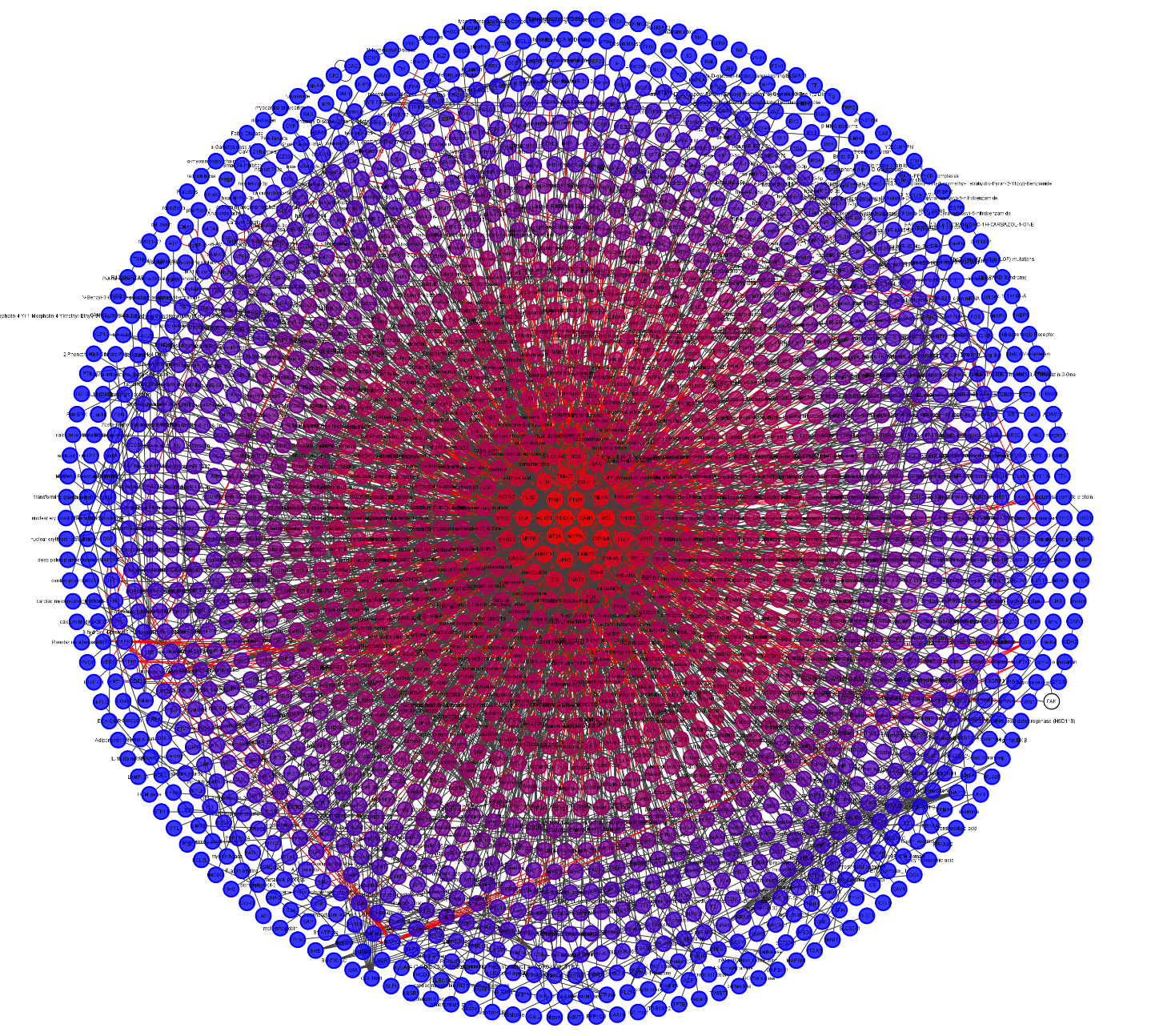
**

**Truncated INDRA DB HCM model**

**
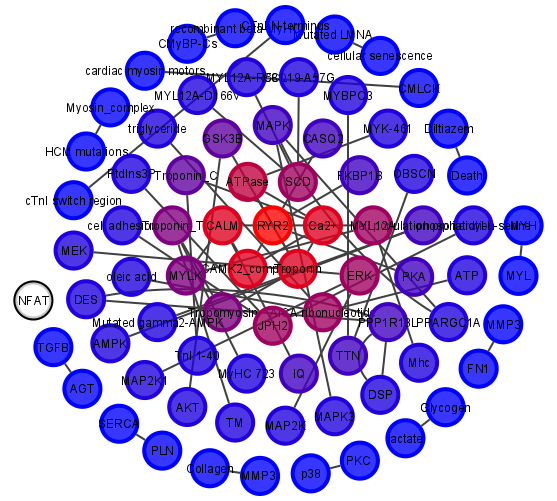
**

**INDRA DB model**

**
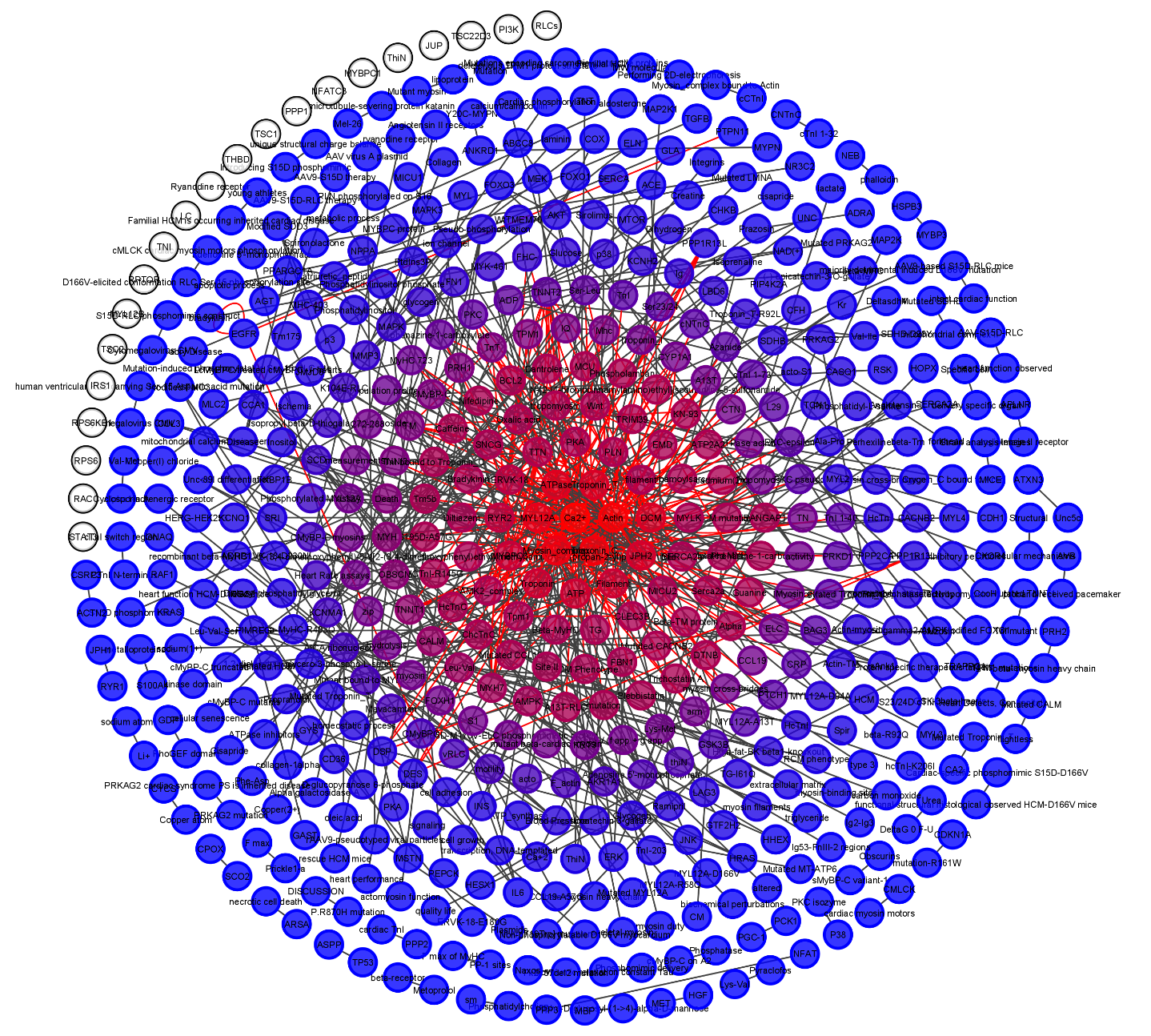
**
