# Supplementary material for: Humans and machines in biomedical knowledge curation: hypertrophic cardiomyopathy molecular mechanisms’ representation
Source: BioData Min. 2021 Oct 2;14:45. doi: 10.1186/s13040-021-00279-2 (PMC8487578; doi:10.1186/s13040-021-00279-2)
Supplement: Supplementary file 2 — Additional file 2. Ranks and k-shells for each node of each network. [file 13040_2021_279_MOESM2_ESM.docx]

**Additional file 2. Ranks and k-shells for each node of each network**

**Tabular manual HCM model**

| name | _wks_percentile_bucket | _wkshell |
| --- | --- | --- |
| cardiomyocyte hypertrophy | 95 | 29 |
| reactive oxygen species (ROS) | 95 | 28 |
| TGF_beta | 95 | 27 |
| Ca2+ | 95 | 26 |
| ATP | 95 | 25 |
| hsa-mir-29a | 95 | 24 |
| myocardial fibrosis | 95 | 23 |
| angiotensin II | 95 | 23 |
| cardiac fibroblast proliferation | 90 | 22 |
| endothelin 1 (ET-1) | 90 | 21 |
| active calcineurin | 90 | 21 |
| NFkB | 90 | 21 |
| ADP | 90 | 20 |
| actomyosin | 90 | 20 |
| liver X receptor (LXR) | 85 | 19 |
| COL3A1 | 85 | 19 |
| COL1A2 | 85 | 19 |
| COL1A1 | 85 | 19 |
| CCN2 | 85 | 19 |
| Erk signaling pathway | 90 | 19 |
| arrhythmias | 90 | 19 |
| calmodulin dependent kinase II (CamKII) | 90 | 19 |
| connexin 43-P | 85 | 18 |
| myosin-binding protein C-P | 85 | 18 |
| protein kinase C (PKC) | 85 | 18 |
| p38 MAPK signaling pathway | 85 | 17 |
| JNK signaling pathway | 80 | 17 |
| GATA4 | 80 | 16 |
| O2 | 70 | 15 |
| proinflammatory cytokines release | 80 | 15 |
| ELN | 70 | 15 |
| TGFB3 | 75 | 15 |
| TGFB2 | 75 | 15 |
| TGFB1 | 75 | 15 |
| MEOX1 | 80 | 15 |
| interleukin 6 | 75 | 15 |
| TNF-alpha | 75 | 15 |
| CYTOR | 75 | 15 |
| hsa-mir-155 | 75 | 15 |
| NFAT | 70 | 15 |
| ACE | 80 | 15 |
| creatine kinase (CK) | 80 | 15 |
| serine-threonine kinases | 80 | 15 |
| NADH | 80 | 15 |
| AGTR1 | 70 | 15 |
| hsa-mir-1 | 75 | 15 |
| myocardial collagen deposition | 70 | 15 |
| myosin-binding protein C | 70 | 15 |
| hypertrophic cardiomyopathy | 80 | 15 |
| twinfilin-1 | 70 | 15 |
| Mef2A | 70 | 15 |
| hsa-mir-21 | 65 | 14 |
| hsa-mir-133 | 65 | 14 |
| VEGF | 70 | 14 |
| hsa-mir-489 | 65 | 14 |
| ubiquitin-specific protease 18 (USP18) | 65 | 14 |
| muscle RING-finger protein-2 (MuRF2) | 65 | 14 |
| triiodothyronine (T3) | 65 | 14 |
| MDM2 | 65 | 14 |
| protein kinase A (PKA) | 65 | 14 |
| antioxidants | 60 | 13 |
| myosin ATPase | 60 | 13 |
| myosin | 60 | 13 |
| actin | 55 | 13 |
| TGF-beta receptor type I (TBRI), ALK1 | 55 | 13 |
| TGF-beta receptor type I (TBRI), ALK5 | 55 | 13 |
| alpha-tubulin acetyltransferase | 60 | 13 |
| cardiomyocyte fibrosis | 60 | 13 |
| TGF-beta receptor type I, ALK1 | 60 | 13 |
| TGF-beta receptor type II | 60 | 13 |
| MAPK signaling pathway | 65 | 13 |
| tyrosine kinases | 55 | 13 |
| mitochondrial DNA, proteins and lipids damage | 55 | 13 |
| SR Ca-ATPase (SERCA) | 60 | 13 |
| troponin complex | 60 | 13 |
| myocardial remodeling | 55 | 13 |
| pressure overload | 55 | 13 |
| apoptosis | 55 | 12 |
| IKKi | 50 | 11 |
| A20 | 50 | 11 |
| muscle RING-finger protein-1 (MuRF1) | 50 | 11 |
| ryanodine receptor (RyR) | 50 | 11 |
| creatine | 50 | 10 |
| inactive calcineurin | 50 | 9 |
| left ventricular hypertrophy | 50 | 9 |
| angiotensin I | 50 | 8 |
| phosphocreatine | 50 | 8 |
| connexin 43 | 45 | 8 |
| beta-adrenergic receptor | 45 | 8 |
| tricarboxylic acid cycle | 45 | 8 |
| Pi | 45 | 8 |
| ventricular myosin regulatory light chain 2 | 45 | 8 |
| NFAT-calcineurin complex | 40 | 7 |
| myosin light chain kinase (MLCK) | 45 | 7 |
| phospholamban-P | 40 | 7 |
| NF-kB signaling pathway | 40 | 7 |
| MYD88 | 40 | 7 |
| TRAF2 | 45 | 7 |
| HIF-2alpha | 45 | 7 |
| Smad1/5/8 | 40 | 7 |
| mitochondrial calcium uniporter | 40 | 7 |
| sodiumâ€“calcium exchanger (NCX) | 40 | 7 |
| L-type calcium channel (LTCC) | 45 | 7 |
| ATP synthase | 40 | 6 |
| troponin complex-P | 35 | 6 |
| phospholamban | 35 | 6 |
| Smad2/3 | 35 | 6 |
| adenine nucleotide transporter (ANT) | 35 | 6 |
| voltage-dependent anion channel (VDAC) | 35 | 6 |
| tropomyosin | 35 | 6 |
| heart failure | 35 | 6 |
| cardiomyocyte disarray | 40 | 6 |
| ventricular myosin regulatory light chain 2-P | 35 | 5 |
| ATP2A2 (SERCA2) | 30 | 5 |
| TGF-beta receptor type I, ALK5 | 30 | 5 |
| TNNC1 | 30 | 5 |
| TNNI3 | 30 | 5 |
| TNNT2 | 30 | 5 |
| AGTR2 | 30 | 5 |
| protein kinase D (PKD) | 30 | 4 |
| casein kinase 1 | 30 | 4 |
| MAPK | 30 | 4 |
| Src | 25 | 4 |
| mitochondrial creatine kinase (CK) | 25 | 4 |
| ribosomal S6 kinase (R6K) | 25 | 4 |
| atrogin-1 | 25 | 4 |
| calpain | 25 | 3 |
| alpha-tubulin-Ac | 25 | 3 |
| degradation products | 25 | 3 |
| cardiac remodeling | 25 | 3 |
| decreased pH | 25 | 3 |
| tetracaine | 20 | 3 |
| caffeine | 20 | 3 |
| TPM1 | 20 | 3 |
| ventricular walls thinning/thickening | 20 | 3 |
| MYBPC3 | 20 | 3 |
| sudden death | 20 | 3 |
| renin | 20 | 2 |
| angiotensinogen | 20 | 2 |
| hsa-mir-199a-5p | 20 | 2 |
| hsa-mir-27a | 15 | 2 |
| proinflammatory cells recruitment | 15 | 2 |
| alpha-tubulin | 15 | 2 |
| BMP7 | 15 | 2 |
| ventricular tachyarrhythmias | 15 | 2 |
| PLN | 15 | 2 |
| adverse cardiac hypertrophic remodeling | 15 | 2 |
| action potential | 15 | 2 |
| MYL2 | 10 | 2 |
| tropomyosin alpha-1 chain | 10 | 2 |
| troponin C | 10 | 2 |
| troponin I | 10 | 2 |
| troponin T | 10 | 2 |
| TWF1 | 10 | 2 |
| MEF2A | 10 | 2 |
| brain natriuretic peptide (BNP) | 10 | 1 |
| nuclear NFAT | 10 | 1 |
| cardiac LIM protein | 5 | 1 |
| CSRP3 | 5 | 1 |
| 5'-AMP-activated protein kinase subunit gamma-2 | 5 | 1 |
| PRKAG2 | 5 | 1 |
| cytotoxicity | 5 | 1 |
| p53 signaling pathway | 5 | 1 |
| calmodulin | 5 | 1 |
| CALM3 | 5 | 1 |
| cardiac contractile and hypertrophic responses | 5 | 1 |
| alpha-adrenergic receptor | 0 | 1 |
| titin | 0 | 1 |
| TTN | 0 | 1 |
| ventricular myosin light chain 1 | 0 | 1 |
| MYL3 | 0 | 1 |
| beta-myosin heavy chain | 0 | 1 |
| MYH7 | 0 | 1 |
| alpha-actin | 0 | 1 |
| ACTC1 | 0 | 1 |

**INDRA-assembled PubMed HCM model**

| name | _wks_percentile_bucket | _wkshell |
| --- | --- | --- |
| calcium(2+) | 95 | 27 |
| Leu-Val | 95 | 26 |
| α | 95 | 25 |
| Death | 95 | 24 |
| AMPK | 95 | 23 |
| PRH2 | 95 | 22 |
| HCM | 95 | 22 |
| Actin | 95 | 21 |
| COX | 95 | 21 |
| Myosin_complex | 95 | 20 |
| TXNDC5 | 95 | 19 |
| PKA | 95 | 18 |
| KHK-C | 95 | 17 |
| triacetylcellulose | 95 | 16 |
| SB 203580 | 95 | 16 |
| captopril | 95 | 16 |
| MYBPC3 | 95 | 15 |
| Angiotensin-2 | 95 | 15 |
| TG | 95 | 15 |
| SF3B1 | 95 | 15 |
| Troponin | 95 | 14 |
| 2-aminooctadec-4-ene-1,3-diol | 90 | 14 |
| BECN1 | 90 | 14 |
| LC3-II/I | 90 | 14 |
| TnC-L29Q construct | 90 | 14 |
| magnesium(2+) | 90 | 14 |
| AR | 90 | 14 |
| APLN | 90 | 14 |
| CAMK | 90 | 14 |
| TNNI3 | 90 | 14 |
| Troponin_I | 90 | 14 |
| PYR3 | 90 | 14 |
| cMyBP-C | 90 | 14 |
| caffeine | 90 | 14 |
| sodium atom | 90 | 14 |
| JPH2 | 90 | 14 |
| PPP3 | 90 | 14 |
| TNNT1 | 90 | 14 |
| Troponin_T | 90 | 14 |
| Proteasome | 90 | 14 |
| filament | 90 | 14 |
| Troponin_C | 90 | 14 |
| S1 | 90 | 14 |
| transient receptor potential channel | 85 | 14 |
| myosin subfragment 1 | 85 | 14 |
| Mavacamten | 85 | 14 |
| MYL2-R58Q iPSC-CMs | 85 | 14 |
| Q510E-SHP2 | 85 | 14 |
| HCM mutations | 85 | 14 |
| NOX4 | 85 | 13 |
| PSMD4 | 85 | 13 |
| adenosine 5'-monophosphate | 85 | 13 |
| DCM | 85 | 13 |
| PTPN11 | 85 | 12 |
| GDF11 | 85 | 12 |
| FHL1 | 85 | 12 |
| ERK | 85 | 12 |
| Metallothionein | 85 | 12 |
| metabolic process | 85 | 12 |
| GNL3 | 85 | 12 |
| cell differentiation | 85 | 12 |
| p38 | 85 | 11 |
| Collagen | 85 | 11 |
| BIN1 | 85 | 11 |
| ARSA | 85 | 11 |
| DDIT3 | 80 | 10 |
| MyBP-C | 80 | 10 |
| NF1 | 80 | 10 |
| LAD1 | 80 | 10 |
| Arg-Val | 80 | 10 |
| Pulmonary Disease, Chronic Obstructive | 80 | 10 |
| SCD | 80 | 10 |
| Tm175 | 80 | 10 |
| dobutamine | 80 | 10 |
| 5alpha-cholestane-3beta,5,6beta-triol | 80 | 10 |
| ethanol | 80 | 10 |
| ELK1 | 80 | 10 |
| cardiac myosin | 80 | 10 |
| LCZ696 | 80 | 10 |
| LVEF | 80 | 10 |
| mdfA | 80 | 10 |
| HSPA5 | 80 | 10 |
| glucose | 80 | 9 |
| GCG | 80 | 9 |
| TnTF72L | 80 | 9 |
| L | 80 | 8 |
| DSP | 80 | 8 |
| FOS | 75 | 8 |
| c-jun mRNA | 75 | 8 |
| PPP1R13L | 75 | 8 |
| PNPLA2 | 75 | 8 |
| HIF1 | 75 | 8 |
| L-thyroxine | 75 | 8 |
| Î±- | 75 | 8 |
| recombinational repair | 75 | 8 |
| FXN | 75 | 8 |
| corticosterone | 75 | 8 |
| APLNR | 75 | 8 |
| mlc-4 | 75 | 7 |
| tropomyosin | 75 | 7 |
| Cicatrix | 75 | 7 |
| RENBP | 75 | 7 |
| NFE2L2 | 75 | 7 |
| MB | 75 | 7 |
| KDM4A | 75 | 7 |
| MEK | 75 | 7 |
| HIF | 75 | 7 |
| fQRS | 75 | 7 |
| NPPA | 70 | 7 |
| homeostatic process | 70 | 7 |
| MARS1 | 70 | 6 |
| ETC_complex_II | 70 | 6 |
| topiramate | 70 | 6 |
| nppb | 70 | 6 |
| SLC33A1 | 70 | 6 |
| MPZL1 | 70 | 6 |
| CFH | 70 | 6 |
| MYH7B | 70 | 6 |
| cMyBP-C M-domain | 70 | 6 |
| p.Glu62_Arg68dup | 70 | 6 |
| phenylephrine | 70 | 6 |
| Arg-Ser | 70 | 6 |
| frataxin | 70 | 6 |
| Khk-A | 70 | 6 |
| beta-D-fructofuranose | 70 | 6 |
| RGS2 | 70 | 6 |
| CLEC3B | 70 | 5 |
| glycogen | 70 | 5 |
| LAMP2 | 70 | 5 |
| autophagy | 70 | 5 |
| SCA | 65 | 5 |
| MyBPC | 65 | 5 |
| FHOD3 | 65 | 5 |
| cardiac myosin-binding protein-C increases | 65 | 5 |
| cMyBPC | 65 | 5 |
| NS RAF1 | 65 | 5 |
| NR3C2 | 65 | 5 |
| NSML | 65 | 5 |
| MAP2K1 | 65 | 5 |
| Tubulin | 65 | 5 |
| IL6 | 65 | 5 |
| ERVK-18 | 65 | 5 |
| NAA15 | 65 | 5 |
| LDB3 | 65 | 5 |
| mevalonic acid | 65 | 5 |
| ATPase | 65 | 5 |
| diphosphate(4-) | 65 | 5 |
| ADP | 65 | 5 |
| cell population proliferation | 65 | 5 |
| 17alpha-ethynylestradiol | 65 | 5 |
| adenosine | 65 | 5 |
| MSTN | 65 | 5 |
| INS | 60 | 5 |
| FLCN | 60 | 5 |
| glycolytic process | 60 | 5 |
| Integrins | 60 | 5 |
| Phosphatase | 60 | 5 |
| GNAQ | 60 | 5 |
| Gas | 60 | 5 |
| GAP | 60 | 5 |
| S1 ATPase | 60 | 5 |
| Met-Met | 60 | 5 |
| c.2737+1 | 60 | 5 |
| IVS26 | 60 | 5 |
| Smad3/4 inhibitor | 60 | 4 |
| icd | 60 | 4 |
| UNC45B | 60 | 4 |
| F_actin | 60 | 4 |
| Beta-MHC | 60 | 4 |
| ATP7B | 60 | 4 |
| JNK | 60 | 4 |
| dioxygen | 60 | 4 |
| HCFC1 | 60 | 4 |
| myosin heads | 60 | 4 |
| extracellular matrix | 55 | 4 |
| protein folding | 55 | 4 |
| chloroform | 55 | 4 |
| COX16 | 55 | 4 |
| MRPL44 | 55 | 4 |
| ACE2 | 55 | 4 |
| VEGF | 55 | 4 |
| KRT1 | 55 | 4 |
| PLN | 55 | 4 |
| PKA-targets | 55 | 4 |
| COA6 | 55 | 4 |
| CaV1.2 channels | 55 | 4 |
| PREP | 55 | 4 |
| indoxyl sulfate | 55 | 4 |
| copper(2+) | 55 | 4 |
| Î˛1-adrenergic receptor | 55 | 4 |
| pCa50 | 55 | 4 |
| VCL | 55 | 4 |
| Transforming growth factor Î˛1 | 55 | 4 |
| F-actin binding | 55 | 4 |
| RAF1 | 55 | 3 |
| ÎĽmol/L | 50 | 3 |
| NAS | 50 | 3 |
| ENG | 50 | 3 |
| BCL2 | 50 | 3 |
| AT1 receptor | 50 | 3 |
| TnI | 50 | 3 |
| Anacardic acid | 50 | 3 |
| IRS1 | 50 | 3 |
| tropomyosin-actin | 50 | 3 |
| MYH | 50 | 3 |
| disulfur | 50 | 3 |
| Mhc | 50 | 3 |
| LIAS | 50 | 3 |
| cardiac myosin-binding protein C. | 50 | 3 |
| MRAS | 50 | 3 |
| MAPK7 | 50 | 3 |
| apoptotic process | 50 | 3 |
| RYR2 | 50 | 3 |
| GLA | 50 | 3 |
| arm | 50 | 3 |
| RAS | 50 | 3 |
| MYK-461 | 50 | 3 |
| CCL11 | 45 | 3 |
| Magnetic Resonance Imaging | 45 | 3 |
| MYL12A | 45 | 3 |
| ESR1 | 45 | 3 |
| MAP1LC3 | 45 | 3 |
| H9c2 | 45 | 3 |
| CA2 | 45 | 3 |
| PTTG1 | 45 | 3 |
| IMMT | 45 | 3 |
| STAT3 | 45 | 3 |
| KHK-A | 45 | 3 |
| RIT1 | 45 | 3 |
| cNTnC | 45 | 3 |
| MAPK | 45 | 3 |
| MEF2A | 45 | 3 |
| CYP | 45 | 3 |
| U0126 | 45 | 3 |
| cGMP | 45 | 3 |
| PKGI | 45 | 3 |
| force-holding AÂ·MÂ·D complex | 45 | 3 |
| Val-Ala | 45 | 3 |
| MTOR | 45 | 3 |
| losartan | 40 | 3 |
| phenazine-1-carboxylate | 40 | 3 |
| NR3C1 | 40 | 3 |
| glutathione | 40 | 3 |
| GSSG | 40 | 3 |
| SLD | 40 | 3 |
| HSPA8 | 40 | 3 |
| beta-MHC | 40 | 3 |
| MAPK3 | 40 | 3 |
| HCM genes | 40 | 3 |
| 3',5'-cyclic AMP | 40 | 3 |
| ARVC | 40 | 3 |
| DHPS | 40 | 3 |
| TCFL5 | 40 | 3 |
| benzo[a]pyrene | 40 | 3 |
| Î˛-myosin heavy chain | 40 | 3 |
| CAV3 | 40 | 3 |
| Î˛-myosin heavy chain isoforms | 40 | 3 |
| CAV1 | 40 | 3 |
| Î˛-cardiac myosin | 40 | 3 |
| Î˛-MHC | 40 | 3 |
| Î˛-MHC isoforms | 40 | 3 |
| DOX | 35 | 3 |
| p.Y89H | 35 | 3 |
| p.T83P | 35 | 3 |
| p.M90I | 35 | 3 |
| salicylhydroxamic acid | 35 | 3 |
| 2-aminoadipic acid | 35 | 3 |
| spermine | 35 | 3 |
| CS | 35 | 3 |
| AKT | 35 | 3 |
| TNXB | 35 | 3 |
| Short-hairpin RNA | 35 | 3 |
| Î±-MHC | 35 | 3 |
| RU486 | 35 | 3 |
| potassium iodide | 35 | 3 |
| Val-Phe | 35 | 3 |
| beta-D-glucosyl-N-(docosanoyl)sphingosine | 35 | 3 |
| Î¨ | 35 | 3 |
| LGE | 35 | 3 |
| High-fat-BK Î˛ | 35 | 3 |
| INaL | 35 | 3 |
| 11beta-hydroxysteroid dehydrogenase (HSD11B) | 35 | 3 |
| 3D | 30 | 3 |
| IL-1beta mRNA | 30 | 2 |
| HCM gene | 30 | 2 |
| Mir208a | 30 | 2 |
| IHC | 30 | 2 |
| Cvb-D | 30 | 2 |
| ADIPOR1 | 30 | 2 |
| Histone | 30 | 2 |
| NSVT- | 30 | 2 |
| PPP1CB | 30 | 2 |
| DES | 30 | 2 |
| NAA10 | 30 | 2 |
| IQ1 motif | 30 | 2 |
| ITGB1BP2 | 30 | 2 |
| TPTE | 30 | 2 |
| TWIST1 | 30 | 2 |
| perhexiline | 30 | 2 |
| AKT/S6 kinase | 30 | 2 |
| MAP3K11 | 30 | 2 |
| MAP3K1 | 30 | 2 |
| KCNMA1 | 30 | 2 |
| ITGA5 | 30 | 2 |
| Phe-Asp | 25 | 2 |
| Î±-galactosidase A | 25 | 2 |
| SLC50A1 | 25 | 2 |
| HRAS | 25 | 2 |
| SIRT1 | 25 | 2 |
| NOS1 | 25 | 2 |
| CAMK2_complex | 25 | 2 |
| ADIPOQ | 25 | 2 |
| KLF10 | 25 | 2 |
| Male | 25 | 2 |
| HDAC_II | 25 | 2 |
| arachidonic acid | 25 | 2 |
| MYL | 25 | 2 |
| PI3K | 25 | 2 |
| translation | 25 | 2 |
| BCL2L1 | 25 | 2 |
| transcription, DNA-templated | 25 | 2 |
| PTS | 25 | 2 |
| TGFB | 25 | 2 |
| Ras-ERK | 25 | 2 |
| LAEI | 25 | 2 |
| calcium oxalate | 25 | 2 |
| methemoglobin | 20 | 2 |
| sodium(1+) | 20 | 2 |
| EPHX2 | 20 | 2 |
| SRXN1 | 20 | 2 |
| necrotic cell death | 20 | 2 |
| indol-3-ylmethylamine | 20 | 2 |
| TP53 | 20 | 2 |
| SLPI | 20 | 2 |
| ASGR1 | 20 | 2 |
| transforming growth factor Î˛1 | 20 | 2 |
| spironolactone | 20 | 2 |
| angiotensin II receptor | 20 | 2 |
| phosphoinositol-4 | 20 | 2 |
| CMA1 | 20 | 2 |
| 3-methylcholanthrene | 20 | 2 |
| nuclear erythroid-2 like factor-2 | 20 | 2 |
| nuclear erythroid related factor-2 | 20 | 2 |
| rutherfordium atom | 20 | 2 |
| diltiazem | 20 | 2 |
| deep palmoplantar creases | 20 | 2 |
| cardiac promoter | 20 | 2 |
| cardiac myosin-binding protein-C | 20 | 2 |
| iron atom | 15 | 2 |
| progesterone | 15 | 2 |
| calcium binding | 15 | 2 |
| finasteride | 15 | 2 |
| Ranolazine attenuated ISO | 15 | 2 |
| PVC1 | 15 | 2 |
| PTH1-34 | 15 | 2 |
| N-ELC | 15 | 2 |
| ICa-L | 15 | 2 |
| ECV-CMR | 15 | 2 |
| DNMT1 | 15 | 2 |
| Alu | 15 | 2 |
| Adiponectin receptor | 15 | 2 |
| T-box transcription factor | 15 | 1 |
| TBX15 | 15 | 1 |
| LAMP-2A | 15 | 1 |
| gallic acid | 15 | 1 |
| ROS1 | 15 | 1 |
| Mutant sarcomeric protein | 15 | 1 |
| SLC7A11 | 15 | 1 |
| FTH1 | 15 | 1 |
| CYCS | 10 | 1 |
| olmesartan | 10 | 1 |
| EGFP | 10 | 1 |
| CRISPR | 10 | 1 |
| RAF1-PPP1CB complexes | 10 | 1 |
| LZTR1 | 10 | 1 |
| Y20C-MYPN | 10 | 1 |
| CA8 | 10 | 1 |
| JP2-S165F | 10 | 1 |
| TRPC3 | 10 | 1 |
| Ig | 10 | 1 |
| TF | 10 | 1 |
| EGF | 10 | 1 |
| heparin | 10 | 1 |
| V1-V4 | 10 | 1 |
| twi | 10 | 1 |
| GDNF | 10 | 1 |
| Sik1 | 10 | 1 |
| vanadium dioxide | 10 | 1 |
| RANGAP1 | 10 | 1 |
| GCK-MODY | 10 | 1 |
| GCK | 10 | 1 |
| coenzyme Q10 | 5 | 1 |
| PDSS2 | 5 | 1 |
| type 2 Berardinelli-Seip Congenital Lipodystrophy | 5 | 1 |
| BSCL2 | 5 | 1 |
| Mazzanti | 5 | 1 |
| SHOC2 | 5 | 1 |
| proteolysis | 5 | 1 |
| VWF | 5 | 1 |
| transthyretin amyloidosis | 5 | 1 |
| TTR | 5 | 1 |
| Mitochondrial Disease | 5 | 1 |
| SCO1 | 5 | 1 |
| CDH2 | 5 | 1 |
| KCNN2 | 5 | 1 |
| HDAC2 | 5 | 1 |
| CK2 | 5 | 1 |
| Asp-Asp | 5 | 1 |
| furosemide | 5 | 1 |
| myocardial triglyceride | 5 | 1 |
| dihydrogen | 5 | 1 |
| TTN | 5 | 1 |
| RBM20 | 5 | 1 |
| integrin Î˛1 | 0 | 1 |
| ADAM17 | 0 | 1 |
| DTNBP1 | 0 | 1 |
| TRIM32 | 0 | 1 |
| Fabry Disease | 0 | 1 |
| Î±-Galactosidase A | 0 | 1 |
| Î±-myosin heavy chain Cre | 0 | 1 |
| recombinase | 0 | 1 |
| Placebos | 0 | 1 |
| receptor-Î± | 0 | 1 |
| Vasopressin | 0 | 1 |
| Î˛-Adrenergic Receptor | 0 | 1 |
| valsartan | 0 | 1 |
| valsartan+LBQ | 0 | 1 |
| FABP6 | 0 | 1 |
| c.111+1Gâ†’A | 0 | 1 |
| LEOPARD Syndrome | 0 | 1 |
| Shp2 loss-of-function (LOF) mutations | 0 | 1 |
| cell death | 0 | 1 |
| SRS11-92 | 0 | 1 |
| TXN | 0 | 1 |
| R120GCRYAB | 0 | 1 |

**INDRA-assembled PubMed+PathwayCommons HCM model**

| name | _wks_percentile_bucket | _wkshell |
| --- | --- | --- |
| VEGFA | 95 | 151 |
| ICAM1 | 95 | 150 |
| MYPN | 95 | 149 |
| MT2A | 95 | 148 |
| ACTC1 | 95 | 147 |
| TPM1 | 95 | 146 |
| PTEN | 95 | 145 |
| VCL | 95 | 144 |
| CRYAB | 95 | 143 |
| LAMP2 | 95 | 142 |
| JPH2 | 95 | 141 |
| ANKRD1 | 95 | 140 |
| NPPB | 95 | 139 |
| GLA | 95 | 138 |
| FLNC | 95 | 137 |
| TTR | 95 | 136 |
| TNNC1 | 95 | 135 |
| CSRP3 | 95 | 134 |
| NEXN | 95 | 133 |
| TNNI3 | 95 | 132 |
| FHL1 | 95 | 131 |
| PRKAG2 | 95 | 130 |
| GNAS | 95 | 129 |
| TNNT2 | 95 | 128 |
| DES | 95 | 127 |
| doxorubicin | 95 | 126 |
| CASQ2 | 95 | 125 |
| MYOZ2 | 95 | 124 |
| MYL3 | 95 | 123 |
| ACTN2 | 95 | 122 |
| valproic acid | 95 | 121 |
| NOG | 95 | 120 |
| SB 431542 | 95 | 120 |
| dorsomorphin | 95 | 120 |
| GAA | 95 | 119 |
| triclosan | 95 | 118 |
| MYH6 | 95 | 118 |
| LDB3 | 95 | 117 |
| MYH7 | 95 | 116 |
| MYL2 | 95 | 115 |
| estradiol | 95 | 114 |
| calcium(2+) | 95 | 113 |
| PLN | 95 | 112 |
| Tobacco Smoke Pollution | 95 | 111 |
| all-trans-retinoic acid | 95 | 110 |
| bisphenol A | 95 | 109 |
| benzo[a]pyrene | 95 | 109 |
| paracetamol | 95 | 108 |
| copper(II) sulfate | 95 | 107 |
| GNAI3 | 95 | 107 |
| phenylmercury acetate | 95 | 106 |
| JQ1 | 95 | 105 |
| dexamethasone | 95 | 104 |
| cyclosporin A | 95 | 104 |
| cobalt dichloride | 95 | 104 |
| silicon dioxide | 95 | 104 |
| 2,3,7,8-tetrachlorodibenzodioxine | 95 | 104 |
| cisplatin | 95 | 104 |
| sodium arsenite | 95 | 103 |
| 4-hydroxy-17beta-estradiol | 85 | 102 |
| nickel sulfate | 95 | 102 |
| Genipin | 85 | 102 |
| kaempferide | 85 | 102 |
| bortezomib | 90 | 102 |
| isotretinoin | 85 | 102 |
| nanoribbon | 90 | 102 |
| lithium-7 atom | 85 | 102 |
| bufalin | 85 | 102 |
| progestin | 85 | 102 |
| propidium | 85 | 102 |
| fludrocortisone | 85 | 102 |
| raloxifene hydrochloride | 90 | 102 |
| alpha-hexylcinnamaldehyde | 85 | 102 |
| minocycline | 85 | 102 |
| chloromethylisothiazolinone | 85 | 102 |
| mifepristone | 85 | 102 |
| methylisothiazolinone | 90 | 102 |
| motexafin gadolinium | 85 | 102 |
| toluene 2,4-diisocyanate | 90 | 102 |
| 5-aza-2'-deoxycytidine | 90 | 102 |
| N-formyl-L-methionyl-L-leucyl-L-phenylalanine | 85 | 102 |
| rosiglitazone | 90 | 102 |
| bucladesine | 85 | 102 |
| indometacin | 95 | 102 |
| cobalt(2+) | 90 | 102 |
| methionine sulfoximine | 85 | 102 |
| emetine | 85 | 102 |
| imidurea | 90 | 102 |
| embelin | 85 | 102 |
| 1,4-phenylenediamine | 85 | 102 |
| diclofenac | 90 | 102 |
| alvocidib | 85 | 102 |
| doxazosin | 85 | 102 |
| crocidolite asbestos | 95 | 102 |
| 9-cis-retinoic acid | 85 | 102 |
| zoledronic acid | 90 | 102 |
| 5-fluorouracil | 90 | 102 |
| TAXOTERE | 85 | 102 |
| trichostatin A | 95 | 102 |
| paclitaxel | 90 | 102 |
| vorinostat | 90 | 102 |
| digoxin | 85 | 102 |
| afimoxifene | 90 | 102 |
| mercury dibromide | 90 | 102 |
| nimesulide | 90 | 102 |
| methotrexate | 90 | 102 |
| (-)-demecolcine | 90 | 102 |
| Deguelin | 85 | 102 |
| lithium chloride | 90 | 102 |
| brefeldin A | 85 | 102 |
| endosulfan | 85 | 102 |
| 3-isobutyl-1-methyl-9H-xanthine | 95 | 102 |
| emodin | 85 | 102 |
| digitoxigenin | 85 | 102 |
| digoxigenin | 85 | 102 |
| quartz | 90 | 102 |
| tremolite asbestos | 85 | 102 |
| daunorubicin | 85 | 102 |
| asbestos | 90 | 102 |
| dequalinium | 85 | 102 |
| celecoxib | 90 | 102 |
| cyclophosphamide | 90 | 102 |
| 1,10-phenanthroline | 85 | 102 |
| okadaic acid | 85 | 102 |
| curcumin | 85 | 102 |
| desferrioxamine B | 85 | 102 |
| coumestrol | 90 | 102 |
| dacarbazine | 85 | 102 |
| phorbol 13-acetate 12-myristate | 90 | 102 |
| antirheumatic drug | 95 | 102 |
| cadmium dichloride | 95 | 102 |
| amitrole | 85 | 102 |
| cephaeline | 85 | 102 |
| manganese(0) | 85 | 102 |
| paraquat | 90 | 102 |
| dibutyl phthalate | 90 | 102 |
| quinomethionate | 85 | 102 |
| 4,4'-sulfonyldiphenol | 95 | 102 |
| 1,3-benzothiazole-2-thiol | 85 | 102 |
| carbamazepine | 95 | 102 |
| (-)-anisomycin | 85 | 102 |
| capsaicin | 80 | 102 |
| sunitinib | 80 | 102 |
| candesartan | 80 | 102 |
| iron chelator | 80 | 102 |
| vitamin E | 90 | 102 |
| gadodiamide | 80 | 102 |
| gadolinium trichloride | 80 | 102 |
| chlorpromazine | 80 | 102 |
| chromium(6+) | 90 | 102 |
| butein | 80 | 102 |
| budesonide | 85 | 102 |
| Proscillaridin | 80 | 102 |
| oxaliplatin | 85 | 102 |
| DL-Isoprenaline hydrochloride | 85 | 102 |
| benzethonium chloride | 80 | 102 |
| alclometasone dipropionate | 80 | 102 |
| betamethasone | 80 | 102 |
| gallic acid | 85 | 102 |
| diarsenic trioxide | 95 | 102 |
| benzo[a]pyrene diol epoxide I | 95 | 102 |
| vanadium oxoanion | 90 | 102 |
| copper(0) | 90 | 102 |
| beclomethasone | 80 | 102 |
| baicalein | 80 | 102 |
| tunicamycin | 85 | 102 |
| nocodazole | 80 | 102 |
| arsenite(1-) | 85 | 102 |
| nickel dichloride | 90 | 102 |
| 2-methoxy-17beta-estradiol | 80 | 102 |
| flavanones | 80 | 102 |
| mibolerone | 80 | 102 |
| 2,4-D | 80 | 102 |
| 1-chloro-2,4-dinitrobenzene | 90 | 102 |
| 3,3',4,4',5-pentachlorobiphenyl | 90 | 102 |
| 1,1,1-Trichloro-2-(o-chlorophenyl)-2-(p-chlorophenyl)ethane | 80 | 102 |
| Calcimycin | 80 | 102 |
| rutin | 80 | 102 |
| tamibarotene | 80 | 102 |
| methylmercury chloride | 95 | 102 |
| gadopentetate dimeglumine | 80 | 102 |
| nickel atom | 95 | 102 |
| genistein | 90 | 102 |
| 2,2'-bipyridine | 80 | 102 |
| resveratrol | 90 | 102 |
| alpha-aluminium oxide | 90 | 102 |
| oxytetracycline | 80 | 102 |
| divanadium pentaoxide | 80 | 102 |
| camptothecin | 80 | 102 |
| cycloheximide | 90 | 102 |
| antimycin A | 80 | 102 |
| arsenic atom | 90 | 102 |
| folic acid | 85 | 102 |
| zinc atom | 90 | 102 |
| reactive oxygen species | 90 | 102 |
| amiloride | 80 | 102 |
| oxygen atom | 95 | 102 |
| aflatoxin B1 | 95 | 102 |
| lead atom | 95 | 102 |
| cadmium atom | 90 | 102 |
| ascorbic acid | 80 | 102 |
| acetylcysteine | 90 | 102 |
| apigenin | 80 | 102 |
| urethane | 90 | 102 |
| puromycin | 80 | 102 |
| methylarsonous acid | 90 | 102 |
| calcitriol | 90 | 102 |
| cortisol | 80 | 102 |
| gemcitabine | 90 | 102 |
| glucose | 90 | 102 |
| p-chloromercuribenzoic acid | 90 | 102 |
| nornicotine | 80 | 102 |
| aristolochic acid | 80 | 102 |
| progesterone | 95 | 102 |
| formaldehyde | 95 | 102 |
| lipopolysaccharide | 90 | 102 |
| quercetin | 95 | 102 |
| hydrogen peroxide | 95 | 102 |
| DDT | 85 | 102 |
| palmatine | 80 | 102 |
| luteolin | 80 | 102 |
| juglone | 80 | 102 |
| butanal | 95 | 102 |
| acrolein | 85 | 102 |
| dinophysistoxin 1 | 85 | 102 |
| ethylisopropylamiloride | 80 | 102 |
| cobalt atom | 85 | 102 |
| lanatoside C | 80 | 102 |
| meclocycline | 80 | 102 |
| entinostat | 95 | 102 |
| tyrphostin B42 | 80 | 102 |
| disodium selenite pentahydrate | 85 | 102 |
| sodium chloride | 90 | 102 |
| amiodarone | 90 | 102 |
| ozone | 80 | 102 |
| naphthoquinone | 80 | 102 |
| 7,12-dimethyltetraphene | 85 | 102 |
| methylmercury compound | 80 | 102 |
| hexachlorocyclohexane | 80 | 102 |
| quinolone | 80 | 102 |
| citral | 90 | 102 |
| 12-HETE | 85 | 102 |
| nicotine | 80 | 102 |
| 4-methylumbelliferyl acetate | 80 | 102 |
| cinnamyl alcohol | 80 | 102 |
| albendazole | 90 | 102 |
| adenosine | 85 | 102 |
| atrazine | 90 | 102 |
| prostaglandin E2 | 80 | 102 |
| Azaspiracid | 85 | 102 |
| avobenzone | 80 | 102 |
| lysophosphatidic acid | 80 | 102 |
| zearalenone | 80 | 102 |
| MT1L mRNA | 80 | 102 |
| Particulate Matter | 95 | 102 |
| Strophanthins | 80 | 102 |
| Plant Extracts | 90 | 102 |
| Dust | 90 | 102 |
| Dietary Fats | 80 | 102 |
| Chrysenes | 80 | 102 |
| Vehicle Emissions | 90 | 102 |
| Air Pollutants, Occupational | 90 | 102 |
| Aerosols | 90 | 102 |
| Meglumine Antimoniate | 80 | 102 |
| 2-amino-8-(4-(2-hydroxyethoxy)cyclohexyl)-6-(6-methoxypyridin-3-yl)-4-methylpyrido(2,3-d)pyrimidin-7(8H)-one | 85 | 102 |
| GSK1210151A | 90 | 102 |
| molibresib | 80 | 102 |
| incobotulinumtoxinA | 95 | 102 |
| jinfukang | 95 | 102 |
| xiongshao | 90 | 102 |
| punarnavine | 80 | 102 |
| N'-(11H-indolo(3,2-c)quinolin-6-yl)-N,N-dimethylethane-1,2-diamine | 80 | 102 |
| auriculasin | 80 | 102 |
| 103D5R | 80 | 102 |
| (E)-4-((2-N-(4-methoxybenzenesulfonyl)amino)stilbazole)1-oxide | 80 | 102 |
| 15,16-dihydrotanshinone I | 80 | 102 |
| Coptidis rhizoma extract | 80 | 102 |
| Progesterone Congeners | 80 | 102 |
| norcantharidin | 80 | 102 |
| epigallocatechin gallate | 90 | 102 |
| N-(deoxyguanosin-8-yl)-1-aminopyrene | 80 | 102 |
| heat stable toxin (E coli) | 80 | 102 |
| fludarabine | 85 | 102 |
| Plant Oils | 85 | 102 |
| tris(1,3-dichloro-2-propyl)phosphate | 95 | 102 |
| Humic Substances | 80 | 102 |
| coralyne | 80 | 102 |
| Drugs, Chinese Herbal | 90 | 102 |
| Drupanin | 80 | 102 |
| PIK3CA | 85 | 102 |
| NSC668394 | 80 | 102 |
| PAX4 | 95 | 102 |
| PCI 5002 | 85 | 102 |
| 7-(benzylamino)-1,3,4,8-tetrahydropyrrolo(4,3,2-de)quinolin-8(1H)-one | 80 | 102 |
| ATF4 | 85 | 102 |
| 2-hydroxymethyl-2-methoxymethylazabicyclo(2.2.2)octan-3-one | 85 | 102 |
| Fedratinib | 80 | 102 |
| 4-(3-(2-propyl-3-hydroxy-4-acetyl)phenoxy)propyloxyphenoxy acetic acid | 80 | 102 |
| fluor-edenite | 80 | 102 |
| cobalt-nickel-chromium-molybdenum alloy | 80 | 102 |
| delta(4)-tibolone | 80 | 102 |
| MAZ | 95 | 102 |
| 7,8-diacetoxy-4-methylcoumarin | 80 | 102 |
| macrophage stimulatory lipopeptide 2 | 80 | 102 |
| 1,2-diethyl-3-hydroxypyridin-4-one | 80 | 102 |
| JUN | 90 | 102 |
| polyhexamethyleneguanidine | 80 | 102 |
| titanium alloy (TiAl6V4) | 80 | 102 |
| cadmium acetate | 80 | 102 |
| HIF1A | 85 | 102 |
| exochelins | 80 | 102 |
| cobaltiprotoporphyrin | 90 | 102 |
| urushiol | 80 | 102 |
| 4-methyl-7-diethylaminocoumarin | 80 | 102 |
| GSK-J4 | 85 | 102 |
| PTGS2 | 80 | 102 |
| PPARG | 80 | 102 |
| EGF | 85 | 102 |
| IL1A | 90 | 102 |
| IL18 | 90 | 102 |
| IGF1 | 80 | 102 |
| REPIN1 | 95 | 102 |
| IL6 | 90 | 102 |
| AGT | 90 | 102 |
| TNF | 90 | 102 |
| TGFB1 | 95 | 102 |
| CXCR4 | 75 | 102 |
| CTNNB1 | 85 | 102 |
| CYP4Z1 | 75 | 102 |
| SULF1 | 75 | 102 |
| CGB3 | 75 | 102 |
| CREB3L1 | 75 | 102 |
| CGB8 | 75 | 102 |
| CGB5 | 75 | 102 |
| CAT | 75 | 102 |
| CXCL12 | 75 | 102 |
| Verbenalin | 75 | 102 |
| SOD2 | 90 | 102 |
| thapsigargin | 85 | 102 |
| valsartan | 85 | 102 |
| troglitazone | 90 | 102 |
| triamcinolone | 75 | 102 |
| thimerosal | 85 | 102 |
| thalidomide | 85 | 102 |
| sirolimus | 85 | 102 |
| MEF2A | 95 | 102 |
| simvastatin | 90 | 102 |
| silver(0) | 95 | 102 |
| arachidonic acid | 85 | 102 |
| sodium dodecyl sulfate | 90 | 102 |
| semaxanib | 75 | 102 |
| INS | 95 | 102 |
| Rhein | 75 | 102 |
| N,N,N',N'-tetrakis(2-pyridylmethyl)ethylenediamine | 85 | 102 |
| pyrvinium | 75 | 102 |
| copper(II) chloride dihydrate | 90 | 102 |
| propranolol | 85 | 102 |
| prednisolone | 75 | 102 |
| icariside II | 75 | 102 |
| piroxicam | 90 | 102 |
| losartan | 85 | 102 |
| carbon black | 90 | 102 |
| pioglitazone | 90 | 102 |
| 2,4-dibromophenyl 2,4,5-tribromophenyl ether | 90 | 102 |
| Enterolactone | 85 | 102 |
| phenobarbital | 90 | 102 |
| royal jelly | 75 | 102 |
| Temsirolimus | 75 | 102 |
| Nonylphenol | 75 | 102 |
| SB 203580 | 85 | 102 |
| Nonidet P-40 | 75 | 102 |
| diallyl trisulfide | 75 | 102 |
| 2-(2-amino-3-methoxyphenyl)chromen-4-one | 75 | 102 |
| octreotide | 75 | 102 |
| ethanol | 90 | 102 |
| ochratoxin A | 90 | 102 |
| copper(II) oxide | 90 | 102 |
| fenbendazole | 75 | 102 |
| Niclosamide | 90 | 102 |
| potassium chromate | 95 | 102 |
| 5-chloro-7-iodoquinolin-8-ol | 75 | 102 |
| WIN 55212-2 | 75 | 102 |
| (25R)-cholest-5-ene-3beta,26-diol | 90 | 102 |
| ICI 118551 | 85 | 102 |
| norethisterone | 75 | 102 |
| triamcinolone acetonide | 75 | 102 |
| withaferin A | 75 | 102 |
| metoprolol | 75 | 102 |
| metformin | 85 | 102 |
| melittin | 90 | 102 |
| medroxyprogesterone acetate | 85 | 102 |
| Delta(9)-tetrahydrocannabinol | 90 | 102 |
| lycorine | 75 | 102 |
| tofacitinib | 75 | 102 |
| demethoxycurcumin | 75 | 102 |
| andrographolide | 75 | 102 |
| LY294002 | 85 | 102 |
| levonorgestrel | 85 | 102 |
| ciglitazone | 90 | 102 |
| 8-Br-cAMP | 85 | 102 |
| 8-bromo-3',5'-cyclic GMP | 75 | 102 |
| mebendazole | 75 | 102 |
| ruxolitinib | 75 | 102 |
| tert-butyl hydroperoxide | 95 | 102 |
| zerumbone | 90 | 102 |
| topotecan | 75 | 102 |
| 7-ketocholesterol | 85 | 102 |
| nevirapine | 75 | 102 |
| ribavirin | 85 | 102 |
| potassium nitrate | 75 | 102 |
| manganese(II) chloride | 85 | 102 |
| N-methyl-4-phenylpyridinium | 85 | 102 |
| zinc acetate | 90 | 102 |
| canertinib | 75 | 102 |
| romidepsin | 85 | 102 |
| cetrorelix | 75 | 102 |
| leflunomide | 90 | 102 |
| Honokiol | 75 | 102 |
| glucosamine | 75 | 102 |
| arsenic trichloride | 75 | 102 |
| alexidine | 75 | 102 |
| potassium dichromate | 90 | 102 |
| dicrotophos | 75 | 101 |
| TCAP | 75 | 100 |
| ICG 001 | 75 | 100 |
| LEF1 | 75 | 100 |
| TCF3 | 75 | 100 |
| propanal | 75 | 99 |
| C646 compound | 75 | 99 |
| K 7174 | 75 | 98 |
| cytarabine | 75 | 97 |
| MYLK2 | 75 | 96 |
| MYBPC3 | 75 | 95 |
| SRF | 75 | 94 |
| magnetite nanoparticle | 75 | 93 |
| succimer | 75 | 93 |
| HNF4A | 75 | 92 |
| N-(hexanoyl)sphing-4-enine | 75 | 91 |
| Bandrowski's base | 75 | 91 |
| polystyrene polymer | 75 | 91 |
| lysophosphatidylcholine | 70 | 91 |
| ceruletide | 70 | 91 |
| 2,4-dinitroiodobenzene | 70 | 91 |
| hyperforin | 70 | 91 |
| hydralazine | 75 | 91 |
| fexofenadine | 70 | 91 |
| beryllium sulfate | 70 | 91 |
| chromium(3+) trichloride | 70 | 91 |
| diphenylmethane-4,4'-diisocyanate | 70 | 91 |
| gentamicin sulfate | 70 | 91 |
| 1-fluoro-2,4-dinitrobenzene | 70 | 91 |
| bezafibrate | 70 | 91 |
| chrysene | 70 | 91 |
| maleic anhydride | 75 | 91 |
| lucanthone | 70 | 91 |
| immunological adjuvant | 70 | 91 |
| ferrous oxide | 70 | 91 |
| ferric oxide | 70 | 91 |
| eugenol | 70 | 91 |
| erythromycin | 70 | 91 |
| sulforaphane | 70 | 91 |
| ammonium salt | 70 | 91 |
| imatinib | 70 | 91 |
| ritonavir | 70 | 91 |
| dicloxacillin | 70 | 91 |
| chloropicrin | 70 | 91 |
| oleanolic acid | 75 | 91 |
| cimetidine | 70 | 91 |
| 24(S),25-epoxycholesterol | 70 | 91 |
| atorvastatin | 70 | 91 |
| perfluorooctane-1-sulfonic acid | 75 | 91 |
| pyrene | 70 | 91 |
| spiro compound | 70 | 91 |
| tetrachloro-1,4-benzoquinone | 70 | 91 |
| phthalic anhydride | 70 | 91 |
| carbonyl compound | 75 | 91 |
| zinc oxide | 75 | 91 |
| L-phenylalanyl group | 70 | 91 |
| tibolone | 70 | 91 |
| Mizolastine | 70 | 91 |
| 2,2',4,4',5,5'-hexachlorobiphenyl | 70 | 91 |
| 1-nitropyrene | 70 | 91 |
| gold molecular entity | 70 | 91 |
| silver molecular entity | 75 | 91 |
| 3,3',5,5'-tetrabromobisphenol A | 70 | 91 |
| aluminium hydroxide | 70 | 91 |
| nitrogen dioxide | 70 | 91 |
| fluoranthene | 70 | 91 |
| titanium dioxide | 70 | 91 |
| bromobenzene | 70 | 91 |
| CAV3 | 70 | 91 |
| lopinavir | 70 | 91 |
| atazanavir sulfate | 70 | 91 |
| benzalkonium chloride | 70 | 91 |
| resorcinol | 75 | 91 |
| sulfates | 70 | 91 |
| glucocorticoid | 75 | 91 |
| melphalan | 75 | 91 |
| phenanthrene | 70 | 91 |
| deoxycholic acid | 70 | 91 |
| fructose | 70 | 91 |
| sodium fluoride | 70 | 91 |
| S-butyl-DL-homocysteine (S,R)-sulfoximine | 70 | 91 |
| farnesol | 70 | 91 |
| vincristine | 75 | 91 |
| nicotinamide | 70 | 91 |
| (E)-cinnamaldehyde | 75 | 91 |
| hexadecanoic acid | 75 | 91 |
| acetylsalicylic acid | 75 | 91 |
| pimecrolimus | 70 | 91 |
| acteoside | 70 | 91 |
| polyphosphates | 70 | 91 |
| polyphenol | 70 | 91 |
| catechin | 70 | 91 |
| N-methyl-N'-nitro-N-nitrosoguanidine | 75 | 91 |
| (R)-noradrenaline | 70 | 91 |
| polysaccharide | 70 | 91 |
| linalool | 70 | 91 |
| chenodeoxycholic acid | 70 | 91 |
| indican | 70 | 91 |
| lithocholic acid | 70 | 91 |
| zinc diethyldithiocarbamate | 70 | 91 |
| 3,3',4,4'-tetrachlorobiphenyl | 70 | 91 |
| 1,2,5,6,9,10-hexabromocyclododecane | 70 | 91 |
| MMP9 | 75 | 91 |
| Smoke | 75 | 91 |
| Air Pollutants | 75 | 91 |
| 2-methoxy-N-(3-methyl-2-oxo-1,2,3,4-tetrahydroquinazolin-6-yl)benzenesulfonamide | 70 | 91 |
| 10-nitro-oleic acid | 70 | 91 |
| huanglian | 70 | 91 |
| 15-deoxyprostaglandin J2 | 70 | 91 |
| SP 5186 | 70 | 91 |
| N-(3-nitratopivaloyl)cysteine ethyl ester | 70 | 91 |
| Ethinyl Estradiol-Norgestrel Combination | 70 | 91 |
| peracetylated N-azidoacetylmannosamine | 70 | 91 |
| NTN1 | 70 | 91 |
| lipopolysaccharide, E coli O55-B5 | 70 | 91 |
| lipopolysaccharide, Escherichia coli O111 B4 | 70 | 91 |
| lipopolysaccharide, E. coli O26-B6 | 70 | 91 |
| TO-901317 | 70 | 91 |
| AG 1879 | 70 | 91 |
| calfactant | 70 | 91 |
| methanandamide | 70 | 91 |
| cobalt oxide | 70 | 91 |
| desethylamiodarone | 70 | 91 |
| tin protoporphyrin IX | 70 | 91 |
| 9,10-dihydro-9,10-dihydroxybenzo(a)pyrene | 75 | 91 |
| pyromellitic dianhydride | 70 | 91 |
| cyclamen aldehyde | 70 | 91 |
| CXCL8 | 70 | 91 |
| IL1B | 75 | 91 |
| IFNG | 75 | 91 |
| HMOX1 | 70 | 91 |
| HMGB1 | 70 | 91 |
| ALB | 70 | 91 |
| TP53 | 75 | 91 |
| CYP3A4 | 65 | 91 |
| TGFBI | 65 | 91 |
| CCL2 | 65 | 91 |
| ADIPOQ | 75 | 91 |
| vincaleukoblastine sulfate | 65 | 91 |
| ursolic acid | 65 | 91 |
| Triptolide | 75 | 91 |
| thiram | 75 | 91 |
| sulindac | 65 | 91 |
| sulfasalazine | 65 | 91 |
| ketotifen | 65 | 91 |
| Quinupristin-dalfopristin | 65 | 91 |
| panobinostat | 75 | 91 |
| (E)-3-tosylacrylonitrile | 65 | 91 |
| pentanal | 75 | 91 |
| o-anisidine | 65 | 91 |
| fenbuconazole | 65 | 91 |
| Cathelicidin | 65 | 91 |
| peptidoglycan | 65 | 91 |
| GW 4064 | 65 | 91 |
| GW 3965 | 65 | 91 |
| caffeine | 75 | 91 |
| dimethyl fumarate | 65 | 91 |
| flavonoids | 65 | 91 |
| xanthohumol | 65 | 91 |
| cannabidiol | 65 | 91 |
| methapyrilene | 65 | 91 |
| (22R)-22-hydroxycholesterol | 65 | 91 |
| nebivolol | 75 | 91 |
| etoricoxib | 65 | 91 |
| conjugated linoleic acid | 65 | 91 |
| ibuprofen | 65 | 91 |
| heterocyclic compound | 65 | 91 |
| hexamethylene diisocyanate | 75 | 91 |
| (4-amino-1,4-dihydro-3-(2-pyridyl)-5-thioxo-1,2,4-triazole)copper(II) | 65 | 90 |
| 17alpha-ethynylestradiol | 65 | 89 |
| ESRRA | 65 | 88 |
| Leu-Val | 65 | 88 |
| TEAD1 | 65 | 87 |
| methylseleninic acid | 65 | 86 |
| SP1 | 65 | 85 |
| NR3C1 | 65 | 84 |
| MECOM | 65 | 84 |
| PPARA | 65 | 83 |
| FOXO4 | 65 | 83 |
| triadimefon | 65 | 83 |
| 2-methylcholine | 65 | 82 |
| tris(2-butoxyethyl) phosphate | 65 | 81 |
| HSF1 | 65 | 81 |
| etoposide | 65 | 80 |
| ETS2 | 65 | 79 |
| bisphenol F | 65 | 78 |
| HSF2 | 65 | 77 |
| MYOD1 | 65 | 76 |
| ZEB1 | 65 | 76 |
| dasatinib monohydrate | 65 | 76 |
| ctxB | 65 | 75 |
| PITX2 | 65 | 74 |
| tamoxifen | 65 | 73 |
| bis(2-chloroethyl) sulfide | 65 | 72 |
| methamphetamine | 65 | 71 |
| disulfiram | 65 | 70 |
| mercury dichloride | 65 | 70 |
| methyl methanesulfonate | 65 | 70 |
| glyoxal-lysine dimer | 65 | 69 |
| ifosfamide | 65 | 69 |
| hydroxycitronellal | 65 | 69 |
| uranium(0) | 65 | 69 |
| diphenylcyclopropenone | 65 | 69 |
| methyl non-2-ynoate | 65 | 69 |
| amphibole asbestos | 65 | 69 |
| isobutanol | 65 | 69 |
| cadmium sulfate | 65 | 69 |
| zinc dichloride | 65 | 69 |
| lead(2+) | 65 | 69 |
| indirubin-3'-monoxime | 65 | 69 |
| fluorescein 5-isothiocyanate | 65 | 69 |
| cidofovir anhydrous | 65 | 69 |
| dibenzo[a,l]pyrene | 65 | 69 |
| iron trichloride | 65 | 69 |
| zinc sulfate | 65 | 69 |
| N-nitrosodiethylamine | 65 | 69 |
| 3-methylcholanthrene | 65 | 69 |
| tungsten molecular entity | 65 | 69 |
| platinum | 65 | 69 |
| silver(1+) nitrate | 65 | 69 |
| rotenone | 65 | 69 |
| zinc pyrithione | 65 | 69 |
| benzoic acid | 65 | 69 |
| parathion | 65 | 69 |
| chloroacetaldehyde | 65 | 69 |
| oxidonitrogen(1+) | 60 | 69 |
| biochanin A | 65 | 69 |
| kaempferol | 60 | 69 |
| daidzein | 60 | 69 |
| 17beta-hydroxy-5alpha-androstan-3-one | 60 | 69 |
| chromium atom | 65 | 69 |
| dipotassium bis[mu-tartrato(4-)]diantimonate(2-) trihydrate | 65 | 69 |
| genistein 7-O-beta-D-glucoside | 60 | 69 |
| trinitrotoluene | 60 | 69 |
| farnesal | 60 | 69 |
| vanillin | 60 | 69 |
| iron atom | 65 | 69 |
| glycerol | 60 | 69 |
| benzo[d]isothiazol-3-one | 60 | 69 |
| p-methylaminophenyl sulfate | 60 | 69 |
| zidovudine | 65 | 69 |
| Arsenites | 60 | 69 |
| Gasoline | 65 | 69 |
| Grape Seed Proanthocyanidins | 60 | 69 |
| acyline | 60 | 69 |
| seocalcitol | 60 | 69 |
| 4-hydroxy-equilenin | 65 | 69 |
| Ferric Compounds | 60 | 69 |
| bis(cysteinato)zincate(lI) | 60 | 69 |
| bis-N,N-dimethylamino-2-(N-methylpyrrolyl)methyl cyclopentadienyl titanium (IV) | 60 | 69 |
| benzyl cinnamate | 60 | 69 |
| beta-hydroxy simvastatin acid | 60 | 69 |
| pluronic block copolymer p85 | 60 | 69 |
| copper histidine | 60 | 69 |
| quinone methide | 60 | 69 |
| tolmetin glucuronide | 60 | 69 |
| iron(II)-ascorbic acid complex | 60 | 69 |
| zomepirac glucuronide | 60 | 69 |
| gallium nitrate | 60 | 69 |
| 3-dimethylaminopropylamine | 60 | 69 |
| IFNA13 | 65 | 69 |
| IFNA1 | 65 | 69 |
| chromium trinitrate | 60 | 69 |
| Salinomycin | 60 | 69 |
| nitroprusside | 65 | 69 |
| bleomycin A5 | 60 | 69 |
| carbonyl cyanide p-trifluoromethoxyphenylhydrazone | 60 | 69 |
| promegestone | 60 | 69 |
| diethyl maleate | 60 | 69 |
| menadione sulfonic acid | 65 | 69 |
| RUNX1 | 60 | 68 |
| hsa-miR-939-3p | 60 | 67 |
| hsa-miR-93-5p | 60 | 67 |
| hsa-miR-8073 | 60 | 67 |
| hsa-miR-8068 | 60 | 67 |
| hsa-miR-7703 | 60 | 67 |
| hsa-miR-7111-3p | 60 | 67 |
| hsa-miR-7107-5p | 60 | 67 |
| hsa-miR-7106-5p | 60 | 67 |
| hsa-miR-6890-5p | 60 | 67 |
| hsa-miR-6883-5p | 60 | 67 |
| hsa-miR-6881-3p | 60 | 67 |
| hsa-miR-6860 | 60 | 67 |
| hsa-miR-6855-5p | 60 | 67 |
| hsa-miR-6849-5p | 60 | 67 |
| hsa-miR-6849-3p | 60 | 67 |
| hsa-miR-6845-3p | 60 | 67 |
| hsa-miR-6840-3p | 60 | 67 |
| hsa-miR-6829-3p | 60 | 67 |
| hsa-miR-6827-3p | 60 | 67 |
| hsa-miR-6823-3p | 60 | 67 |
| hsa-miR-6811-5p | 60 | 67 |
| hsa-miR-6807-5p | 60 | 67 |
| hsa-miR-6799-5p | 60 | 67 |
| hsa-miR-6791-3p | 60 | 67 |
| hsa-miR-6788-3p | 60 | 67 |
| hsa-miR-6785-5p | 60 | 67 |
| hsa-miR-6780a-5p | 60 | 67 |
| hsa-miR-6780a-3p | 60 | 67 |
| hsa-miR-6779-5p | 60 | 67 |
| hsa-miR-6771-3p | 60 | 67 |
| hsa-miR-6769b-3p | 60 | 67 |
| hsa-miR-6764-5p | 60 | 67 |
| hsa-miR-6747-3p | 60 | 67 |
| hsa-miR-6720-5p | 60 | 67 |
| hsa-miR-670-3p | 60 | 67 |
| hsa-miR-661 | 60 | 67 |
| hsa-miR-6516-5p | 60 | 67 |
| hsa-miR-6512-3p | 60 | 67 |
| hsa-miR-6511b-5p | 60 | 67 |
| hsa-miR-6511a-5p | 60 | 67 |
| hsa-miR-6506-5p | 60 | 67 |
| hsa-miR-650 | 60 | 67 |
| hsa-miR-6499-3p | 60 | 67 |
| hsa-miR-620 | 55 | 67 |
| hsa-miR-619-5p | 55 | 67 |
| hsa-miR-612 | 55 | 67 |
| hsa-miR-6088 | 55 | 67 |
| hsa-miR-6086 | 55 | 67 |
| hsa-miR-5589-5p | 55 | 67 |
| hsa-miR-548e-5p | 55 | 67 |
| hsa-miR-548ah-5p | 55 | 67 |
| hsa-miR-526b-3p | 55 | 67 |
| hsa-miR-520e | 55 | 67 |
| hsa-miR-520d-3p | 55 | 67 |
| hsa-miR-520c-3p | 55 | 67 |
| hsa-miR-520b | 55 | 67 |
| hsa-miR-520a-3p | 55 | 67 |
| hsa-miR-519d-3p | 55 | 67 |
| hsa-miR-5189-5p | 55 | 67 |
| hsa-miR-5186 | 55 | 67 |
| hsa-miR-512-3p | 55 | 67 |
| hsa-miR-5089-5p | 55 | 67 |
| hsa-miR-508-5p | 55 | 67 |
| hsa-miR-5009-3p | 55 | 67 |
| hsa-miR-490-5p | 55 | 67 |
| hsa-miR-4802-3p | 55 | 67 |
| hsa-miR-4796-3p | 55 | 67 |
| hsa-miR-4793-3p | 55 | 67 |
| hsa-miR-4770 | 55 | 67 |
| hsa-miR-4763-5p | 55 | 67 |
| hsa-miR-4756-3p | 55 | 67 |
| hsa-miR-4731-5p | 55 | 67 |
| hsa-miR-4728-5p | 55 | 67 |
| hsa-miR-4727-3p | 55 | 67 |
| hsa-miR-4726-3p | 55 | 67 |
| hsa-miR-4723-3p | 60 | 67 |
| hsa-miR-4684-5p | 55 | 67 |
| hsa-miR-4683 | 55 | 67 |
| hsa-miR-4639-3p | 55 | 67 |
| hsa-miR-455-3p | 55 | 67 |
| hsa-miR-4504 | 55 | 67 |
| hsa-miR-4468 | 55 | 67 |
| hsa-miR-4460 | 55 | 67 |
| hsa-miR-4284 | 60 | 67 |
| hsa-miR-4279 | 60 | 67 |
| hsa-miR-4257 | 55 | 67 |
| hsa-miR-4252 | 55 | 67 |
| hsa-miR-425-5p | 55 | 67 |
| hsa-miR-3977 | 55 | 67 |
| hsa-miR-3928-3p | 55 | 67 |
| hsa-miR-377-5p | 55 | 67 |
| hsa-miR-373-3p | 55 | 67 |
| hsa-miR-372-3p | 55 | 67 |
| hsa-miR-3689c | 55 | 67 |
| hsa-miR-3689b-3p | 55 | 67 |
| hsa-miR-3689a-3p | 55 | 67 |
| hsa-miR-3681-5p | 55 | 67 |
| hsa-miR-3653-5p | 60 | 67 |
| hsa-miR-3612 | 55 | 67 |
| hsa-miR-3609 | 55 | 67 |
| hsa-miR-3591-5p | 55 | 67 |
| hsa-miR-340-5p | 60 | 67 |
| hsa-miR-340-3p | 55 | 67 |
| hsa-miR-335-5p | 60 | 67 |
| hsa-miR-3192-5p | 55 | 67 |
| hsa-miR-3187-5p | 55 | 67 |
| hsa-miR-3183 | 60 | 67 |
| hsa-miR-3170 | 55 | 67 |
| hsa-miR-3163 | 55 | 67 |
| hsa-miR-3156-5p | 55 | 67 |
| hsa-miR-30b-3p | 55 | 67 |
| hsa-miR-302f | 55 | 67 |
| hsa-miR-302e | 55 | 67 |
| hsa-miR-302d-3p | 55 | 67 |
| hsa-miR-302c-3p | 55 | 67 |
| hsa-miR-302b-3p | 55 | 67 |
| hsa-miR-302a-3p | 55 | 67 |
| hsa-miR-2467-3p | 55 | 67 |
| hsa-miR-24-3p | 60 | 67 |
| hsa-miR-221-5p | 55 | 67 |
| hsa-miR-2114-3p | 55 | 67 |
| hsa-miR-20b-5p | 55 | 67 |
| hsa-miR-20a-5p | 55 | 67 |
| hsa-miR-193b-5p | 60 | 67 |
| hsa-miR-1915-3p | 60 | 67 |
| hsa-miR-1910-3p | 55 | 67 |
| hsa-miR-1827 | 55 | 67 |
| hsa-miR-17-5p | 55 | 67 |
| hsa-miR-149-5p | 55 | 67 |
| hsa-miR-149-3p | 55 | 67 |
| hsa-miR-143-3p | 55 | 67 |
| hsa-miR-133b | 55 | 67 |
| hsa-miR-133a-3p | 60 | 67 |
| hsa-miR-1285-3p | 55 | 67 |
| hsa-miR-1281 | 55 | 67 |
| hsa-miR-128-3p | 60 | 67 |
| hsa-miR-1273h-5p | 55 | 67 |
| hsa-miR-1273g-3p | 55 | 67 |
| hsa-miR-1273f | 60 | 67 |
| hsa-miR-1270 | 55 | 67 |
| hsa-miR-1261 | 55 | 67 |
| hsa-miR-124-3p | 60 | 67 |
| hsa-miR-1238-3p | 55 | 67 |
| hsa-miR-1234-3p | 55 | 67 |
| hsa-miR-122-3p | 55 | 67 |
| hsa-miR-106b-5p | 55 | 67 |
| hsa-miR-106a-5p | 55 | 67 |
| hsa-miR-1-3p | 60 | 67 |
| HNF1A | 60 | 67 |
| STAT5A | 55 | 67 |
| ESR2 | 55 | 66 |
| PAX2 | 55 | 65 |
| CEBPA | 55 | 65 |
| sarin | 50 | 64 |
| GATA1 | 50 | 63 |
| MEIS1 | 50 | 62 |
| beta-naphthoflavone | 50 | 62 |
| SREBF1 | 50 | 61 |
| Vitallium | 50 | 60 |
| 2,7-dihydroxynaphthalene | 50 | 60 |
| 2-methyl-2H-pyrazole-3-carboxylic acid (2-methyl-4-o-tolylazophenyl)amide | 50 | 60 |
| HOXA4 | 50 | 60 |
| GATA4 | 50 | 60 |
| EGR1 | 50 | 60 |
| NF1 | 50 | 60 |
| dimethylarsinous acid | 50 | 59 |
| RREB1 | 50 | 59 |
| hsa-miR-34a-5p | 50 | 58 |
| OR5I1 | 50 | 58 |
| SF1 | 50 | 57 |
| TCF4 | 50 | 57 |
| hsa-miR-497-5p | 50 | 56 |
| hsa-miR-21-5p | 50 | 56 |
| YY1 | 50 | 56 |
| testosterone | 50 | 55 |
| hsa-miR-92b-3p | 50 | 55 |
| hsa-miR-92a-3p | 50 | 55 |
| hsa-miR-7151-3p | 50 | 55 |
| hsa-miR-5095 | 50 | 55 |
| MAPK14 | 50 | 55 |
| hsa-miR-32-5p | 50 | 55 |
| hsa-miR-195-5p | 50 | 55 |
| hsa-miR-16-5p | 50 | 55 |
| hsa-miR-15b-5p | 50 | 55 |
| hsa-miR-15a-5p | 50 | 55 |
| Arsenates | 50 | 55 |
| NR2F2 | 50 | 55 |
| MYOG | 50 | 55 |
| MYC | 50 | 55 |
| GABPA | 50 | 55 |
| GABPB2 | 50 | 55 |
| ZNF384 | 50 | 55 |
| TFAP2C | 50 | 55 |
| elesclomol | 50 | 55 |
| ELK1 | 50 | 55 |
| SOX9 | 50 | 54 |
| SPI1 | 50 | 53 |
| ethyl methanesulfonate | 50 | 52 |
| PRKCA | 50 | 51 |
| TFCP2 | 50 | 51 |
| ESR1 | 50 | 51 |
| hsa-miR-375 | 50 | 50 |
| ONECUT1 | 50 | 50 |
| GCM1 | 50 | 50 |
| cylindrospermopsin | 50 | 50 |
| Oxyquinoline sulfate | 50 | 50 |
| PRKACA | 50 | 49 |
| POU1F1 | 50 | 48 |
| EGR2 | 50 | 48 |
| cladribine | 50 | 47 |
| flavanone | 50 | 47 |
| methylmercury(1+) | 50 | 47 |
| lovastatin | 50 | 47 |
| Butylbenzyl phthalate | 50 | 47 |
| carmustine | 50 | 47 |
| cucurbitacin E | 50 | 47 |
| clothianidin | 50 | 47 |
| hydrazone | 50 | 47 |
| vanadyl sulfate | 50 | 47 |
| perfluorooctanoic acid | 50 | 47 |
| methylarsonic acid | 50 | 47 |
| dimethyl sulfoxide | 50 | 47 |
| butyric acid | 50 | 47 |
| estriol | 50 | 47 |
| retinoid | 50 | 47 |
| calciol | 50 | 47 |
| hsa-miR-9500 | 50 | 47 |
| tributylstannane | 50 | 47 |
| hsa-miR-95-5p | 50 | 47 |
| hsa-miR-892a | 50 | 47 |
| hsa-miR-876-3p | 50 | 47 |
| hsa-miR-8064 | 50 | 47 |
| hsa-miR-7107-3p | 50 | 47 |
| hsa-miR-7-5p | 50 | 47 |
| hsa-miR-7-2-3p | 50 | 47 |
| hsa-miR-7-1-3p | 50 | 47 |
| hsa-miR-6875-5p | 50 | 47 |
| hsa-miR-6838-3p | 50 | 47 |
| hsa-miR-6835-3p | 50 | 47 |
| hsa-miR-6834-5p | 50 | 47 |
| hsa-miR-6832-5p | 50 | 47 |
| hsa-miR-6831-5p | 50 | 47 |
| hsa-miR-6831-3p | 45 | 47 |
| hsa-miR-6828-3p | 45 | 47 |
| hsa-miR-6825-3p | 45 | 47 |
| hsa-miR-6761-3p | 45 | 47 |
| hsa-miR-6753-3p | 45 | 47 |
| hsa-miR-6503-5p | 45 | 47 |
| hsa-miR-6133 | 45 | 47 |
| hsa-miR-6130 | 45 | 47 |
| hsa-miR-6129 | 45 | 47 |
| hsa-miR-6127 | 45 | 47 |
| hsa-miR-6079 | 45 | 47 |
| hsa-miR-6077 | 45 | 47 |
| hsa-miR-6071 | 45 | 47 |
| hsa-miR-580-5p | 45 | 47 |
| hsa-miR-5695 | 45 | 47 |
| hsa-miR-5688 | 45 | 47 |
| hsa-miR-5590-3p | 45 | 47 |
| hsa-miR-548g-3p | 45 | 47 |
| hsa-miR-5196-5p | 45 | 47 |
| hsa-miR-5196-3p | 45 | 47 |
| gossypetin | 45 | 47 |
| hsa-miR-5087 | 45 | 47 |
| hsa-miR-495-3p | 45 | 47 |
| hsa-miR-4793-5p | 45 | 47 |
| hsa-miR-4747-5p | 45 | 47 |
| DAPK1 | 45 | 47 |
| hsa-miR-4703-5p | 45 | 47 |
| hsa-miR-4684-3p | 45 | 47 |
| hsa-miR-4533 | 45 | 47 |
| hsa-miR-4527 | 45 | 47 |
| hsa-miR-4510 | 45 | 47 |
| deoxynivalenol | 45 | 47 |
| hsa-miR-4432 | 45 | 47 |
| hsa-miR-4422 | 45 | 47 |
| hsa-miR-4419a | 45 | 47 |
| hsa-miR-3942-5p | 45 | 47 |
| hsa-miR-3927-3p | 45 | 47 |
| hsa-miR-378a-5p | 45 | 47 |
| hsa-miR-369-3p | 45 | 47 |
| hsa-miR-3685 | 45 | 47 |
| Uranium Compounds | 45 | 47 |
| hsa-miR-3679-5p | 45 | 47 |
| hsa-miR-367-3p | 45 | 47 |
| hsa-miR-3663-5p | 45 | 47 |
| hsa-miR-363-3p | 45 | 47 |
| hsa-miR-3605-5p | 45 | 47 |
| hsa-miR-3202 | 45 | 47 |
| phenethyl isothiocyanate | 45 | 47 |
| hsa-miR-3188 | 45 | 47 |
| dihydroartemisinin | 45 | 47 |
| hsa-miR-3174 | 45 | 47 |
| hsa-miR-3145-5p | 45 | 47 |
| hsa-miR-3126-5p | 45 | 47 |
| hsa-miR-3119 | 50 | 47 |
| hsa-miR-30e-5p | 45 | 47 |
| hsa-miR-30d-5p | 45 | 47 |
| hsa-miR-30c-5p | 45 | 47 |
| hsa-miR-30b-5p | 45 | 47 |
| hsa-miR-30a-5p | 45 | 47 |
| hsa-miR-29b-1-5p | 45 | 47 |
| N-(3-((2-hydroxynaphthalen-1-ylmethylene)amino)phenyl)-2-phenylpropionamide | 45 | 47 |
| hsa-miR-299-5p | 45 | 47 |
| hsa-miR-25-3p | 45 | 47 |
| monoisoamyl-2,3-dimercaptosuccinate | 45 | 47 |
| benzenesulfonamide | 45 | 47 |
| hsa-miR-155-3p | 45 | 47 |
| hsa-miR-145-3p | 45 | 47 |
| hsa-miR-142-5p | 45 | 47 |
| hsa-miR-139-5p | 45 | 47 |
| POU2F1 | 45 | 47 |
| PAX5 | 50 | 47 |
| 3-oxo-29-noroleana-1,9(11),12-trien-2,20-dicarbonitrile | 45 | 47 |
| excavatolide B | 45 | 47 |
| DLBS 1425 | 45 | 47 |
| hsa-miR-1185-5p | 45 | 47 |
| hsa-miR-10b-5p | 45 | 47 |
| 1-(methylimino-N-oxy)-6-(2-morpholinoethoxy)-3-phenyl-1H-indene-2-carboxylic acid ethyl ester | 45 | 47 |
| MYB | 50 | 47 |
| 4,4',4''-(4-propyl-((1)H)-pyrazole-1,3,5-triyl) tris-phenol | 45 | 47 |
| candoxin | 45 | 47 |
| hsa-let-7c-3p | 45 | 47 |
| MIR21 mRNA | 45 | 47 |
| ETS1 | 45 | 47 |
| E2F1 | 45 | 47 |
| DDIT3 | 50 | 47 |
| EBF2 | 45 | 47 |
| ZIC3 | 50 | 47 |
| AR | 50 | 47 |
| ZBTB14 | 45 | 47 |
| EP300 | 45 | 47 |
| STAT4 | 45 | 47 |
| decabromodiphenyl ether | 45 | 47 |
| fenhexamid | 45 | 47 |
| fludioxonil | 45 | 47 |
| Tributyltin oxide | 45 | 47 |
| microcystin-LR | 45 | 47 |
| lupeol | 45 | 47 |
| carnosic acid | 45 | 47 |
| 2,3-bis(4-hydroxyphenyl)propionitrile | 45 | 47 |
| levosimendan | 40 | 46 |
| 17beta-hydroxy-17-methylestra-4,9,11-trien-3-one | 45 | 46 |
| mono(2-ethylhexyl) phthalate | 40 | 46 |
| microcystin RR | 40 | 46 |
| hsa-miR-98-5p | 40 | 46 |
| hsa-miR-8065 | 40 | 46 |
| hsa-miR-6836-5p | 40 | 46 |
| hsa-miR-6754-5p | 40 | 46 |
| hsa-miR-6722-3p | 40 | 46 |
| hsa-miR-656-5p | 40 | 46 |
| hsa-miR-6504-3p | 40 | 46 |
| hsa-miR-6132 | 40 | 46 |
| hsa-miR-559 | 40 | 46 |
| hsa-miR-4756-5p | 40 | 46 |
| hsa-miR-4739 | 40 | 46 |
| hsa-miR-4700-3p | 40 | 46 |
| hsa-miR-4500 | 40 | 46 |
| hsa-miR-4458 | 40 | 46 |
| hsa-miR-4441 | 40 | 46 |
| hsa-miR-4270 | 40 | 46 |
| hsa-miR-320b | 40 | 46 |
| hsa-miR-320a | 40 | 46 |
| hsa-miR-222-3p | 40 | 46 |
| hsa-miR-196a-5p | 40 | 46 |
| hsa-miR-1909-3p | 40 | 46 |
| hsa-miR-1321 | 40 | 46 |
| hsa-miR-130b-5p | 40 | 46 |
| hsa-miR-125b-5p | 40 | 46 |
| hsa-let-7i-5p | 40 | 46 |
| hsa-let-7g-5p | 40 | 46 |
| hsa-let-7f-5p | 40 | 46 |
| hsa-let-7e-5p | 40 | 46 |
| hsa-let-7d-5p | 40 | 46 |
| hsa-let-7c-5p | 40 | 46 |
| hsa-let-7b-5p | 40 | 46 |
| hsa-let-7a-5p | 40 | 46 |
| FOXF2 | 40 | 46 |
| ELF2 | 40 | 46 |
| VDR | 40 | 46 |
| MRTFA | 40 | 46 |
| STAT3 | 40 | 46 |
| phosphoinositol-4 | 40 | 46 |
| DCM | 40 | 46 |
| azetidinecarboxylic acid | 40 | 45 |
| 4-hydroxyphenyl retinamide | 40 | 45 |
| usnic acid | 40 | 45 |
| carvedilol | 40 | 45 |
| Aurin | 40 | 45 |
| iopamidol | 40 | 45 |
| cocaine | 40 | 45 |
| thiostrepton | 40 | 45 |
| tetrachloromethane | 40 | 45 |
| MK2i peptide | 40 | 45 |
| 6-OH-BDE-47 | 40 | 45 |
| N-((5-(3-(1-benzylpiperidin-4-yl)propoxy)-1-methyl-1H-indol-2-yl)methyl)-N-methylprop-2-yn-1-amine | 40 | 45 |
| GTF2A2 | 40 | 45 |
| bromoacetate | 40 | 45 |
| GTF2A1 | 40 | 45 |
| EGR3 | 40 | 45 |
| ZIC2 | 40 | 45 |
| ZIC1 | 40 | 45 |
| NQO1 | 40 | 45 |
| STAT6 | 40 | 45 |
| BRCA1 | 40 | 45 |
| 2,6-dichloroindophenol | 40 | 45 |
| bisphenol AF | 40 | 45 |
| sulindac sulfide | 40 | 45 |
| celastrol | 40 | 45 |
| sodium tungstate | 40 | 45 |
| polychlorobiphenyl | 40 | 44 |
| hsa-miR-8485 | 40 | 44 |
| RFX1 | 40 | 44 |
| CEBPD | 40 | 44 |
| SP3 | 40 | 44 |
| NFE2L1 | 40 | 43 |
| MAFG | 40 | 43 |
| dicarboxylic acid | 40 | 42 |
| bicalutamide | 40 | 42 |
| hsa-miR-873-5p | 40 | 42 |
| hsa-miR-6838-5p | 40 | 42 |
| iron molecular entity | 40 | 42 |
| hsa-miR-6746-3p | 40 | 42 |
| hsa-miR-606 | 40 | 42 |
| hsa-miR-548x-5p | 40 | 42 |
| hsa-miR-548p | 40 | 42 |
| hsa-miR-548g-5p | 40 | 42 |
| hsa-miR-548f-5p | 40 | 42 |
| hsa-miR-548c-3p | 40 | 42 |
| hsa-miR-548aj-5p | 40 | 42 |
| hsa-miR-4537 | 40 | 42 |
| hsa-miR-451b | 40 | 42 |
| hsa-miR-4438 | 40 | 42 |
| hsa-miR-424-5p | 40 | 42 |
| hsa-miR-3914 | 35 | 42 |
| hsa-miR-377-3p | 35 | 42 |
| N-(6-AMINOHEXYL)-5-CHLORO-1-NAPHTHALENESULFONAMIDE | 35 | 42 |
| hsa-miR-3152-3p | 35 | 42 |
| hsa-miR-27a-3p | 35 | 42 |
| ON 01910 | 35 | 42 |
| uranyl acetate | 35 | 42 |
| hsa-miR-140-3p | 35 | 42 |
| hsa-miR-1276 | 35 | 42 |
| hsa-miR-103a-3p | 35 | 42 |
| MAF | 35 | 42 |
| LRRK2 | 35 | 42 |
| trifloxystrobin | 35 | 42 |
| glycogen | 40 | 42 |
| Alu | 35 | 42 |
| autophagy | 40 | 42 |
| ammonium hexachloroplatinate | 35 | 41 |
| geldanamycin | 35 | 40 |
| verteporfin | 35 | 40 |
| ampicillin | 35 | 40 |
| hsa-miR-181a-5p | 35 | 40 |
| tobacco tar | 35 | 40 |
| darinaparsin | 35 | 40 |
| ETV4 | 35 | 40 |
| VSX1 | 35 | 40 |
| YAP1 | 35 | 40 |
| ╬▒ | 35 | 40 |
| diquat | 35 | 40 |
| ATP12A | 35 | 39 |
| rifampicin zwitterion | 35 | 39 |
| trichloroethene | 35 | 38 |
| hsa-miR-8082 | 35 | 38 |
| hsa-miR-8079 | 35 | 38 |
| hsa-miR-8057 | 35 | 38 |
| hsa-miR-7641 | 35 | 38 |
| hsa-miR-6879-3p | 35 | 38 |
| hsa-miR-6805-3p | 35 | 38 |
| hsa-miR-6773-3p | 35 | 38 |
| hsa-miR-6730-3p | 35 | 38 |
| hsa-miR-6514-5p | 35 | 38 |
| hsa-miR-6513-3p | 35 | 38 |
| hsa-miR-640 | 35 | 38 |
| hsa-miR-603 | 35 | 38 |
| hsa-miR-5698 | 35 | 38 |
| hsa-miR-5693 | 35 | 38 |
| hsa-miR-5691 | 35 | 38 |
| hsa-miR-525-5p | 35 | 38 |
| hsa-miR-520a-5p | 35 | 38 |
| hsa-miR-5193 | 35 | 38 |
| hsa-miR-504-3p | 35 | 38 |
| hsa-miR-500b-3p | 35 | 38 |
| hsa-miR-488-5p | 35 | 38 |
| hsa-miR-4789-3p | 35 | 38 |
| hsa-miR-4771 | 35 | 38 |
| hsa-miR-4534 | 35 | 38 |
| hsa-miR-4430 | 35 | 38 |
| hsa-miR-4309 | 35 | 38 |
| hsa-miR-4294 | 35 | 38 |
| hsa-miR-3941 | 35 | 38 |
| hsa-miR-3652 | 35 | 38 |
| hsa-miR-362-3p | 35 | 38 |
| hsa-miR-329-3p | 35 | 38 |
| hsa-miR-3198 | 35 | 38 |
| hsa-miR-3194-3p | 35 | 38 |
| hsa-miR-3180-5p | 35 | 38 |
| hsa-miR-3166 | 35 | 38 |
| hsa-miR-3158-5p | 35 | 38 |
| hsa-miR-3135b | 35 | 38 |
| hsa-miR-17-3p | 35 | 38 |
| hsa-miR-143-5p | 35 | 38 |
| hsa-miR-1289 | 35 | 38 |
| hsa-miR-1273h-3p | 35 | 38 |
| hsa-miR-1245a | 35 | 38 |
| hsa-miR-122-5p | 35 | 38 |
| hsa-miR-1205 | 35 | 38 |
| hsa-miR-1184 | 35 | 38 |
| MAX | 35 | 38 |
| KLF12 | 35 | 38 |
| NFE2L2 | 35 | 38 |
| FOXN1 | 35 | 38 |
| 5-HETE | 35 | 37 |
| chrysotile | 35 | 37 |
| dipyridamole | 35 | 37 |
| idarubicin | 35 | 37 |
| beta-adrenergic antagonist | 35 | 37 |
| EC 3.4.15.1 (peptidyl-dipeptidase A) inhibitor | 35 | 37 |
| Temocapril hydrochloride | 35 | 37 |
| dehydroepiandrosterone | 35 | 37 |
| atenolol | 35 | 37 |
| prostaglandin E1 | 35 | 37 |
| BAEC protocol | 35 | 37 |
| CHOP protocol | 35 | 37 |
| NR1I2 | 35 | 37 |
| F2 | 35 | 37 |
| EDN1 | 35 | 37 |
| telmisartan | 30 | 37 |
| spironolactone | 35 | 37 |
| Pentoxifylline | 30 | 37 |
| angiotensin receptor antagonist | 30 | 37 |
| sildenafil citrate | 30 | 37 |
| (5Z,8Z,11Z,13E)-15-HETE | 30 | 37 |
| N-nitrosodimethylamine | 30 | 36 |
| antimony(0) | 30 | 36 |
| selenium atom | 30 | 36 |
| hsa-miR-769-5p | 30 | 36 |
| hsa-miR-6856-5p | 30 | 36 |
| mercury atom | 30 | 36 |
| hsa-miR-6758-5p | 30 | 36 |
| hsa-miR-6732-5p | 30 | 36 |
| hsa-miR-615-3p | 30 | 36 |
| hsa-miR-588 | 30 | 36 |
| hsa-miR-548s | 30 | 36 |
| hsa-miR-5096 | 30 | 36 |
| hsa-miR-4781-3p | 30 | 36 |
| hsa-miR-4716-5p | 30 | 36 |
| hsa-miR-4701-5p | 30 | 36 |
| hsa-miR-4435 | 30 | 36 |
| PRKAA1 | 30 | 36 |
| hsa-miR-3187-3p | 30 | 36 |
| migalastat | 30 | 36 |
| hsa-miR-197-3p | 30 | 36 |
| ATF3 | 30 | 36 |
| ARNT | 30 | 36 |
| CUX1 | 30 | 36 |
| XBP1 | 30 | 36 |
| Phe-Asp | 30 | 36 |
| ╬▒-galactosidase A | 30 | 36 |
| TFAP4 | 30 | 36 |
| STAT1 | 30 | 36 |
| 2,3-dimethoxynaphthalene-1,4-dione | 30 | 36 |
| fulvestrant | 30 | 35 |
| actinomycin D | 30 | 35 |
| AKT2 | 30 | 35 |
| AKT1 | 30 | 35 |
| hsa-miR-454-3p | 30 | 35 |
| pyrazolo(3,4-d)pyrimidine | 30 | 35 |
| ATF6 | 30 | 35 |
| GFI1 | 30 | 35 |
| CRX | 30 | 35 |
| TAL1 | 30 | 35 |
| Death | 30 | 35 |
| mitoxantrone | 30 | 34 |
| HCM | 30 | 34 |
| benzylpenicillin | 30 | 33 |
| hsa-miR-6890-3p | 30 | 33 |
| hsa-miR-6872-3p | 30 | 33 |
| hsa-miR-6869-5p | 30 | 33 |
| hsa-miR-6777-3p | 30 | 33 |
| hsa-miR-590-3p | 30 | 33 |
| hsa-miR-512-5p | 30 | 33 |
| hsa-miR-5010-3p | 30 | 33 |
| hsa-miR-4775 | 30 | 33 |
| hsa-miR-4772-3p | 30 | 33 |
| hsa-miR-4735-5p | 30 | 33 |
| hsa-miR-411-5p | 30 | 33 |
| tafamidis | 30 | 33 |
| zinc(0) | 30 | 33 |
| copper atom | 30 | 33 |
| hsa-miR-2355-5p | 30 | 33 |
| hsa-miR-2113 | 30 | 33 |
| hsa-miR-1976 | 30 | 33 |
| hsa-miR-1304-3p | 30 | 33 |
| PBX1 | 30 | 33 |
| CART1 | 30 | 33 |
| FOXA2 | 30 | 33 |
| FOXO3 | 30 | 33 |
| FOXC1 | 30 | 33 |
| CEBPB | 30 | 33 |
| FOXA3 | 30 | 33 |
| FOXA1 | 30 | 33 |
| transthyretin amyloidosis | 30 | 33 |
| UBP1 | 30 | 32 |
| hsa-miR-513c-3p | 30 | 31 |
| hsa-miR-513a-3p | 30 | 31 |
| hsa-miR-4743-3p | 30 | 31 |
| hsa-miR-4732-5p | 30 | 31 |
| hsa-miR-4652-3p | 30 | 31 |
| hsa-miR-3686 | 30 | 31 |
| hsa-miR-3606-3p | 30 | 31 |
| felodipine | 30 | 31 |
| trifluoperazine | 30 | 31 |
| dihydroxy(stearato)aluminium | 30 | 31 |
| bepridil | 30 | 31 |
| calcium atom | 30 | 31 |
| hsa-miR-3137 | 30 | 31 |
| 5-[1-(3,4-Dimethoxy-Benzoyl)-1,2,3,4-Tetrahydro-Quinolin-6-Yl]-6-Methyl-3,6-Dihydro-[1,3,4]Thiadiazin-2-One | 30 | 31 |
| hsa-miR-20b-3p | 30 | 31 |
| AMPK | 30 | 31 |
| PRH2 | 30 | 31 |
| cell differentiation | 30 | 31 |
| SMAD3 | 25 | 30 |
| HRAS | 25 | 30 |
| ROCK1 | 25 | 29 |
| SRC | 25 | 28 |
| Proteasome | 25 | 28 |
| PKA | 25 | 28 |
| hsa-miR-6875-3p | 25 | 27 |
| hsa-miR-6804-5p | 25 | 27 |
| hsa-miR-6755-5p | 25 | 27 |
| hsa-miR-569 | 25 | 27 |
| GSK3B | 25 | 27 |
| hsa-miR-4659b-3p | 25 | 27 |
| hsa-miR-4659a-3p | 25 | 27 |
| hsa-miR-4650-3p | 25 | 27 |
| NRAS | 25 | 27 |
| KRAS | 25 | 27 |
| hsa-miR-3132 | 25 | 27 |
| hsa-miR-197-5p | 25 | 27 |
| hsa-miR-103a-2-5p | 25 | 27 |
| GTF3A | 25 | 27 |
| FOXO1 | 25 | 27 |
| AHR | 25 | 27 |
| PTF1A | 25 | 27 |
| TFDP1 | 25 | 27 |
| TFAP2A | 25 | 27 |
| SRY | 25 | 27 |
| homeostatic process | 25 | 27 |
| trimellitic anhydride | 25 | 26 |
| testosterone undecanoate | 25 | 26 |
| RRN3 | 25 | 26 |
| gardiquimod | 25 | 26 |
| PPP1R13L | 25 | 26 |
| SLC33A1 | 25 | 26 |
| KDM4A | 25 | 26 |
| COX | 25 | 26 |
| PRKCE | 25 | 25 |
| PRKCD | 25 | 25 |
| STK4 | 25 | 25 |
| AT1 receptor | 25 | 25 |
| EN1 | 25 | 25 |
| ADIPOR1 | 25 | 25 |
| Actin | 25 | 25 |
| perphenazine | 25 | 25 |
| alprenolol | 25 | 24 |
| boric acid | 25 | 24 |
| 3-cyanoalanine | 25 | 24 |
| 5-azacytidine | 25 | 24 |
| GSK3A | 25 | 24 |
| AURKB | 25 | 24 |
| Environmental Pollutants | 25 | 24 |
| 3-hydroxy-4-prenyl-5-methoxystilbene-2-carboxylic acid | 25 | 24 |
| eltB | 25 | 24 |
| JP8 aviation fuel | 25 | 24 |
| KAT2B | 25 | 24 |
| TGIF | 25 | 24 |
| ETV7 | 25 | 24 |
| ADRB2 | 25 | 24 |
| TBP | 25 | 24 |
| pyrrolidine dithiocarbamate | 25 | 24 |
| (E)-4-hydroxynon-2-enal | 25 | 24 |
| bathocuproine disulfonic acid | 25 | 24 |
| tacrolimus (anhydrous) | 25 | 23 |
| epichlorohydrin | 25 | 23 |
| 7beta-aminocephalosporanic acid | 25 | 23 |
| CDC5L | 25 | 23 |
| TCF12 | 25 | 23 |
| Myosin_complex | 25 | 22 |
| TXNDC5 | 25 | 21 |
| triacetylcellulose | 25 | 20 |
| CAMK2A | 25 | 19 |
| MT19c compound | 25 | 19 |
| phenylephrine | 25 | 19 |
| TG | 25 | 19 |
| BIN1 | 25 | 18 |
| calcium phosphate | 25 | 18 |
| calcium hydrogenphosphate dihydrate | 25 | 18 |
| Calcium Citrate | 25 | 18 |
| KHK-C | 25 | 18 |
| heroin | 25 | 17 |
| hsa-miR-7110-3p | 25 | 17 |
| hsa-miR-6892-3p | 25 | 17 |
| hsa-miR-6887-3p | 25 | 17 |
| hsa-miR-6873-3p | 25 | 17 |
| hsa-miR-6858-3p | 25 | 17 |
| hsa-miR-6826-3p | 25 | 17 |
| hsa-miR-6823-5p | 25 | 17 |
| hsa-miR-6817-3p | 25 | 17 |
| hsa-miR-6795-3p | 25 | 17 |
| hsa-miR-6787-3p | 25 | 17 |
| hsa-miR-6736-3p | 25 | 17 |
| hsa-miR-621 | 25 | 17 |
| hsa-miR-575 | 25 | 17 |
| CSNK2A1 | 25 | 17 |
| hsa-miR-4695-3p | 25 | 17 |
| hsa-miR-4676-5p | 20 | 17 |
| hsa-miR-4524b-3p | 20 | 17 |
| hsa-miR-4524a-3p | 20 | 17 |
| hsa-miR-4485-5p | 20 | 17 |
| hsa-miR-4446-5p | 20 | 17 |
| hsa-miR-3679-3p | 20 | 17 |
| hsa-miR-2276-3p | 20 | 17 |
| POU6F1 | 20 | 17 |
| NR6A1 | 20 | 17 |
| ITGAL | 20 | 17 |
| IRF1 | 20 | 17 |
| HOXA9 | 20 | 17 |
| FOXM1 | 20 | 17 |
| DBP | 20 | 17 |
| CBFA2T3 | 20 | 17 |
| CBFA2T2 | 20 | 17 |
| ZBTB18 | 20 | 17 |
| SOX5 | 20 | 17 |
| RORA | 20 | 17 |
| PRKD1 | 20 | 16 |
| Troponin | 20 | 16 |
| cMyBP-C | 20 | 16 |
| captopril | 20 | 16 |
| SF3B1 | 20 | 16 |
| RASGRP1 | 20 | 15 |
| RASGRF2 | 20 | 15 |
| RASGEF1A | 20 | 15 |
| RASGRP4 | 20 | 15 |
| RAPGEF2 | 20 | 15 |
| RASGRP3 | 20 | 15 |
| Miglitol | 20 | 15 |
| acarbose | 20 | 15 |
| AT2220 | 20 | 15 |
| Dietary Carbohydrates | 20 | 15 |
| BECN1 | 20 | 15 |
| LC3-II/I | 20 | 15 |
| NFE2 | 20 | 15 |
| yessotoxin | 20 | 15 |
| HES1 | 20 | 15 |
| APLN | 20 | 15 |
| Angiotensin-2 | 20 | 15 |
| ERK | 20 | 15 |
| torkinib | 20 | 15 |
| Metallothionein | 20 | 15 |
| p38 | 20 | 14 |
| 2-aminooctadec-4-ene-1,3-diol | 20 | 14 |
| PYR3 | 20 | 14 |
| Cadherin | 20 | 14 |
| MAPK3 | 20 | 14 |
| PPP3 | 20 | 14 |
| Mavacamten | 20 | 14 |
| MYL2-R58Q iPSC-CMs | 20 | 14 |
| Fostamatinib | 20 | 14 |
| 4-[4-(2,5-DIOXO-PYRROLIDIN-1-YL)-PHENYLAMINO]-4-HYDROXY-BUTYRIC ACID | 20 | 14 |
| nickel acetate | 20 | 14 |
| Phenylbutyrates | 20 | 14 |
| NOX4 | 20 | 14 |
| gambierol | 20 | 14 |
| SMAD1 | 20 | 14 |
| 1-hydroxy-2-oxo-3,3-bis(2-aminoethyl)-1-triazene | 20 | 14 |
| HAND1 | 20 | 14 |
| TnC-L29Q construct | 20 | 14 |
| magnesium(2+) | 20 | 14 |
| PSMD4 | 20 | 14 |
| CREB1 | 20 | 14 |
| CAMK | 20 | 14 |
| RXRB | 20 | 14 |
| Troponin_I | 20 | 14 |
| sodium atom | 20 | 14 |
| TNNT1 | 20 | 14 |
| Troponin_T | 20 | 14 |
| transient receptor potential channel | 20 | 14 |
| myosin subfragment 1 | 20 | 14 |
| filament | 20 | 14 |
| Troponin_C | 20 | 14 |
| S1 | 20 | 14 |
| Q510E-SHP2 | 20 | 14 |
| HCM mutations | 20 | 14 |
| quercitrin | 20 | 13 |
| hsa-miR-99a-5p | 20 | 13 |
| MYLK | 20 | 13 |
| CIT | 20 | 13 |
| ROCK2 | 20 | 13 |
| PPP1R12A | 20 | 13 |
| BACH2 | 20 | 13 |
| Collagen | 20 | 13 |
| metabolic process | 20 | 13 |
| adenosine 5'-monophosphate | 20 | 13 |
| PTPN11 | 20 | 12 |
| GDF11 | 20 | 12 |
| frataxin | 20 | 12 |
| ARSA | 20 | 12 |
| GNL3 | 20 | 12 |
| MAPK1 | 20 | 11 |
| DMPK | 15 | 11 |
| LVEF | 15 | 11 |
| corticosterone | 15 | 11 |
| DSP | 15 | 11 |
| MAP2K1 | 15 | 11 |
| TEF | 15 | 11 |
| HIF1 | 15 | 11 |
| SCD | 15 | 11 |
| forskolin | 15 | 10 |
| Pulmonary Disease, Chronic Obstructive | 15 | 10 |
| HSPA8 | 15 | 10 |
| Short-hairpin RNA | 15 | 10 |
| LCZ696 | 15 | 10 |
| hsa-let-7i-3p | 15 | 10 |
| hsa-let-7g-3p | 15 | 10 |
| hsa-let-7f-1-3p | 15 | 10 |
| Anacardic acid | 15 | 10 |
| GATA6 | 15 | 10 |
| MyBP-C | 15 | 10 |
| cardiac myosin-binding protein C. | 15 | 10 |
| LAD1 | 15 | 10 |
| HSPA5 | 15 | 10 |
| Arg-Val | 15 | 10 |
| Tm175 | 15 | 10 |
| dobutamine | 15 | 10 |
| 5alpha-cholestane-3beta,5,6beta-triol | 15 | 10 |
| ╬▓1-adrenergic receptor | 15 | 10 |
| FXN | 15 | 10 |
| cardiac myosin | 15 | 10 |
| mdfA | 15 | 10 |
| MEF2C | 15 | 9 |
| Prenylamine | 15 | 9 |
| adenosine 5'-monophosphate(1+) | 15 | 9 |
| CALR3 | 15 | 9 |
| GCG | 15 | 9 |
| PRRX2 | 15 | 9 |
| KCNMA1 | 15 | 9 |
| testosterone enanthate | 15 | 9 |
| HDAC_II | 15 | 9 |
| NPPA | 15 | 9 |
| recombinational repair | 15 | 9 |
| TnTF72L | 15 | 9 |
| FOS | 15 | 8 |
| c-jun mRNA | 15 | 8 |
| L | 15 | 8 |
| N-acetyl-alpha-neuraminic acid | 15 | 8 |
| Htris | 15 | 8 |
| beta-D-glucose | 15 | 8 |
| N-{3-[4-(3-aminopropyl)piperazin-1-yl]propyl}-3-(beta-D-galactopyranosyloxy)-5-nitrobenzamide | 15 | 8 |
| N-{3-[4-(3-aminopropyl)piperazin-1-yl]propyl}-3-(alpha-D-galactopyranosyloxy)-5-nitrobenzamide | 15 | 8 |
| N-{3-[4-(3-Amino-Propyl)-Piperazin-1-Yl]-Propyl}-3-(2-Thiophen-2-Yl-Acetylamino)-5-(3,4,5-Trihydroxy-6-Hydroxymethyl-Tetrahydro-Pyran-2-Yloxy)-Benzamide | 15 | 8 |
| Metanitrophenyl-Alpha-D-Galactoside | 15 | 8 |
| Bmsc-0013 | 15 | 8 |
| BV4 | 15 | 8 |
| BV3 | 15 | 8 |
| BV2 | 15 | 8 |
| BV1 | 15 | 8 |
| 5-Aminocarbonyl-3-Nitrophenyl-Alpha-D-Galactopyranose | 15 | 8 |
| 3-Amino-4-{3-[2-(2-Propoxy-Ethoxy)-Ethoxy]-Propylamino}-Cyclobut-3-Ene-1,2-Dione | 15 | 8 |
| APLNR | 15 | 8 |
| PNPLA2 | 15 | 8 |
| TP73 | 15 | 8 |
| MEK | 15 | 8 |
| L-thyroxine | 15 | 8 |
| ╬▒- | 15 | 8 |
| MAP2K2 | 15 | 7 |
| SOS1 | 15 | 7 |
| FER | 15 | 7 |
| Fyn | 15 | 7 |
| EGFR | 15 | 7 |
| PRKAG1 | 15 | 7 |
| mlc-4 | 15 | 7 |
| tropomyosin | 15 | 7 |
| MARS1 | 15 | 7 |
| Cicatrix | 15 | 7 |
| RENBP | 15 | 7 |
| NR3C2 | 15 | 7 |
| MB | 15 | 7 |
| Tubulin | 15 | 7 |
| HIF | 15 | 7 |
| Arg-Ser | 15 | 7 |
| fQRS | 15 | 7 |
| ETC_complex_II | 15 | 6 |
| cMyBP-C M-domain | 15 | 6 |
| p.Glu62_Arg68dup | 15 | 6 |
| PRKAA2 | 15 | 6 |
| PPM1A | 15 | 6 |
| SNAP25 | 15 | 6 |
| Khk-A | 15 | 6 |
| beta-D-fructofuranose | 15 | 6 |
| topiramate | 15 | 6 |
| nppb | 15 | 6 |
| MPZL1 | 15 | 6 |
| RGS2 | 15 | 6 |
| NSML | 10 | 6 |
| CFH | 10 | 6 |
| MYH7B | 10 | 6 |
| CS | 10 | 6 |
| diphosphate(4-) | 10 | 5 |
| GNAQ | 10 | 5 |
| Gas | 10 | 5 |
| GAP | 10 | 5 |
| S1 ATPase | 10 | 5 |
| Met-Met | 10 | 5 |
| PRKAB1 | 10 | 5 |
| SCA | 10 | 5 |
| MyBPC | 10 | 5 |
| FHOD3 | 10 | 5 |
| CLEC3B | 10 | 5 |
| cardiac myosin-binding protein-C increases | 10 | 5 |
| cMyBPC | 10 | 5 |
| NS RAF1 | 10 | 5 |
| MSTN | 10 | 5 |
| ERVK-18 | 10 | 5 |
| NAA15 | 10 | 5 |
| mevalonic acid | 10 | 5 |
| ATPase | 10 | 5 |
| FLCN | 10 | 5 |
| glycolytic process | 10 | 5 |
| Integrins | 10 | 5 |
| ADP | 10 | 5 |
| necrotic cell death | 10 | 5 |
| indol-3-ylmethylamine | 10 | 5 |
| cell population proliferation | 10 | 5 |
| Phosphatase | 10 | 5 |
| c.2737+1 | 10 | 5 |
| IVS26 | 10 | 5 |
| PKA-targets | 10 | 4 |
| CaV1.2 channels | 10 | 4 |
| Smad3/4 inhibitor | 10 | 4 |
| PREP | 10 | 4 |
| pCa50 | 10 | 4 |
| phenformin | 10 | 4 |
| CMA1 | 10 | 4 |
| ATP | 10 | 4 |
| Thiodigalactoside | 10 | 4 |
| P-Aminophenyl-Alpha-D-Galactopyranoside | 10 | 4 |
| N-Benzyl-3-(alpha-D-galactopyranosyloxy)benzamide | 10 | 4 |
| N-(2-Morpholin-4-Yl-1-Morpholin-4-Ylmethyl-Ethyl)-3-Nitro-5-(3,4,5-Trihydroxy-6-Hydroxymethyl-Tetrahydro-Pyran-2-Yloxy)-Benzamide | 10 | 4 |
| MYK-461 | 10 | 4 |
| 2-Phenethyl-2,3-Dihydro-Phthalazine-1,4-Dione | 10 | 4 |
| icd | 10 | 4 |
| UNC45B | 10 | 4 |
| F_actin | 10 | 4 |
| Beta-MHC | 10 | 4 |
| ATP7B | 10 | 4 |
| Magnetic Resonance Imaging | 10 | 4 |
| extracellular matrix | 10 | 4 |
| protein folding | 10 | 4 |
| JNK | 10 | 4 |
| chloroform | 10 | 4 |
| COX16 | 10 | 4 |
| MRPL44 | 10 | 4 |
| ACE2 | 10 | 4 |
| RIT1 | 10 | 4 |
| VEGF | 10 | 4 |
| KRT1 | 10 | 4 |
| COA6 | 10 | 4 |
| dioxygen | 10 | 4 |
| indoxyl sulfate | 10 | 4 |
| copper(2+) | 10 | 4 |
| HCFC1 | 10 | 4 |
| p.Y89H | 10 | 4 |
| p.T83P | 10 | 4 |
| p.M90I | 10 | 4 |
| Transforming growth factor ╬▓1 | 10 | 4 |
| myosin heads | 10 | 4 |
| F-actin binding | 10 | 4 |
| cGMP | 10 | 3 |
| PKGI | 10 | 3 |
| Casein kinase II | 10 | 3 |
| ╬╝mol/L | 10 | 3 |
| NAS | 10 | 3 |
| force-holding A┬ĚM┬ĚD complex | 10 | 3 |
| ENG | 10 | 3 |
| BCL2 | 10 | 3 |
| MTOR | 10 | 3 |
| glutathione | 10 | 3 |
| GSSG | 10 | 3 |
| SLD | 10 | 3 |
| beta-MHC | 10 | 3 |
| TnI | 10 | 3 |
| ARVC | 10 | 3 |
| DHPS | 10 | 3 |
| TCFL5 | 5 | 3 |
| CAV1 | 5 | 3 |
| DOX | 5 | 3 |
| salicylhydroxamic acid | 5 | 3 |
| PRKAB2 | 5 | 3 |
| 2-aminoadipic acid | 5 | 3 |
| spermine | 5 | 3 |
| AKT | 5 | 3 |
| RU486 | 5 | 3 |
| ╬Ę | 5 | 3 |
| High-fat-BK ╬▓ | 5 | 3 |
| 11beta-hydroxysteroid dehydrogenase (HSD11B) | 5 | 3 |
| IRS1 | 5 | 3 |
| tropomyosin-actin | 5 | 3 |
| MYH | 5 | 3 |
| disulfur | 5 | 3 |
| Mhc | 5 | 3 |
| RAF1 | 10 | 3 |
| LIAS | 5 | 3 |
| MRAS | 5 | 3 |
| MAPK7 | 5 | 3 |
| apoptotic process | 5 | 3 |
| RYR2 | 5 | 3 |
| CCL11 | 5 | 3 |
| arm | 10 | 3 |
| MYL12A | 5 | 3 |
| MAP1LC3 | 5 | 3 |
| H9c2 | 5 | 3 |
| CA2 | 5 | 3 |
| PTTG1 | 5 | 3 |
| IMMT | 5 | 3 |
| KHK-A | 5 | 3 |
| cNTnC | 5 | 3 |
| MAPK | 5 | 3 |
| RAS | 10 | 3 |
| CYP | 10 | 3 |
| U0126 | 5 | 3 |
| translation | 5 | 3 |
| transcription, DNA-templated | 5 | 3 |
| Val-Ala | 5 | 3 |
| phenazine-1-carboxylate | 5 | 3 |
| HCM genes | 5 | 3 |
| 3',5'-cyclic AMP | 5 | 3 |
| ╬▓-myosin heavy chain | 5 | 3 |
| ╬▓-myosin heavy chain isoforms | 5 | 3 |
| ╬▓-cardiac myosin | 5 | 3 |
| ╬▓-MHC | 5 | 3 |
| ╬▓-MHC isoforms | 5 | 3 |
| TNXB | 5 | 3 |
| ╬▒-MHC | 5 | 3 |
| potassium iodide | 5 | 3 |
| Val-Phe | 5 | 3 |
| beta-D-glucosyl-N-(docosanoyl)sphingosine | 5 | 3 |
| LGE | 5 | 3 |
| INaL | 5 | 3 |
| 3D | 5 | 3 |
| IL-1beta mRNA | 5 | 2 |
| LAMP-2A | 5 | 2 |
| HCM gene | 5 | 2 |
| STK11 | 5 | 2 |
| MYL | 5 | 2 |
| PI3K | 5 | 2 |
| BCL2L1 | 5 | 2 |
| Mir208a | 5 | 2 |
| IHC | 5 | 2 |
| Cvb-D | 5 | 2 |
| LAEI | 5 | 2 |
| methemoglobin | 5 | 2 |
| EPHX2 | 5 | 2 |
| botE | 5 | 2 |
| BoNT/C LC | 5 | 2 |
| botA | 5 | 2 |
| COL16A1 | 5 | 2 |
| SLPI | 5 | 2 |
| ASGR1 | 5 | 2 |
| angiotensin II receptor | 5 | 2 |
| rutherfordium atom | 5 | 2 |
| diltiazem | 5 | 2 |
| valsartan+LBQ | 5 | 2 |
| finasteride | 5 | 2 |
| ICa-L | 5 | 2 |
| Histone | 5 | 2 |
| NSVT- | 5 | 2 |
| PPP1CB | 5 | 2 |
| NAA10 | 5 | 2 |
| IQ1 motif | 5 | 2 |
| ITGB1BP2 | 5 | 2 |
| TPTE | 5 | 2 |
| heparin | 5 | 2 |
| TWIST1 | 5 | 2 |
| perhexiline | 5 | 2 |
| AKT/S6 kinase | 5 | 2 |
| MAP3K11 | 5 | 2 |
| MAP3K1 | 5 | 2 |
| ITGA5 | 5 | 2 |
| DNMT1 | 5 | 2 |
| SLC50A1 | 5 | 2 |
| SIRT1 | 5 | 2 |
| NOS1 | 0 | 2 |
| CAMK2_complex | 0 | 2 |
| KLF10 | 0 | 2 |
| Male | 0 | 2 |
| PTS | 0 | 2 |
| TGFB | 0 | 2 |
| Ras-ERK | 0 | 2 |
| calcium oxalate | 0 | 2 |
| sodium(1+) | 0 | 2 |
| SRXN1 | 0 | 2 |
| transforming growth factor ╬▓1 | 0 | 2 |
| nuclear erythroid-2 like factor-2 | 0 | 2 |
| nuclear erythroid related factor-2 | 0 | 2 |
| deep palmoplantar creases | 0 | 2 |
| cardiac promoter | 0 | 2 |
| cardiac myosin-binding protein-C | 0 | 2 |
| calcium binding | 0 | 2 |
| Ranolazine attenuated ISO | 0 | 2 |
| PVC1 | 0 | 2 |
| PTH1-34 | 0 | 2 |
| N-ELC | 0 | 2 |
| ECV-CMR | 0 | 2 |
| Adiponectin receptor | 0 | 2 |
| CYCS | 0 | 1 |
| olmesartan | 0 | 1 |
| CDH2 | 0 | 1 |
| KCNN2 | 0 | 1 |
| Myosin | 0 | 1 |
| MLCK | 0 | 1 |
| CKAP4 | 0 | 1 |
| ZDHHC2 | 0 | 1 |
| ROS1 | 0 | 1 |
| Mutant sarcomeric protein | 0 | 1 |
| TTN | 0 | 1 |
| RBM20 | 0 | 1 |
| integrin ╬▓1 | 0 | 1 |
| ADAM17 | 0 | 1 |
| DTNBP1 | 0 | 1 |
| TRIM32 | 0 | 1 |
| Vasopressin | 0 | 1 |
| ╬▓-Adrenergic Receptor | 0 | 1 |
| FABP6 | 0 | 1 |
| c.111+1GÔćĺA | 0 | 1 |
| LEOPARD Syndrome | 0 | 1 |
| Shp2 loss-of-function (LOF) mutations | 0 | 1 |
| CREBBP | 0 | 1 |
| 9-ACETYL-2,3,4,9-TETRAHYDRO-1H-CARBAZOL-1-ONE | 0 | 1 |
| EGFP | 0 | 1 |
| CRISPR | 0 | 1 |
| RAF1-PPP1CB complexes | 0 | 1 |
| LZTR1 | 0 | 1 |
| Y20C-MYPN | 0 | 1 |
| CA8 | 0 | 1 |
| JP2-S165F | 0 | 1 |
| TRPC3 | 0 | 1 |
| Ig | 0 | 1 |
| TF | 0 | 1 |
| SLC7A11 | 0 | 1 |
| FTH1 | 0 | 1 |
| V1-V4 | 0 | 1 |
| twi | 0 | 1 |
| GDNF | 0 | 1 |
| Sik1 | 0 | 1 |
| vanadium dioxide | 0 | 1 |
| RANGAP1 | 0 | 1 |
| GCK-MODY | 0 | 1 |
| GCK | 0 | 1 |
| coenzyme Q10 | 0 | 1 |
| PDSS2 | 0 | 1 |
| T-box transcription factor | 0 | 1 |
| TBX15 | 0 | 1 |
| type 2 Berardinelli-Seip Congenital Lipodystrophy | 0 | 1 |
| BSCL2 | 0 | 1 |
| Mazzanti | 0 | 1 |
| SHOC2 | 0 | 1 |
| proteolysis | 0 | 1 |
| VWF | 0 | 1 |
| Mitochondrial Disease | 0 | 1 |
| SCO1 | 0 | 1 |
| HDAC2 | 0 | 1 |
| CK2 | 0 | 1 |
| Asp-Asp | 0 | 1 |
| furosemide | 0 | 1 |
| myocardial triglyceride | 0 | 1 |
| dihydrogen | 0 | 1 |
| Fabry Disease | 0 | 1 |
| ╬▒-Galactosidase A | 0 | 1 |
| ╬▒-myosin heavy chain Cre | 0 | 1 |
| recombinase | 0 | 1 |
| Placebos | 0 | 1 |
| receptor-╬▒ | 0 | 1 |
| cell death | 0 | 1 |
| SRS11-92 | 0 | 1 |
| TXN | 0 | 1 |
| R120GCRYAB | 0 | 1 |
| FAK |  |  |

**Truncated INDRA DB model**

| name | _wks_percentile_bucket | _wkshell |
| --- | --- | --- |
| RYR2 | 95 | 9 |
| Troponin | 95 | 8 |
| CALM | 90 | 8 |
| CAMK2_complex | 90 | 8 |
| Ca2+ | 95 | 8 |
| ATPase | 90 | 7 |
| SCD | 90 | 6 |
| JPH2 | 85 | 6 |
| AICA ribonucleotide | 85 | 6 |
| ERK | 85 | 6 |
| MYL12A | 85 | 6 |
| Troponin_T | 80 | 5 |
| MYLK | 80 | 5 |
| Tropomyosin | 80 | 5 |
| Troponin_C | 80 | 4 |
| GSK3B | 75 | 4 |
| FKBP1B | 75 | 3 |
| CASQ2 | 75 | 3 |
| MAPK | 75 | 3 |
| PPP1R13L | 70 | 3 |
| PKA | 70 | 3 |
| cell population proliferation | 70 | 3 |
| IQ | 65 | 3 |
| TTN | 65 | 3 |
| TnI 1-40 | 65 | 2 |
| MyHC 723 | 65 | 2 |
| PtdIns3P | 60 | 2 |
| cell adhesion | 60 | 2 |
| oleic acid | 60 | 2 |
| Mutated gamma2-AMPK | 60 | 2 |
| CCL19-A57G | 55 | 2 |
| MYL12A-R58Q | 55 | 2 |
| MYL12A-D166V | 55 | 2 |
| triglyceride | 55 | 2 |
| phosphatidyl-L-serine | 50 | 2 |
| OBSCN | 50 | 2 |
| MYK-461 | 50 | 2 |
| MYBPC3 | 50 | 2 |
| Mhc | 45 | 2 |
| PPARGC1A | 45 | 2 |
| ATP | 45 | 2 |
| TM | 40 | 2 |
| MAP2K | 40 | 2 |
| MAPK3 | 40 | 2 |
| DSP | 40 | 2 |
| DES | 35 | 2 |
| AMPK | 35 | 2 |
| MAP2K1 | 35 | 2 |
| AKT | 35 | 2 |
| MEK | 30 | 2 |
| Myosin_complex | 30 | 1 |
| HCM mutations | 30 | 1 |
| cTnI switch region | 30 | 1 |
| CTnI N-terminus | 25 | 1 |
| recombinant beta-MyHC | 25 | 1 |
| CMyBP-Cs | 25 | 1 |
| cardiac myosin motors | 25 | 1 |
| CMLCK | 20 | 1 |
| cellular senescence | 20 | 1 |
| Mutated LMNA | 20 | 1 |
| MYL | 15 | 1 |
| MYH | 15 | 1 |
| Death | 15 | 1 |
| Diltiazem | 15 | 1 |
| lactate | 10 | 1 |
| Glycogen | 10 | 1 |
| FN1 | 10 | 1 |
| MMP3 | 10 | 1 |
| Collagen | 5 | 1 |
| MMP3 | 5 | 1 |
| p38 | 5 | 1 |
| PKC | 5 | 1 |
| TGFB | 0 | 1 |
| AGT | 0 | 1 |
| SERCA | 0 | 1 |
| PLN | 0 | 1 |
| NFAT |  |  |

**INDRA DB HCM model**

| name | _wks_percentile_bucket | _wkshell |
| --- | --- | --- |
| Ca2+ | 95 | 41 |
| Actin | 95 | 40 |
| Troponin_C | 95 | 39 |
| Myosin_complex | 95 | 38 |
| MYL12A | 95 | 37 |
| ATPase | 95 | 36 |
| DCM | 95 | 35 |
| Troponin_T | 95 | 35 |
| JPH2 | 95 | 34 |
| Troponin | 95 | 33 |
| ATP | 95 | 33 |
| Filament | 95 | 33 |
| MYBPC3 | 95 | 32 |
| RYR2 | 95 | 31 |
| ERVK-18 | 95 | 30 |
| TTN | 95 | 29 |
| TnI bound to Troponin_C | 90 | 28 |
| Tm5b | 90 | 28 |
| S195D-A57G | 90 | 28 |
| HCTnI-R145G | 90 | 28 |
| HcTnC | 90 | 28 |
| ChcTnC | 90 | 28 |
| Mutated CCL19 | 90 | 28 |
| Tpm1 | 95 | 28 |
| Site II | 90 | 28 |
| HCM Phenotype | 90 | 28 |
| FBN1 | 90 | 28 |
| Mutated CACNB2 | 90 | 28 |
| Beta-TM protein | 90 | 28 |
| A13T-RLC | 85 | 28 |
| Serca2a | 90 | 28 |
| Mutated MYH7 | 90 | 28 |
| mutation | 85 | 28 |
| HCM mutations | 90 | 28 |
| Blebbistatin | 85 | 28 |
| Beta-MyHC | 95 | 28 |
| Trichostatin A | 85 | 28 |
| TG | 95 | 28 |
| DTNB | 85 | 28 |
| Alpha | 85 | 28 |
| Guanine | 85 | 28 |
| N-carbamoylsarcosine | 90 | 28 |
| Phenazine-1-carboxylate | 85 | 28 |
| RANGAP1 | 85 | 28 |
| cadmium(2+) | 85 | 28 |
| EMD | 90 | 28 |
| ATP2A2 | 85 | 28 |
| KN-93 | 85 | 28 |
| TRIM39 | 90 | 28 |
| N-[2-(4-bromocinnamylamino)ethyl]isoquinoline-5-sulfonamide | 85 | 28 |
| Phospholamban | 85 | 28 |
| CLEC3B | 95 | 28 |
| MCU | 85 | 28 |
| Wnt | 90 | 28 |
| tropomyosin | 90 | 28 |
| Oxalic acid | 90 | 28 |
| MICU2 | 95 | 28 |
| Dantrolene | 85 | 28 |
| BCL2 | 85 | 28 |
| SERCA2a | 95 | 28 |
| SNCG | 90 | 28 |
| Bradykinin | 90 | 28 |
| Diltiazem | 90 | 28 |
| MYLK | 95 | 28 |
| filament | 95 | 28 |
| Nifedipine | 85 | 28 |
| 2-(3,4-dimethoxyphenyl)-5-\{[2-(3,4-dimethoxyphenyl)ethyl](methyl)amino\}-2-(propan-2-yl)pentanenitrile | 90 | 28 |
| Caffeine | 85 | 28 |
| CAMK2_complex | 90 | 28 |
| PLN | 95 | 28 |
| PKA | 95 | 28 |
| MYH7 | 85 | 27 |
| AMPK | 85 | 27 |
| Leu-Val | 85 | 26 |
| CALM | 85 | 25 |
| TNNT1 | 85 | 24 |
| MYH | 85 | 23 |
| OBSCN | 85 | 23 |
| Death | 80 | 22 |
| TPM1 | 80 | 21 |
| TnT | 80 | 21 |
| PRH1 | 80 | 21 |
| cMyBP-C | 80 | 21 |
| TM | 80 | 21 |
| TNNI3 | 80 | 21 |
| Mhc | 80 | 20 |
| IQ | 80 | 20 |
| S195D-M173V-ELC phosphomimetic protein | 80 | 19 |
| mutant beta-cardiac myosin | 80 | 19 |
| KRT3 | 80 | 19 |
| app / (f app + g app | 80 | 19 |
| Lys-Met | 80 | 19 |
| arm | 80 | 19 |
| 272-288 | 75 | 19 |
| measurements | 75 | 19 |
| myosin cross-bridges | 80 | 19 |
| assay | 75 | 19 |
| CCL19 | 80 | 19 |
| myosins | 75 | 19 |
| assays | 75 | 19 |
| ELC | 80 | 19 |
| Myosin | 80 | 19 |
| zip | 75 | 19 |
| hydrolysis | 75 | 19 |
| myosin | 75 | 19 |
| activity | 80 | 19 |
| FOXH1 | 75 | 19 |
| TN | 80 | 19 |
| S1 | 75 | 19 |
| Tropomyosin | 80 | 19 |
| ATPase activity | 80 | 19 |
| CTN | 80 | 19 |
| A13T | 80 | 19 |
| CYP1A1 | 80 | 19 |
| Troponin_I | 80 | 19 |
| MyHC 723 | 75 | 18 |
| cell population proliferation | 75 | 18 |
| phenazine-1-carboxylate | 75 | 17 |
| Ser-Leu | 75 | 16 |
| TNNT2 | 75 | 16 |
| ADP | 75 | 16 |
| PKC | 75 | 16 |
| TnI 1-40 | 75 | 15 |
| PKC pseudo | 75 | 15 |
| PKC-epsilon | 75 | 15 |
| L29 | 75 | 15 |
| cTnI 1-73 | 75 | 15 |
| Azanide | 75 | 15 |
| cNTnC | 75 | 15 |
| Ser23/24 | 75 | 15 |
| PTCH1 | 70 | 15 |
| CRP | 70 | 15 |
| BAG3 | 70 | 15 |
| Phosphorylated Troponin_C | 70 | 15 |
| TnI | 75 | 15 |
| PRKD1 | 70 | 15 |
| MYL12A-A13T | 70 | 14 |
| Adenosine 5'-monophosphate | 70 | 13 |
| thiN | 70 | 13 |
| GSK3B | 70 | 13 |
| Mutant bound to MYL12A | 70 | 12 |
| Mavacamten | 70 | 12 |
| CMyBPC | 70 | 12 |
| vRLC | 70 | 12 |
| motility | 70 | 12 |
| acto | 70 | 12 |
| F_actin | 70 | 12 |
| AKR1A1 | 70 | 12 |
| CMyBP-C | 70 | 11 |
| Heart Rate | 70 | 11 |
| KCNMA1 | 70 | 11 |
| AICA ribonucleotide | 70 | 11 |
| K104E-RLC | 70 | 10 |
| Isopropyl beta-D-thiogalactopyranoside | 70 | 10 |
| SCD | 70 | 10 |
| Phosphorylated MYL12A | 70 | 10 |
| FHC- | 65 | 9 |
| MYK-461 | 65 | 9 |
| FN1 | 65 | 9 |
| glycogen | 65 | 9 |
| MMP3 | 70 | 9 |
| MAPK | 65 | 9 |
| Troponin_T-R92L | 65 | 8 |
| LBD6 | 65 | 8 |
| Ig | 65 | 8 |
| KCNH2 | 65 | 8 |
| p38 | 65 | 8 |
| Glucose | 65 | 8 |
| LAG3 | 65 | 7 |
| TG-I61Q | 65 | 7 |
| High-fat-BK beta1-knockout | 65 | 7 |
| HcTnI | 65 | 7 |
| MYL12A-D94A | 65 | 7 |
| Actin-TM | 65 | 7 |
| Actin-myosin | 65 | 7 |
| gamma 2 non-catalytic subunit | 65 | 7 |
| PPP2CA | 65 | 7 |
| HcTn | 65 | 7 |
| MYL2 | 65 | 7 |
| Ala-Pro | 65 | 7 |
| TCN1 | 65 | 7 |
| acto-S1 | 65 | 7 |
| SDHB | 65 | 7 |
| Blood Pressure | 60 | 7 |
| (-)-epigallocatechin 3-gallate | 60 | 7 |
| Glycogen | 60 | 7 |
| Ramipril | 60 | 7 |
| WtTMEM70 | 60 | 6 |
| Pseudo-phosphorylation | 60 | 6 |
| ion channel | 60 | 6 |
| PtdIns3P | 60 | 6 |
| Phosphatidylinositol phosphate | 60 | 6 |
| Phosphatidylinositol | 60 | 6 |
| p3 | 60 | 6 |
| JMJD6 | 60 | 6 |
| Ischemia | 60 | 6 |
| Inositol | 60 | 6 |
| FKBP1B | 60 | 6 |
| SRI | 60 | 6 |
| ERVK-18-D230N | 60 | 6 |
| Dioleoyl phosphatidylglycerol | 60 | 6 |
| Alpha-MyHC-R403Q | 60 | 6 |
| 1,2-dioleoyl-sn-glycero-3-phospho-L-serine | 60 | 6 |
| Mutated Troponin_T | 60 | 6 |
| homeostatic process | 60 | 6 |
| Mutated gamma2-AMPK | 55 | 6 |
| Triphosphatase activity | 55 | 6 |
| PPP1R13L | 55 | 6 |
| CACNB2 | 55 | 6 |
| myosin cross-bridge | 55 | 6 |
| Perhexiline | 55 | 6 |
| Phosphatidyl-L-serine | 55 | 6 |
| CASQ1 | 55 | 6 |
| PRKAG2 | 55 | 6 |
| CFH | 55 | 6 |
| PIP4K2A | 55 | 6 |
| Isoprenaline | 55 | 6 |
| PPP1R13L | 55 | 6 |
| DSP | 60 | 6 |
| DES | 60 | 6 |
| cell adhesion | 60 | 6 |
| INS | 60 | 6 |
| ATP_synthase | 60 | 6 |
| Dihydrogen | 55 | 6 |
| MTOR | 55 | 6 |
| Sirolimus | 55 | 6 |
| AKT | 55 | 6 |
| TnI-203 | 55 | 5 |
| JNK | 55 | 5 |
| GTF2H2 | 55 | 5 |
| myosin filaments | 55 | 5 |
| extracellular matrix | 55 | 5 |
| RCM phenotype | 55 | 5 |
| MYBPC protein | 50 | 5 |
| NPPA | 50 | 5 |
| Natriuretic_peptide | 50 | 5 |
| MHC-403 | 50 | 5 |
| Tm175 | 50 | 5 |
| LcMyBPC-treated cMyBPC -/- hearts | 50 | 5 |
| CCA1 | 50 | 5 |
| Disease | 50 | 5 |
| Spir | 55 | 5 |
| cell differentiation | 50 | 5 |
| KCNQ1 | 50 | 5 |
| ADRB1 | 50 | 5 |
| RGS2 | 50 | 5 |
| PIMREG | 50 | 5 |
| Mutated HCM | 50 | 5 |
| HCM | 55 | 5 |
| Propranolol | 50 | 5 |
| GYS | 50 | 5 |
| CD36 | 50 | 5 |
| D-glucopyranose 6-phosphate | 50 | 5 |
| sAnk1 | 55 | 5 |
| PKA | 50 | 5 |
| signaling | 50 | 5 |
| cell growth | 50 | 5 |
| transcription, DNA-templated | 50 | 5 |
| Ca+2 | 50 | 5 |
| ThiN | 50 | 5 |
| ERK | 50 | 5 |
| Mutated tropomyosin | 45 | 4 |
| TnI inhibitory peptide | 45 | 4 |
| MYL4 | 45 | 4 |
| Cryge | 45 | 4 |
| beta-Tm | 45 | 4 |
| Angiotensin-2 | 45 | 4 |
| RSK | 45 | 4 |
| Val-Ile | 45 | 4 |
| Kr | 45 | 4 |
| (-)-epicatechin-3-O-gallate | 45 | 4 |
| NAD(+) | 45 | 4 |
| Prazosin | 45 | 4 |
| CHKB | 45 | 4 |
| Creatine | 45 | 4 |
| MAPK3 | 50 | 4 |
| ACE | 45 | 4 |
| SERCA | 45 | 4 |
| FOXO1 | 45 | 4 |
| MEK | 45 | 4 |
| FOXO3 | 45 | 4 |
| MYL | 45 | 4 |
| myosin-binding sites | 45 | 3 |
| type 3 | 45 | 3 |
| beta-R92Q | 45 | 3 |
| S23/24D cTnI | 45 | 3 |
| Protein specific therapeutic targets | 45 | 3 |
| Mutation-induced phosphorylatable Ser-15 RLC site | 40 | 3 |
| MLC2 | 40 | 3 |
| mitochondrial calcium uniporter | 40 | 3 |
| Unc-89 | 40 | 3 |
| HERG-HEK293 | 40 | 3 |
| recombinant beta-MyHC | 40 | 3 |
| heart function HCM-D166V mice | 40 | 3 |
| Leu-Val-Ser | 40 | 3 |
| cMyBP-C truncated mutants | 40 | 3 |
| cMyBP-C mutants | 40 | 3 |
| ATPase inhibitors | 40 | 3 |
| collagen-1alpha | 40 | 3 |
| Alpha galactosidase A | 40 | 3 |
| oleic acid | 40 | 3 |
| rAAV9-pseudotyped viral particles | 40 | 3 |
| MSTN | 40 | 3 |
| PEPCK | 40 | 3 |
| HESX1 | 40 | 3 |
| IL6 | 40 | 3 |
| CCL19-A57G | 40 | 3 |
| beta-myosin heavy chain | 40 | 3 |
| Mutated MYL12A | 40 | 3 |
| MYL12A-R58Q | 40 | 3 |
| MYL12A-D166V | 40 | 3 |
| HRAS | 40 | 3 |
| HHEX | 40 | 3 |
| Alfuzosin | 45 | 3 |
| triglyceride | 40 | 3 |
| beta-MHC | 35 | 3 |
| Mutated PRKAG2 | 35 | 3 |
| UNC | 35 | 3 |
| cisapride | 35 | 3 |
| Mutated LMNA | 35 | 3 |
| Integrins | 35 | 3 |
| GLA | 35 | 3 |
| ELN | 35 | 3 |
| COX | 35 | 3 |
| laminin | 35 | 3 |
| ABCC8 | 35 | 3 |
| ANKRD1 | 35 | 3 |
| Collagen | 35 | 3 |
| MICU1 | 35 | 3 |
| PLN phosphorylated on S16 | 35 | 3 |
| metabolic process | 35 | 3 |
| Spironolactone | 35 | 3 |
| PPARGC1A | 35 | 3 |
| AGT | 35 | 3 |
| EGFR | 35 | 3 |
| Troponin_C bound to Actin | 35 | 2 |
| forkhead | 35 | 2 |
| SERCA2A | 35 | 2 |
| HOPX | 35 | 2 |
| SDHD-D98Y | 35 | 2 |
| Deltasdh4 | 35 | 2 |
| GAST | 30 | 2 |
| rescue HCM mice | 30 | 2 |
| heart performance | 30 | 2 |
| actomyosin function | 30 | 2 |
| quality life | 30 | 2 |
| ERVK-18-E180G | 30 | 2 |
| Plasmids | 30 | 2 |
| P.Arg719Trp] mutation | 30 | 2 |
| Non-phosphorylatable D166V myocardium | 30 | 2 |
| skeletal myosin | 30 | 2 |
| myosin duty | 30 | 2 |
| CM | 30 | 2 |
| biochemical perturbations | 30 | 2 |
| altered | 30 | 2 |
| Mutated MT-ATP6 | 30 | 2 |
| Ig53-FnIII-2 regions | 30 | 2 |
| Ig2-Ig3 | 30 | 2 |
| carbon monoxide | 30 | 2 |
| hcTnI-K206I | 30 | 2 |
| MYL3 | 30 | 2 |
| GSK-3beta hearts | 30 | 2 |
| TRAPPC1 | 30 | 2 |
| Modified FOXO1 | 30 | 2 |
| CooH | 30 | 2 |
| CXCR4 | 30 | 2 |
| CDH1 | 30 | 2 |
| Cardiac phosphorylation | 25 | 2 |
| calcium/calmodulin | 25 | 2 |
| Y20C-MYPN | 25 | 2 |
| Angiotensin II receptors | 25 | 2 |
| ryanodine receptor | 25 | 2 |
| AAV virus A plasmid | 25 | 2 |
| AAV9-S15D therapy | 25 | 2 |
| AAV9-S15D-RLC therapy | 25 | 2 |
| Modified SOD3 | 25 | 2 |
| adenosine 5'-monophosphate | 25 | 2 |
| apoptotic process | 25 | 2 |
| bradykinin | 25 | 2 |
| Fabry Disease | 25 | 2 |
| Modified FMO3 | 25 | 2 |
| CUL3 | 25 | 2 |
| Copper(I) chloride | 25 | 2 |
| Alpha1-adrenergic receptor | 25 | 2 |
| GNAQ | 25 | 2 |
| RAF1 | 25 | 2 |
| KRAS | 25 | 2 |
| sodium(1+) | 25 | 2 |
| kinase domain | 25 | 2 |
| cellular senescence | 25 | 2 |
| Cisapride | 25 | 2 |
| Phe-Asp | 25 | 2 |
| Copper(2+) | 25 | 2 |
| MAP2K | 20 | 2 |
| ADRA | 20 | 2 |
| lactate | 20 | 2 |
| NR3C2 | 20 | 2 |
| MYPN | 20 | 2 |
| PTPN11 | 20 | 2 |
| TGFB | 20 | 2 |
| MAP2K1 | 20 | 2 |
| aldosterone | 20 | 2 |
| TNT | 20 | 2 |
| DeltaG 0 F-U | 20 | 1 |
| Urea | 20 | 1 |
| CA2 | 20 | 1 |
| Mutated Troponin_T | 20 | 1 |
| Heart Defects, Congenital | 20 | 1 |
| TPM1 mutations | 20 | 1 |
| TnI mutant | 20 | 1 |
| Mutated TNNT1 | 20 | 1 |
| molecular mechanisms | 20 | 1 |
| Structural | 20 | 1 |
| MICE | 20 | 1 |
| Strain analysis images | 20 | 1 |
| delivery specific organ | 20 | 1 |
| Specific cell | 20 | 1 |
| mitochondrial complex II | 20 | 1 |
| Mutated SDHD | 20 | 1 |
| majority detrimental induced D166V mutation | 20 | 1 |
| S15D phosphomimic | 15 | 1 |
| metalloproteinase | 15 | 1 |
| S100A4 | 15 | 1 |
| GDP | 15 | 1 |
| RhoGEF domain | 15 | 1 |
| PRKAG2 cardiac syndrome PS is inherited disease | 15 | 1 |
| PRKAG2 mutation | 15 | 1 |
| F max | 15 | 1 |
| Prickle1-a | 15 | 1 |
| DISCUSSION | 15 | 1 |
| P.R870H mutation | 15 | 1 |
| cardiac TnI | 15 | 1 |
| PPP2 | 15 | 1 |
| F max of MyHC | 15 | 1 |
| PP-1 sites | 15 | 1 |
| Naxos | 15 | 1 |
| Pk2157del2 mutation | 15 | 1 |
| isovolumic relaxation constant Tau | 15 | 1 |
| Phosphomimic delivery | 15 | 1 |
| cMyBP-C on A2 | 15 | 1 |
| Phosphatase | 15 | 1 |
| PGC-1 | 15 | 1 |
| PCK1 | 15 | 1 |
| PKC isozyme | 15 | 1 |
| sMyBP-C variant-1 | 15 | 1 |
| Obscurins | 15 | 1 |
| cCTnI | 10 | 1 |
| Myosin_complex bound to Actin | 10 | 1 |
| Performing 2D-electrophoresis | 10 | 1 |
| MW molecular | 10 | 1 |
| Familial HCM | 10 | 1 |
| Mutations encoding sarcomeric contractile proteins | 10 | 1 |
| deleterious TPM1 protein structure | 10 | 1 |
| Mutation | 10 | 1 |
| lipoprotein | 10 | 1 |
| Mutant myosin | 10 | 1 |
| microtubule-severing protein katanin | 10 | 1 |
| Mel-26 | 10 | 1 |
| unique structural charge balance | 10 | 1 |
| Introducing S15D phosphomimic | 10 | 1 |
| young athletes | 10 | 1 |
| Familial HCM is occurring inherited cardiac disease | 10 | 1 |
| cMLCK cardiac myosin motors phosphorylation | 10 | 1 |
| D166V-elicited conformation RLC Ser-15 phosphorylation site | 10 | 1 |
| S15D-RLC phosphomimic construct | 10 | 1 |
| Cytomegalovirus CMV | 10 | 1 |
| human ventricular RLC carrying Ser-15 Aspartic acid mutation | 10 | 1 |
| Cytomegalovirus CMV | 10 | 1 |
| Val-Met | 10 | 1 |
| Cyclosporin A | 10 | 1 |
| cTnI switch region | 10 | 1 |
| CTnI N-terminus | 10 | 1 |
| cardiac myosin motors | 5 | 1 |
| CMLCK | 5 | 1 |
| mutation-R161W | 5 | 1 |
| CDKN1A | 5 | 1 |
| functional structural histological observed HCM-D166V mice | 5 | 1 |
| Cardiac-specific phosphomimic S15D-D166V | 5 | 1 |
| flightless | 5 | 1 |
| Mutated CALM | 5 | 1 |
| beta- myosin heavy chain | 5 | 1 |
| PRH2 | 5 | 1 |
| proband received pacemaker | 5 | 1 |
| AVB | 5 | 1 |
| Unc5c | 5 | 1 |
| ATXN3 | 5 | 1 |
| angiotensin II receptor | 5 | 1 |
| APLNR | 5 | 1 |
| heart function observed | 5 | 1 |
| AAV-S15D-RLC | 5 | 1 |
| intact cardiac function | 5 | 1 |
| AAV9-based S15D-RLC mice | 5 | 1 |
| MYBP3 | 5 | 1 |
| HSPB3 | 5 | 1 |
| phalloidin | 5 | 1 |
| NEB | 5 | 1 |
| cTnI 1-32 | 5 | 1 |
| CNTnC | 5 | 1 |
| CSRP3 | 0 | 1 |
| ACTN2 | 0 | 1 |
| JPH1 | 0 | 1 |
| RYR1 | 0 | 1 |
| sodium atom | 0 | 1 |
| Li+ | 0 | 1 |
| CYCS | 0 | 1 |
| Copper atom | 0 | 1 |
| CPOX | 0 | 1 |
| SCO2 | 0 | 1 |
| necrotic cell death | 0 | 1 |
| ARSA | 0 | 1 |
| ASPP | 0 | 1 |
| TP53 | 0 | 1 |
| beta-receptor | 0 | 1 |
| Metoprolol | 0 | 1 |
| sm | 0 | 1 |
| Phosphatidylcholine | 0 | 1 |
| PPP3 | 0 | 1 |
| MBP | 0 | 1 |
| Alpha-D-glucosyl-(1->4)-alpha-D-mannose | 0 | 1 |
| MET | 0 | 1 |
| HGF | 0 | 1 |
| Lys-Val | 0 | 1 |
| Pyraclofos | 0 | 1 |
| NFAT | 0 | 1 |
| P38 | 0 | 1 |
| RLCs |  |  |
| PI3K |  |  |
| TSC22D3 |  |  |
| JUP |  |  |
| ThiN |  |  |
| MYBPC1 |  |  |
| NFATC3 |  |  |
| PPP1 |  |  |
| TSC1 |  |  |
| THBD |  |  |
| Ryanodine receptor |  |  |
| LC |  |  |
| TNI |  |  |
| RPTOR |  |  |
| MYL12B |  |  |
| TSC2 |  |  |
| IRS1 |  |  |
| RPS6KB1 |  |  |
| RPS6 |  |  |
| RAC |  |  |
| STAT3 |  |  |
