# Supplementary material for: Humans and machines in biomedical knowledge curation: hypertrophic cardiomyopathy molecular mechanisms’ representation
Source: BioData Min. 2021 Oct 2;14:45. doi: 10.1186/s13040-021-00279-2 (PMC8487578; doi:10.1186/s13040-021-00279-2)
Supplement: Supplementary file 3 — Additional file 3. Cooperatively working elements. Cooperatively working elements (functional modules) detected and their likely implications. [file 13040_2021_279_MOESM3_ESM.docx]

**Additional file 3. Cooperatively working elements**

| Tabular manual HCM model,  Page Rank | Members | Cliqueness | # of children | Possible | Relevant for HCM | Likely implication |
| --- | --- | --- | --- | --- | --- | --- |
|  | ADP, ATP, Ca2+, SR Ca_ATPase (SERCA) | 0.833 | 0 | + | + | Ca-homeostasis |
|  | ADP, ATP, SR Ca_ATPase (SERCA) | 1.000 | 0 | + | + | Ca-homeostasis |
| * | TGF-beta, angiotensin II, cardiac fibroblast proliferation, cardiomyocyte hypertrophy, myocardial fibrosis | 0.700 | 2 | + | + | fibrosis |
| Child of * | TGF-beta, angiotensin II, cardiac fibroblast proliferation, cardiomyocyte hypertrophy, myocardial fibrosis, reactive oxygen species (ROS) | 0.600 | 0 | + | + | fibrosis |
| Child of * | AGTR1, TGF-beta, angiotensin II, cardiac fibroblast proliferation, cardiomyocyte hypertrophy, myocardial fibrosis | 0.600 | 0 | + | + | fibrosis |
|  | GATA, MEOX1, active calcineurin, cardiomyocyte hypertrophy | 0.833 | 0 | + | + | hypertrophy |
|  | TGF-beta, angiotensin II, has-mir-29a, myocardial fibrosis, reactive oxygen species (ROS) | 0.700 | 0 | + | + | fibrosis |
|  | Arrhythmias, calmodulin dependent kinase II (CamKII), reactive oxygen species (ROS) | 1.000 | 0 | + | + | arrhythmias |
|  | ATP2A2 (SERCA2), phospholamban, phospholamban-P | 1.000 | 0 | + | + | Ca-homeostasis |
|  | COL1A1, COL1A2, TGF-beta, has-mir-29a, reactive oxygen species (ROS) | 0.800 | 0 | + | + | fibrosis |
|  | COL3A1, TGF-beta, has-mir-29a, reactive oxygen species (ROS) | 0.833 | 0 | + | + | fibrosis |
|  | CCN2, TGFB1, TGFB3, TGF-beta, endothelin 1 (ET-1), reactive oxygen species (ROS) | 0.667 | 0 | + | + | fibrosis |
|  | ERK signaling pathway, TGF-beta, cardiomyocyte hypertrophy, reactive oxygen species (ROS) | 0.833 | 0 | + | + | fibrosis |
|  | COL1A1, COL1A2, TGF-beta, cardiomyocyte hypertrophy, has-mir-29a, myocardial fibrosis | 0.600 | 0 | + | + | fibrosis |
|  | CYTOR, IKKi, cardiomyocyte hypertrophy, has-mir-155 | 0.833 | 0 | + | + | proliferation |
|  | TGF-beta, liver X receptor (LXR), reactive oxygen species (ROS) | 1.000 | 0 | + | + | proliferation |
|  | ACE, angiotensin II, cardiac fibroblast proliferation, cardiomyocyte hypertrophy | 0.833 | 0 | + | + | fibrosis |

| Tabular manual HCM model,  Node degree | Members | Cliqueness | # of children | Possible | Relevant for HCM | Likely implication |
| --- | --- | --- | --- | --- | --- | --- |
| * | Erk signaling pathway, TGF-beta, angiotensin II, cardiomyocyte hypertrophy, has-mir-29a | 0.700 | 2 | + | + | fibrosis |
| Child of * | ACE, Erk signaling pathway, TGF-beta, angiotensin II, cardiomyocyte hypertrophy, has-mir-29a | 0.600 | 0 | + | + | fibrosis |
| Child of * | Erk signaling pathway, TGF-beta, angiotensin II, cardiomyocyte hypertrophy, has-mir-29a, myocardial fibrosis, reactive oxygen species (ROS) | 0.619 | 0 | + | + | fibrosis |
|  | Erk signaling pathway, TGF-beta, endothelin 1 (ET-1), liver X receptor (LXR), myocardial fibrosis, reactive oxygen species (ROS) | 0.600 | 0 | + | + | fibrosis |
|  | Ca2+, active calcineurin, inactive calcineurin | 1.000 | 0 | + | + | hypertrophy |
|  | ADP, ATP, Ca2+, SR Ca-ATPase (SERCA) | 0.833 | 0 | + | + | Ca-homeostasis |
|  | COL1A1, COL3A1, TGF-beta, cardiomyocyte hypertrophy, hsa-mir-29a, myocardial fibrosis | 0.600 | 0 | + | + | fibrosis |
|  | GATA4, MEOX1, active calcineurin, cardiomyocyte hypertrophy | 0.833 | 0 | + | + | hypertrophy |
|  | ACE, TGF-beta, angiotensin II, cardiac fibroblast proliferation, cardiomyocyte hypertrophy, myocardial fibrosis | 0.667 | 0 | + | + | fibrosis |
|  | TGF-beta, angiotensin II, has-mir-29a, myocardial fibrosis, reactive oxygen species (ROS) | 0.700 | 0 | + | + | fibrosis |
|  | CCN2, TGFB1, TGFB3, TGF-beta, endothelin 1 (ET-1), reactive oxygen species (ROS) | 0.667 | 0 | + | + | fibrosis |
|  | Arrhythmias, calmodulin dependent kinase II (CamKII), reactive oxygen species (ROS) | 1.000 | 0 | + | + | arrhythmias |
|  | Erk signaling pathway, TGF-beta, cardiomyocyte hypertrophy, reactive oxygen species (ROS) | 0.833 | 0 | + | + | fibrosis |
|  | CYTOR, IKKi, cardiomyocyte hypertrophy, has-mir-155 | 0.833 | 0 | + | + | proliferation |
|  | ATP2A2 (SERCA2), phospholamban, phospholamban-P | 1.000 | 0 | + | + | Ca-homeostasis |
|  | COL1A1, COL3A1, TGF-beta, has-mir-29a, reactive oxygen species (ROS) | 0.800 | 0 | + | + | fibrosis |
|  | COL1A2, TGF-beta, has-mir-29a, reactive oxygen species (ROS) | 0.833 | 0 | + | + | fibrosis |
|  | TGF-beta, liver receptor (LXR), reactive oxygen species (ROS) | 1.000 | 0 | + | + | proliferation |

| INDRA-assembled PubMed HCM model,  Page Rank | Members | Cliqueness | # of children | Possible | Relevant for HCM | Likely implication |
| --- | --- | --- | --- | --- | --- | --- |
|  | cMyBP-C, Ca2+, filament | 1.000 | 0 | Ambiguous element | | |
|  | Troponin, Troponin I, Ca2+ | 1.000 | 0 | Ambiguous element | | |
|  | Leu_Val, TG, triacetylcellulose | 1.000 | 0 | Exogenous element | | |
|  | HIF, HIF1, KHK-C, SF3B1, alpha | 0.700 | 0 | Ambiguous element | | |
|  | Angiotensin II, GDF11, NPPA, nppb | 0.833 | 0 | + | + | overload induced hypertrophy |
|  | Actin, Troponin, tropomyosin | 1.000 | 0 | + | + | contraction |

| INDRA-assembled PubMed HCM model,  Node degree | Members | Cliqueness | # of children | Possible | Relevant for HCM | Likely implication |
| --- | --- | --- | --- | --- | --- | --- |
|  | HIF, HIF1, KHC-C, SF3B1, alpha | 0.700 | 0 | Ambiguous element | | |
|  | Angiotensin II, GDF11, NPPA, nppb | 0.833 | 0 | + | + | overload induced hypertrophy |
|  | CMyBP-C, Ca2+, filament | 1.000 | 0 | Ambiguous element | | |
|  | Leu-Val, TG, triacetylcellulose | 1.000 | 0 | Exogenous element | | |
|  | Troponin, Troponin I, Ca2+ | 1.000 | 0 | Ambiguous element | | |

| INDRA-assembled PubMed+PathwayCommons HCM model,  Page rank | Members | Cliqueness | # of children | Possible | Relevant for HCM | Likely implication |
| --- | --- | --- | --- | --- | --- | --- |
|  | INS, PTEN, VEGFA, benzo[a]pyrene, diarsenic trioxide, formaldehyde | 0.600 | 0 | Exogenous element | | |
|  | INS, PTEN, TGFB1, VEGFA, benzo[a]pyrene, jinfukang | 0.600 | 0 | Exogenous element | | |
|  | MYH7, NPPB, benzo[a]pyrene, cyclosporine A, daunorubicin, doxorubicin | 0.600 | 0 | Exogenous element | | |
|  | cMyBP-C, Ca2+, filament | 1.000 | 0 | Ambiguous element | | |
|  | Troponin, Troponin I, Ca2+ | 1.000 | 0 | Ambiguous element | | |
|  | Leu-Val, TG, triacetylcellulose | 1.000 | 0 | Exogenous element | | |
|  | HIF, HIF1, KHK-C, SF3B1, alpha | 0.700 | 0 | Ambiguous element | | |
|  | PTEN, VEGFA, benzo[a]pyrene | 1.000 | 0 | Exogenous element | | |
|  | INS, PTEN, VEGFA | 1.000 | 0 | + | + | angiogenesis |
|  | Angiotensin II, GDF11, NPPA, nppb | 0.833 | 0 | + | + | overload induced hypertrophy |
|  | MAP2K1, MAP2K2, MAPK1, MAPK3, SOS1 | 0.700 | 0 | + | + | MAPK signaling pathway |
|  | PTEN, VEGFA, doxorubicin | 1.000 | 0 | Exogenous element | | |
|  | PTEN, VEGFA, thalidomide | 1.000 | 0 | Exogenous element | | |
|  | JQ1, PTEN, VEGFA | 1.000 | 0 | + | + | angiogenesis and hypertrophy |
|  | PTEN, VEGFA, arsenic atom | 1.000 | 0 | Exogenous element | | |
|  | Aerosols, PTEN, VEGFA | 1.000 | 0 | Exogenous element | | |
|  | PTEN, VEGFA, copper(II)sulfate | 1.000 | 0 | Exogenous element | | |
|  | PTEN, VEGFA, methylmercury chloride | 1.000 | 0 | Exogenous element | | |
|  | Actin, Troponin, tropomyosin | 1.000 | 0 | + | + | contraction |
|  | PTEN, VEGFA, indometacin | 1.000 | 0 | Exogenous element | | |
|  | PTEN, Tobacco Smoke Pollution, VEGFA | 1.000 | 0 | Exogenous element | | |
|  | PTEN, VEGFA, cobalt dichloride | 1.000 | 0 | Exogenous element | | |
|  | PTEN, VEGFA, lead atom | 1.000 | 0 | Exogenous element | | |
|  | PTEN, VEGFA, cadmium dichloride | 1.000 | 0 | Exogenous element | | |
|  | PTEN, VEGFA, crocidolite asbestos | 1.000 | 0 | Exogenous element | | |
|  | Niclosamide, PTEN, VEGFA | 1.000 | 0 | Exogenous element | | |
|  | PTEN, VEGFA, ochratoxin A | 1.000 | 0 | Exogenous element | | |
|  | PTEN, VEGFA, sodium arsenite | 1.000 | 0 | Exogenous element | | |
|  | PTEN, VEGFA, methotrexate | 1.000 | 0 | Exogenous element | | |
|  | PTEN, VEGFA, rosiglitazone | 1.000 | 0 | Exogenous element | | |
|  | PTEN, VEGFA, quercetin | 1.000 | 0 | Exogenous element | | |
|  | PTEN, VEGFA, dorsomorphin | 1.000 | 0 | Exogenous element | | |
|  | 5-fluorouracil, PTEN, VEGFA | 1.000 | 0 | Exogenous element | | |
|  | PTEN, VEGFA, diarsenic trioxide | 1.000 | 0 | Exogenous element | | |
|  | PTEN, VEGFA, genistein | 1.000 | 0 | Exogenous element | | |
|  | PTEN, VEGFA, valproic acid | 1.000 | 0 | Exogenous element | | |
|  | PTEN, VEGFA, formaldehyde | 1.000 | 0 | Exogenous element | | |
|  | PTEN, VEGFA, dexamethasone | 1.000 | 0 | Exogenous element | | |
|  | PTEN, VEGFA, bisphenol A | 1.000 | 0 | Exogenous element | | |
|  | PTEN, VEGFA, vorinostat | 1.000 | 0 | Exogenous element | | |
|  | PTEN, VEGFA, urethane | 1.000 | 0 | Exogenous element | | |
|  | PTEN, VEGFA, coumestrol | 1.000 | 0 | Exogenous element | | |
|  | 2,3,7,8-tetrachlorodibenzodioxine, PTEN, VEGFA | 1.000 | 0 | Exogenous element | | |
|  | PTEN, VEGFA, copper (0) | 1.000 | 0 | + | + | angiogenesis and hypertrophy |
|  | PTEN, SB 431542, VEGFA | 1.000 | 0 | Exogenous element | | |
|  | PTEN, VEGFA, all-trans-retinoic acid | 1.000 | 0 | Exogenous element | | |
|  | PTEN, VEGFA, reactive oxygen species | 1.000 | 0 | + | + | angiogenesis and hypertrophy |
|  | PTEN, VEGFA, gemcitabine | 1.000 | 0 | Exogenous element | | |
|  | PTEN, VEGFA, tert-butyl hydroperoxide | 1.000 | 0 | Exogenous element | | |
|  | PTEN, VEGFA, jinfukang | 1.000 | 0 | Exogenous element | | |
|  | PTEN, VEGFA, estradiol | 1.000 | 0 | + | + | angiogenesis and hypertrophy |
|  | PTEN, VEGFA, resveratrol | 1.000 | 0 | Exogenous element | | |
|  | 5-aza-2’deoyxytidine, PTEN, VEGFA | 1.000 | 0 | Exogenous element | | |
|  | PTEN, VEGFA, nickel dichloride | 1.000 | 0 | Exogenous element | | |
|  | NOG, PTEN, VEGFA | 1.000 | 0 | + | + | angiogenesis and hypertrophy |
|  | IL18, PTEN, VEGFA, | 1.000 | 0 | + | + | angiogenesis and hypertrophy |
|  | PTEN, TGFB1, VEGFA | 1.000 | 0 | + | + | angiogenesis and hypertrophy |
|  | PTEN, VEGFA, leflunomide | 1.000 | 0 | Exogenous element | | |
|  | PTEN, VEGFA, diclofenac | 1.000 | 0 | Exogenous element | | |
|  | PTEN, VEGFA, carbamazepine | 1.000 | 0 | Exogenous element | | |
|  | PTEN, VEGFA, nickel sulfate | 1.000 | 0 | Exogenous element | | |

| INDRA-assembled PubMed+PathwayCommons HCM model, Node degree | Members | Cliqueness | # of children | Possible | Relevant for HCM | Likely implication |
| --- | --- | --- | --- | --- | --- | --- |
|  | NOG, PTEN, SB 431542, VEGFA, doxorubicin, valproic acid | 0.600 | 0 | Exogenous element | | |
|  | MYH7, NPPB, benzo[a]pyrene, cyclosporine A, daunorubicin, doxorubicin | 0.600 | 0 | Exogenous element | | |
|  | PTEN, VEGFA, doxorubicin | 1.000 | 0 | Exogenous element | | |
|  | PTEN, VEGFA, valproic acid | 1.000 | 0 | Exogenous element | | |
|  | PTEN, SB 431542, VEGFA | 1.000 | 0 | Exogenous element | | |
|  | PTEN, VEGFA, dorsomorphin | 1.000 | 0 | Exogenous element | | |
|  | NOG, PTEN, VEGFA | 1.000 | 0 | + | + | angiogenesis and hypertrophy |
|  | PTEN, VEGFA, estradiol | 1.000 | 0 | + | + | angiogenesis and hypertrophy |
|  | PTEN, Tobacco Smoke Pollution, VEGFA | 1.000 | 0 | Exogenous element | | |
|  | PTEN, VEGFA, benzo[a]pyrene | 1.000 | 0 | Exogenous element | | |
|  | PTEN, VEGFA, all-trans-retinoic acid | 1.000 | 0 | Exogenous element | | |
|  | PTEN, VEGFA, bisphenol A | 1.000 | 0 | Exogenous element | | |
|  | PTEN, VEGFA, copper(II)sulfate | 1.000 | 0 | Exogenous element | | |
|  | JQ1, PTEN, VEGFA | 1.000 | 0 | + | + | angiogenesis and hypertrophy |
|  | PTEN, VEGFA, cobalt dichloride | 1.000 | 0 | Exogenous element | | |
|  | 2,3,7,8-tetrachlorodibenzodioxine, PTEN, VEGFA | 1.000 | 0 | Exogenous element | | |
|  | PTEN, VEGFA, dexamethasone | 1.000 | 0 | Exogenous element | | |
|  | PTEN, VEGFA, sodium arsenite | 1.000 | 0 | Exogenous element | | |
|  | PTEN, VEGFA, carbamazepine | 1.000 | 0 | Exogenous element | | |
|  | PTEN, VEGFA, jinfukang | 1.000 | 0 | Exogenous element | | |
|  | PTEN, TGFB1, VEGFA | 1.000 | 0 | + | + | angiogenesis and hypertrophy |
|  | PTEN, VEGFA, methylmercury chloride | 1.000 | 0 | Exogenous element | | |
|  | PTEN, VEGFA, diarsenic trioxide | 1.000 | 0 | Exogenous element | | |
|  | PTEN, VEGFA, quercetin | 1.000 | 0 | Exogenous element | | |
|  | INS, PTEN, VEGFA | 1.000 | 0 | + | + | angiogenesis |
|  | PTEN, VEGFA, indometacin | 1.000 | 0 | Exogenous element | | |
|  | PTEN, VEGFA, cadmium dichloride | 1.000 | 0 | Exogenous element | | |
|  | PTEN, VEGFA, nickel sulfate | 1.000 | 0 | Exogenous element | | |
|  | PTEN, VEGFA, tert-butyl hydroperoxide | 1.000 | 0 | Exogenous element | | |
|  | PTEN, VEGFA, lead atom | 1.000 | 0 | Exogenous element | | |
|  | PTEN, VEGFA, crocidolite asbestos | 1.000 | 0 | Exogenous element | | |
|  | PTEN, VEGFA, urethane | 1.000 | 0 | Exogenous element | | |
|  | PTEN, VEGFA, genistein | 1.000 | 0 | Exogenous element | | |
|  | PTEN, VEGFA, vorinostat | 1.000 | 0 | Exogenous element | | |
|  | PTEN, VEGFA, formaldehyde | 1.000 | 0 | Exogenous element | | |
|  | PTEN, VEGFA, methotrexate | 1.000 | 0 | Exogenous element | | |
|  | PTEN, VEGFA, resveratrol | 1.000 | 0 | Exogenous element | | |
|  | Angiotensin II, GDF11, NPPA, nppb | 1.000 | 0 | + | + | overload inducing hypertrophy |
|  | HIF, HIF1, KHK-C, SF3B1, alpha | 0.700 | 0 | Ambiguous element | | |
|  | 17alpha-ethynylestradiol, 3-methylcholanthrene, MT2A | 1.000 | 0 | Exogenous element | | |
|  | MAP2K1, MAP2K2, MAPK1, MAPK3, SOS1 | 0.700 | 0 | + | + | MAPK signaling pathway |
|  | cMyBP-C, Ca2+, filament | 1.000 | 0 | Ambiguous element | | |
|  | PTEN, VEGFA, nickel dichloride | 1.000 | 0 | Exogenous element | | |
|  | Leu-Val, TG, triacetylcellulose | 1.000 | 0 | Exogenous element | | |
|  | PTEN, VEGFA, arsenic atom | 1.000 | 0 | Exogenous element | | |
|  | PTEN, VEGFA,, copper(0) | 1.000 | 0 | Exogenous element | | |
|  | 5-fluorouracil, PTEN, VEGFA | 1.000 | 0 | Exogenous element | | |
|  | PTEN, VEGFA, leflunomide | 1.000 | 0 | Exogenous element | | |
|  | 5-aza-2’-deoxycytidine, PTEN, VEGFA | 1.000 | 0 | Exogenous element | | |
|  | PTEN, VEGFA, coumestrol | 1.000 | 0 | Exogenous element | | |
|  | Troponin, Troponin I, Ca2+ | 1.000 | 0 | Ambiguous element | | |
|  | PTEN, VEGFA, diclofenac | 1.000 | 0 | Exogenous element | | |
|  | PTEN, VEGFA, gemcitabine | 1.000 | 0 | Exogenous element | | |
|  | PTEN, VEGFA, rosiglitazone | 1.000 | 0 | Exogenous element | | |
|  | Niclosamide, PTEN, VEGFA | 1.000 | 0 | Exogenous element | | |
|  | PTEN, VEGFA, ochratoxin A | 1.000 | 0 | Exogenous element | | |
|  | Aerosols, PTEN, VEGFA | 1.000 | 0 | Exogenous element | | |
|  | IL18, PTEN, VEGFA | 1.000 | 0 | + | + | angiogenesis and hypertrophy |
|  | PTEN, VEGFA, reactive oxygen species | 1.000 | 0 | + | + | angiogenesis and hypertrophy |
|  | PTEN, VEGFA, thalidomide | 1.000 | 0 | Exogenous element | | |

| Truncated INDRA DB model, Page Rank | Members | Cliqueness | # of children | Possible | Relevant for HCM | Likely implication |
| --- | --- | --- | --- | --- | --- | --- |
|  | ATPase, Ca2+, Troponin | 1.000 | 0 | + | + | contraction |
|  | CALM, MYL12A, MYLK | 1.000 | 0 | + | + | contraction |

| Truncated INDRA DB model, Node Degree | Members | Cliqueness | # of children | Possible | Relevant for HCM | Likely implication |
| --- | --- | --- | --- | --- | --- | --- |
|  | ATPase, Ca2+, Troponin | 1.000 | 0 | + | + | contraction |
|  | CALM, MYL12A, MYLK | 1.000 | 0 | + | + | contraction |

| INDRA DB model, Page Rank | Members | Cliqueness | # of children | Posible | Relevant for HCM | Likely implication |
| --- | --- | --- | --- | --- | --- | --- |
|  | ATPase, Ca2+, filament, MYL12A, Troponin C, Troponin T | 0.600 | 0 | Ambiguous element | | |
|  | Ca2+, filament, MyHC 723, TNNI3, Troponin C, Troponin T | 0.600 | 0 | Ambiguous element | | |
|  | ATPase, Actin, Ca2+, filament, MYL12A, Myosin complex, Troponin, Troponin C | 0.679 | 0 | Ambiguous element | | |
|  | Actin, Ca2+, filament, MYL12A, Tpm1, Troponin C | 0.667 | 0 | Ambiguous element | | |
|  | Ca2+, ERVK-18, TPM1, Troponin C, Troponin T, tropomyosin | 0.600 | 0 | Exogenous element | | |
|  | DCM, MYBPC3, MYH7, TNNT1, TNNT2, Troponin C | 0.667 | 0 | + | +/- | dilated cardiomyopathy |
|  | CLEC3B, Ca2+, DCM, ERVK-18, TPM1, Troponin T | 0.667 | 0 | Exogenous element | | |
|  | CALM, CAMK2 complex, Ca2+, PKA, RYR2 | 0.700 | 0 | + | + | Ca-homeostasis |
|  | DCM, MYBPC3, MYH7, TTN, TnT, Troponin C | 0.600 | 0 | + | +/- | dilated cardiomyopathy |
|  | Ca2+, Troponin T, tropomyosin | 1.000 | 0 | + | + | contraction |
|  | Ca2+, EMD, TG, phenazine-1-carboxylate | 0.833 | 0 | Exogenous element | | |
|  | Actin, MYBPC3, TM, TnT, Troponin C | 0.700 | 0 | + | + | contraction |
|  | DES, DSP, PPP1R13L, PPP1R13L | 0.833 | 0 | + | +/- | arrhythmogenic right ventricular cardiomyopathy |
|  | CLEC3B, Ca2+, DCM, ERVK-18, FHC, Tpm1 | 0.600 | 0 | Exogenous element | | |
|  | CALM, Ca2+, MYL12A, MYLK | 0.833 | 0 | + | + | contraction |
|  | CLEC3B, Ca2+, filament, Tpm1 | 0.833 | 0 | Ambiguous element | | |
|  | MYL12A, Myosin complex, arm | 1.000 | 0 | Ambiguous element | | |
|  | MyHC723, Troponin C, Troponin T | 1.000 | 0 | + | + | contraction |
|  | TNNI3, Troponin C, Troponin T | 1.000 | 0 | + | + | troponin complex |
|  | Ca2+, PLN, SERCA2a | 1.000 | 0 | + | + | Ca-homeostasis |
|  | AICA ribonucleotide, AMPK, ATP | 1.000 | 0 | + | + | cell energy balance |
|  | Actin, ELC, MYL12A, Mhc, Myosin complex | 0.800 | 0 | + | + | contraction |
|  | DCM, MYBPC3, MYH7, TNNT2, TTN | 0.800 | 0 | + | +/- | dilated cardiomyopathy |
|  | Actin, DCM, TNNT1, TnI, Troponin C | 0.700 | 0 | + | +/- | dilated cardiomyopathy |
|  | Beta-MyHC, Ca2+, oxalic acid, SNCG | 0.833 | 0 | Exogenous element | | |
|  | Ca2+, PKA, RYR2, Troponin C | 0.833 | 0 | + | + | Ca-homeostasis |
|  | MYH, MYL12A, Myosin complex | 1.000 | 0 | + | + | myosin complex |

| INDRA DB model, Node Degree | Members | Cliqueness | # of children | Possible | Relevant for HCM | Likely implication |
| --- | --- | --- | --- | --- | --- | --- |
|  | ATPase, Ca2+, filament, MYL12A, Troponin C, Troponin T | 0.600 | 0 | Ambiguous element | | |
| * | ATPase, Actin, Ca2+, MYL12A, Myosin complex, Troponin | 0.867 | 2 | + | + | contraction |
| Child of * | ATPase, Actin, Ca2+, filament, MYL12A, MYL12A-A13T, Myosin complex, Troponin | 0.643 | 0 | Ambiguous element | | |
| Child of * | A13T, ATPase, Actin, Ca2+, MYL12A, Myosin complex, Tropomyosin, Troponin | 0.607 | 0 | + | + | contraction |
|  | Actin, Ca2+, filament, TNNT1, Troponin C, Troponin T | 0.600 | 0 | Ambiguous element | | |
|  | ATP, ATPase, Actin, MYH, MYL12A, Myosin complex, Troponin | 0.619 | 0 | + | + | contraction |
|  | Ca2+, ERVK-18, TNNI3, TPM1, Troponin C, Troponin T | 0.600 | 0 | Exogenous element | | |
|  | DCM, MYBPC3, MYH7, TNNT1, TNNT2, Troponin C | 0.667 | 0 | + | +/- | dilated cardiomyopathy |
|  | Actin, Ca2+, filament, MYL12A, Tpm1, Troponin C | 0.667 | 0 | Ambiguous element | | |
|  | DCM, MYBPC3, MYH7, TTN, TnT, Troponin C | 0.600 | 0 | + | +/- | dilated cardiomyopathy |
|  | CALM, CAMK2 complex, Ca2+, PKA, RYR2 | 0.700 | 0 | + | + | Ca-homeostasis |
|  | AICA ribonucleotide, AMPK, ATP | 1.000 | 0 | + | + | cell energy balance |
|  | DCM, MYBPC3, MYH7, TNNT2, TTN | 0.800 | 0 | + | +/- | dilated cardiomyopathy |
|  | Ca2+, EMD, TG, phenazine-1-carboxylate | 0.833 | 0 | Exogenous element | | |
|  | CLEC3B, Ca2+, DCM, ERVK-18, TPM1, Troponin T | 0.667 | 0 | Exogenous element | | |
|  | LBD6, OBSCN, TTN | 1.000 | 0 | ? | +/- | ? |
|  | Actin, DCM, TNNT1, TnI, Troponin C | 0.700 | 0 | + | +/- | dilated cardiomyopathy |
|  | CLEC3B, Ca2+, DCM, ERVK-18, FHC, Tpm1 | 0.600 | 0 | Exogenous element | | |
|  | CALM, Ca2+, MYL12A, MYLK | 0.833 | 0 | + | + | contraction |
|  | MYH, MYL12A, Myosin complex | 1.000 | 0 | + | + | myosin complex |
|  | Ca2+, PLN, SERCA2a | 1.000 | 0 | + | + | Ca-homeostasis |
|  | Ca2+, PKA; RYR2, Troponin C | 0.833 | 0 | + | + | Ca-homeostasis |
|  | TNNI3, Troponin C, Troponin T | 1.000 | 0 | + | + | troponin complex |
|  | Beta-MyHC, Ca2+, oxalic acid, SNCG | 0.833 | 0 | Exogenous element | | |
|  | Actin, Troponin I, activity | 1.000 | 0 | Ambiguous element | | |
|  | Actin, CTN, TM, TN, TnT | 0.700 | 0 | + | + | contraction |
|  | MyHC 723, Troponin C, Troponin T | 1.000 | 0 | + | + | contraction |
|  | CLEC3B, Ca2+, filament, Tpm1 | 0.833 | 0 | Ambiguous element | | |
|  | Actin, MYBPC3, TM, TnT, Troponin C | 0.700 | 0 | + | + | contraction |
|  | Actin, ELC, MYL12A, Mhc, Myosin complex | 0.800 | 0 | + | + | contraction |
|  | DES, DSP, PPP1R13L, PPP1R13L | 0.833 | 0 | + | +/- | arrhythmogenic right ventricular cardiomyopathy |
|  | MYL12A, Myosin complex, arm | 1.000 | 0 | Ambiguous element | | |
|  | Ca2+, Troponin T, tropomyosin | 1,000 | 0 | + | + | contraction |
