# Supplementary material for: Humans and machines in biomedical knowledge curation: hypertrophic cardiomyopathy molecular mechanisms’ representation
Source: BioData Min. 2021 Oct 2;14:45. doi: 10.1186/s13040-021-00279-2 (PMC8487578; doi:10.1186/s13040-021-00279-2)
Supplement: Supplementary file 4 — Additional file 4. Eidos statements. Statements extracted for the INDRA DB model by Eidos reading system. [file 13040_2021_279_MOESM4_ESM.docx]

**Additional file 4. Eidos statements**

| statement | number of extractions | nonsense |
| --- | --- | --- |
| Calcium(2+) activates calcium(2+). | 3 | no |
| AICA ribonucleotide activates AMPK. | 4 | no |
| MEK activates ERK. | 4 | no |
| Calcium(2+) activates transcription, DNA-templated. | 2 | no |
| ATP_synthase activates ATP. | 4 | no |
| AGT activates TGFB. | 3 | no |
| MEK activates MAPK3. | 1 | no |
| Glycogen activates lactate. | 1 | no |
| AAV9-based S15D-RLC mice activates intact cardiac function. | 1 | yes |
| AAV9-S15D-RLC therapy activates Disease. | 1 | no |
| AAV-S15D-RLC activates heart function observed. | 1 | yes |
| AAV9-S15D therapy activates Disease. | 1 | no |
| AAV virus A plasmid activates rAAV9-pseudotyped viral particles. | 1 | no |
| AVB activates proband received pacemaker. | 1 | yes |
| Cardiac phosphorylation activates heart function HCM-D166V mice. | 1 | no |
| Cytomegalovirus CMV activates S15D-RLC phosphomimic construct. | 1 | yes |
| Introducing S15D phosphomimic activates unique structural charge balance. | 1 | yes |
| Mutation activates deleterious TPM1 protein structure. | 1 | no |
| Mutations encoding sarcomeric contractile proteins activates Familial HCM. | 1 | no |
| Non-phosphorylatable D166V myocardium activates heart function HCM-D166V mice. | 1 | no |
| Plasmids activates rAAV9-pseudotyped viral particles. | 1 | no |
| PRKAG2 mutation activates PRKAG2 cardiac syndrome PS is inherited disease. | 1 | yes |
| Protein specific therapeutic targets activates quality life. | 1 | no |
| Pseudo-phosphorylation activates actomyosin function. | 1 | no |
| Pseudo-phosphorylation activates heart performance. | 1 | no |
| Pseudo-phosphorylation activates rescue HCM mice. | 1 | yes |
| Specific cell activates delivery specific organ. | 1 | yes |
| Structural activates molecular mechanisms. | 1 | yes |
| TPM1 mutations activates Heart Defects, Congenital. | 1 | no |
| Cytomegalovirus CMV activates human ventricular RLC carrying Ser-15 Aspartic acid mutation. | 1 | yes |
| Familial HCM is occurring inherited cardiac disease activates young athletes. | 1 | yes |
| D166V-elicited conformation RLC Ser-15 phosphorylation site activates cMLCK cardiac myosin motors phosphorylation. | 1 | yes |
| Cardiac-specific phosphomimic S15D-D166V inhibits functional structural histological observed HCM-D166V mice. | 1 | yes |
| Sirolimus inhibits MTOR. | 35 | no |
| MMP3 inhibits Collagen. | 2 | no |
| MMP3 inhibits FN1. | 1 | no |
| Mutation-induced phosphorylatable Ser-15 RLC site inhibits altered. | 1 | yes |
| Mutation-induced phosphorylatable Ser-15 RLC site inhibits biochemical perturbations. | 1 | yes |
| Phosphomimic delivery inhibits isovolumic relaxation constant Tau. | 1 | yes |
| S15D phosphomimic inhibits majority detrimental induced D166V mutation. | 1 | yes |
| Strain analysis images inhibits MICE. | 1 | yes |
| Protein specific therapeutic targets inhibits Disease. | 1 | no |
| MW molecular inhibits Performing 2D-electrophoresis. | 1 | yes |
