# Supplementary material for: Humans and machines in biomedical knowledge curation: hypertrophic cardiomyopathy molecular mechanisms’ representation
Source: BioData Min. 2021 Oct 2;14:45. doi: 10.1186/s13040-021-00279-2 (PMC8487578; doi:10.1186/s13040-021-00279-2)
Supplement: Supplementary file 5 — Additional file 5. Code used for generation of INDRA-assembled PubMed HCM model. [file 13040_2021_279_MOESM5_ESM.docx]

**Additional file 5. Code used for generation of INDRA-assembled PubMed HCM model**

from indra import literature

from indra.literature import pubmed_client

from indra.sources import reach

from indra.tools import assemble_corpus as ac

from indra.assemblers import cx

from indra.assemblers.cx.assembler import CxAssembler

hcm_search_products=literature.pubmed_client.get_ids('hypertrophic cardiomyopathy', use_text_word=True, major_topic=True, reldate=4000, mindate='2010/01/01')

hcm_paper_contents = {}

for hcm_search_product in hcm_search_products:

    content, content_type = literature.get_full_text(hcm_search_product, 'pmid')

    if content_type == 'abstract':

        hcm_paper_contents[hcm_search_product] = content

hcm_literature_stmts = []

for hcm_search_product, content in hcm_paper_contents.items():

    rp = reach.process_text(content)

hcm_literature_stmts += rp.statements

statements = hcm_literature_stmts

statements = ac.map_grounding(statements)

statements = ac.map_sequence(statements)

statements = ac.run_preassembly(statements, return_toplevel=False)

cxa = CxAssembler(statements)

cxm=cxa.make_model()

cxa_cx=cxa.save_model(file_name='hcm_pubmed.cx')
